# Comprehensive Mass Spectrometry Workflows to Systematically Elucidate Transformation Processes of Organic Micropollutants: A Case Study on the Photodegradation of Four Pharmaceuticals

**DOI:** 10.1021/acs.est.4c09121

**Published:** 2025-02-14

**Authors:** Rick Helmus, Ingrida Bagdonaite, Pim de Voogt, Maarten R. van Bommel, Emma L. Schymanski, Annemarie P. van Wezel, Thomas L. ter Laak

**Affiliations:** † Institute for Biodiversity and Ecosystem Dynamics, University of Amsterdam, Science Park 904, Amsterdam 1098 XH, The Netherlands; ‡ Amsterdam Institute for Life and Environment, Vrije Universiteit Amsterdam, De Boelelaan 1108, Amsterdam 1081 HZ, The Netherlands; § Analytical-Chemistry Group, van’t Hoff Institute for Molecular Sciences, University of Amsterdam, Science Park 904, Amsterdam 1098 XH, The Netherlands; ∥ Centre for Analytical Sciences Amsterdam, Science Park 904, Amsterdam 1098 XH, The Netherlands; ⊥ Amsterdam School for Heritage, Memory and Material Culture, Conservation and Restoration of Cultural Heritage, University of Amsterdam, P.O. Box 94522, Amsterdam 1090 GN, The Netherlands; # Luxembourg Centre for Systems Biomedicine, University of Luxembourg, 6 avenue du Swing, Belvaux L-4367, Luxembourg; ∇ KWR Water Research Institute, Groningenhaven 7, Nieuwegein 3430 BB, The Netherlands

**Keywords:** UV photolysis, advanced oxidation processes, in-line degradation, transformation products, non-target analysis workflows, open science

## Abstract

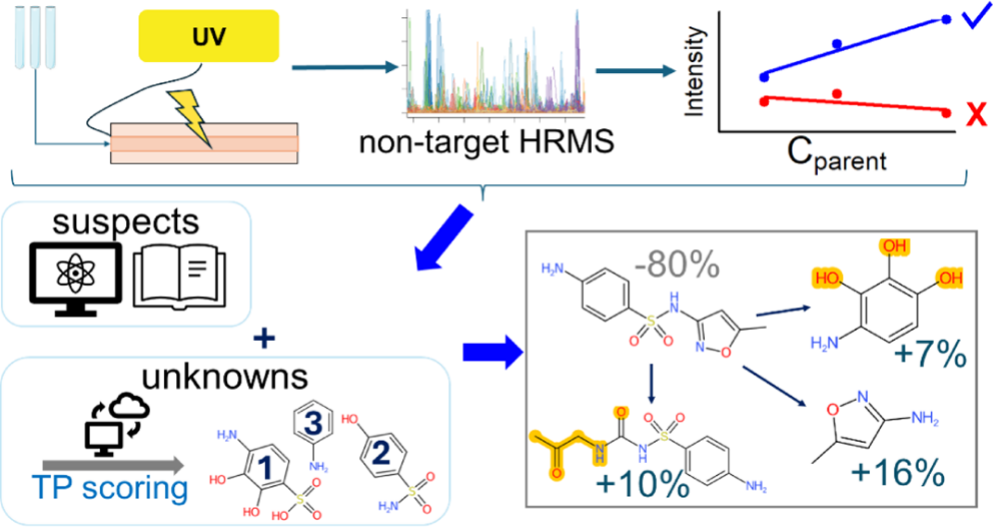

Organic micropollutants (OMPs) in the aquatic environment challenge conventional water treatment processes. Advanced oxidation processes, such as UV photolysis, serve as effective strategies to remove OMPs. However, these often yield unknown transformation products (TPs). High-resolution mass spectrometry (HRMS)-based non-target analysis (NTA) is commonly used to screen large numbers of chemicals but faces specific challenges such as low concentrations of compounds of interest, lack of reference standards, and the need for sophisticated data analysis workflows when used for TP identification. This article describes comprehensive workflows to study UV photolysis-related processes and the resulting TPs, by combining an automated photodegradation setup and HRMS and advanced NTA approaches. Four pharmaceuticals were successfully degraded in a case study, and 38 NTA features were effectively prioritized from complex sample matrices and identified as TPs through complementary approaches developed in this work. The identified TPs were structurally diverse and mostly novel. Semi-quantitation suggested that the TPs explained a relevant part of the parent removal. The developed workflows are a step toward systematic comprehensive analysis of transformation processes in water and beyond. The openly available data-processing tools and data enhance transformation data repositories and algorithms and support NTA studies in general.

## Abstract 2

We demonstrate tools and open-source workflows to systematically identify and exchange information about unknown chemicals formed from environmental pollutants during common oxidative water treatment processes.

## Introduction

Organic micropollutants (OMPs), such as pharmaceuticals and their transformation products (TPs), are pervasive in aquatic environments, with detected concentrations of ng/L to μg/L in drinking water sources.^1,2^ Advanced oxidation processes (AOPs) are applied for OMP removal in treatment of wastewater^3−5^ and drinking water.^6,7^ A commonly applied technique for the latter is UV photolysis.^8−11^ The UV emission bands align with the dissociation energies of, e.g., double bonds in organic compounds, facilitating their direct photolysis upon UV light absorption. Additionally, indirect photolysis can occur with reactive OH radicals formed when UV light interacts with purposely added reactants such as hydrogen peroxide (H_2_O_2_) or ozone.^8,12,13^ Natural organic matter (NOM) in water sources can inhibit or promote OMP removal,^14−16^ e.g., by shielding UV light and scavenging OH radicals in AOPs or the formation of reactive species, respectively. The nonselectivity of OH radicals and unpredictable interference of NOM can lead to the formation of various TPs with unknown toxicity.^10,17−23^ Understanding degradation pathways and systematically identifying TPs are therefore crucial for assessing environmental fate and ultimately their risks.

The identification of unknown or suspect OMPs in complex environmental matrices is currently typically performed with liquid chromatography coupled to high-resolution mass spectrometry (LC-HRMS). This offers exceptional sensitivity and the ability to identify compounds based on accurate mass, isotopic patterns, and characteristic molecular fragments.^24^ LC-HRMS is combined with non-target analysis (NTA) to screen and identify large numbers of chemicals.^24^ However, elucidating TPs with NTA presents several complications. First, multiple TPs can arise from a single-parent compound, while the same TP may be formed from multiple parents.^25,26^ Second, TPs generally have low concentrations, like their parents, and may be less sensitive to applied detection techniques than their parents. Third, the unavailability of reference standards hinders analytical method development and TP identification. Finally, data interpretation is often complex and tedious. The absence of MS^2^ library data for most TPs requires manual elucidation, which involves reviewing chromatograms, mass spectra, assigning identification confidence^27^ and possible pathways, and interpreting intensity trends across experimental conditions for numerous (often 100 s–1000 s) TP candidates, as performed by, e.g., Gulde et al.^28^ To alleviate some of these difficulties, the open-source software patRoon^29,30^ was recently enhanced with functionality for TP screening^31^ such as automatically obtaining and combining suspect data from various prediction and library sources, integrating approaches to recognize TPs and link these to corresponding parents, and improved identification of TPs that are typically absent in open data sources.

The primary objective of the present work was to establish and showcase a comprehensive analytical and computational workflow to systematically perform photodegradation experiments and elucidate the resulting TPs. Four environmentally relevant and light-sensitive pharmaceuticals (flecainide, metoprolol, sulfamethoxazole, and phenazone) were selected for automated photodegradation experiments using the “TooCOLD” setup.^32−34^ This setup was adapted to perform UV degradation experiments with and without H_2_O_2_ and NOM and coupled to LC-HRMS for direct postdegradation analysis. NTA was applied to investigate TP formation using open-source patRoon-based workflows that enhance the prioritization, elucidation, and reporting of TP data.

## Materials and Methods

### Materials

Flecainide acetate salt (≥98%), metoprolol tartrate (≥99%), sulfamethoxazole (≥98%), and phenazone (≥97.5%) were purchased from Sigma-Aldrich (Zwijndrecht, The Netherlands). Further details are in Section S1.1. Water was obtained from a Dutch drinking water treatment facility on May 16, 2022, which uses Lake IJssel as a source (see Table S2 for water characteristics). The sampling point was after rapid sand filtration and before the UV/H_2_O_2_ treatment. The water was subsequently filtered (GD/X, 25 mm, 0.45 μm, Whatman) and used to imitate natural background in degradation experiments from, e.g., dissolved organic matter and salts (hereafter referred to as “NOM”).

### Instrumentation

Photodegradation experiments were automated with the “TooCOLD” setup.^32−34^ In short, the TooCOLD setup is a fully automated system to study light-induced degradation and consists of automated sample introduction, an irradiation source with dedicated optics, and a light-exposure cell based on a liquid-core waveguide. This setup was adapted for the current study to reduce sample carryover, improve sample recovery, and include the use of UV (characteristic wavelength 254 nm) and H_2_O_2_ for sample degradation purposes (see Section S1.2). An LC-HRMS system was coupled for direct chemical analysis after degradation and consisted of an Acquity ultra-high-performance LC (Waters, Etten-Leur, The Netherlands) and a maXis 4G Q-TOF upgraded with an HD collision cell and equipped with an electrospray ionization source operating in positive mode (Bruker Daltonics, Leiderdorp, The Netherlands), see Section S1.3 for further details. The analytical repeatability was <5.0% standard error for all parent compounds, see Section S1.4.

### Degradation Experiments

The experiments were performed with mixtures of parent pharmaceuticals to simulate cooccurrence in environmental conditions. The degradation experiments occurred by 2 h exposure to (1) UV, (2) UV and 10 mg/L H_2_O_2_, or (3) UV, 10 mg/L H_2_O_2_, and NOM. The experiments were performed with initial parent concentrations of 25 μg/L, 75 μg/L, and 150 μg/L (*n* = 3). The relatively high concentrations compared with environmental occurrences were chosen to ensure comprehensive detection of potentially low-abundant TPs. Single-parent experiments were performed at 150 μg/L (*n* = 2) and were used to aid the identification of parent-specific TPs. In addition, mixture experiments were repeated with a disabled light source (“dark controls”) at 150 μg/L (*n* = 2) to assess the effects of adsorption processes and parent removal solely by H_2_O_2_/NOM. The experiments were repeated with 0 h exposure, which was used to correct for parent removal and perform NTA blank subtraction.

### Target Analysis

Target analysis was performed to quantitate the loss of parent pharmaceuticals and to semi-quantitate the formation of the TPs with (tentatively) assigned structure and available standards. The LC-HRMS data were processed with TASQ 2021 (Bruker Daltonics, Leiderdorp, The Netherlands). Quantitation of parent compounds was performed on the naturally occurring M + 2 isotope (i.e., the agglomeration of isotopes with equal nominal mass distanced two units away from the monoisotopic mass); the low abundance (<6%) allowed accurate quantitation of relatively high parent concentrations by avoiding detector saturation. The TPs were quantified by the MS signals from the monoisotopic mass. The analytes were confirmed by retention time (±0.1 min), accurate *m*/*z* (±5 mDa), and good isotopic pattern match (mSigma <100, calculated by the SigmaFit algorithm^35^ by TASQ, only performed if the intensity of the monoisotopic mass was below the detector saturation). The calibration curves ranged from 12.5 to 250 μg/L (parent compounds) and 1.2–150 μg/L (TPs), which were prepared by serially diluting the highest concentration standard in steps of two. The calibration lines for each analyte were ensured to have ≥5 points, an *R*^2^ of ≥0.99, and residuals of ≤30%. The significance of differences between 0 h and 2 h exposure and among degradation conditions was assessed with a pooled *t*-test assuming equal variances (*p* ≤ 0.05).

### Transformation Product Screening

**Figure 1:**
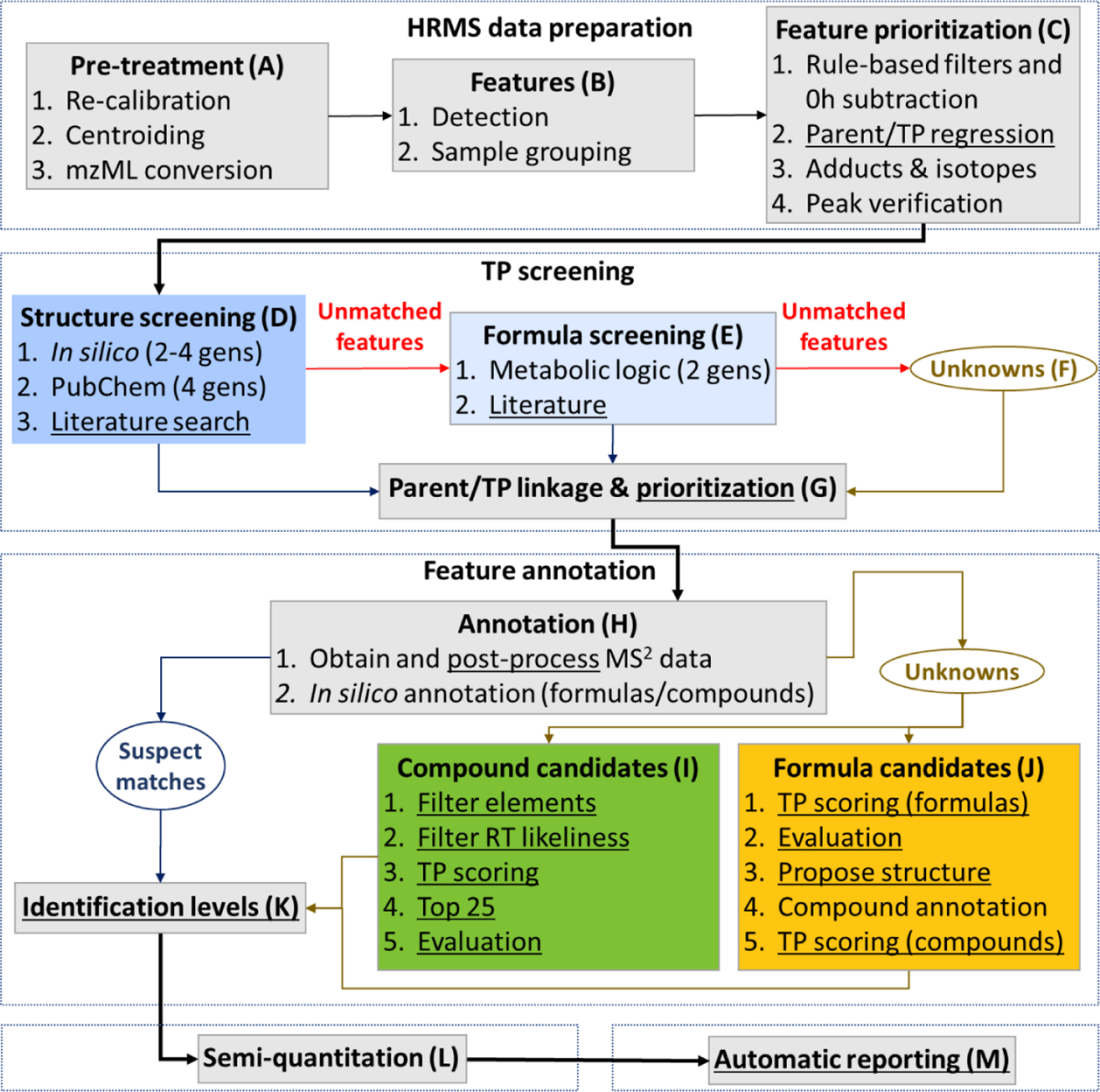
patRoon-based non-target analysis workflow to elucidate TPs. Underlined steps are novel or extended in this work. The box colors of steps D, E and I, J are reused in subsequent figures to identify these steps. Gens: number of prediction generations.

The formation of TPs was investigated with R^36^ using patRoon 2.3.3-based NTA workflows,^29−31,37^ which were extended with various novel steps (underlined in Figure 1) as described below. The code associated with this manuscript and a detailed overview of all software tools are available from ref (38).

Raw LC-HRMS data were first pretreated by internal *m*/*z* recalibration and centroiding and subsequently exported to the mzML format^39^ via DataAnalysis (Figure 1A). Next, features were detected, grouped, and aligned across samples via OpenMS^40^ (Figure 1B). The features were then prioritized with common patRoon functionality, including blank subtraction of 0 h experiments and regression analysis (Figure 1C). A linear relationship was assumed between the three initial parent concentrations tested in the mixture samples and the TP abundance (i.e., peak intensity). This is limited to TPs that originate from the studied parents, as the concentration of background chemicals across concentration points was unaltered. Thus, only features with a significant correlation were kept, yet with tolerant constraints to accommodate TPs with small linear deviations due to, e.g., detector or reactant saturation or higher-order kinetics. Further prioritization details and constraints are described in Section S1.5.

The prioritized features were elucidated by a thorough TP screening workflow. Several prediction algorithms and literature sources were aggregated to obtain TP suspects with known structure (“structure suspects”) or known formula (“formula suspects”), see Figure 1D,E and details in Section S1.6. Features that matched with a structure suspect were not further considered for formula suspect screening, as the structural data from the former allows more confident feature identification. The features that matched with structure suspects, formula suspects, or without any match (“unknowns”, Figure 1F) were each linked with parent features with the TP componentization functionality of patRoon (Figure 1G). Links were removed for TP features that were present in any of the single-parent experiments for other parents, unless the intensity was five times less compared to the mixture experiments or it was also present in the corresponding single-parent experiment.

Several steps were then performed to annotate the suspects (Figure 1H). Feature MS and MS^2^ data were automatically obtained with patRoon via mzR^41^ and postprocessed with a background removal algorithm developed in this work and patRoon filtering functionality (detailed in Section S1.7). The suspect hits were then subjected to formula annotation with GenForm^42^ and compound annotation with MetFrag^43^ using a compound database from the suspects, see Section S1.8. Identification confidence levels^27^ were assigned automatically and refined with reference standards where possible (further detailed in Section S1.9; Figure 1K).

The annotation workflow for unknowns included adjustments to accommodate a broad range of possible candidates. Compound candidates were obtained from the PubChem database,^44,45^ which, given its large size (119 million unique chemicals as of August 2024), maximizes the search range. First, compounds were filtered (Figure 1I steps 1–2) to exclude candidates with (1) elements unlikely to be present in TPs from the investigated parents (Cl, Br, Si, and P) or (2) unexpected LC elution order relative to the parent (with considerable tolerance to allow for predictive errors, see Section S1.10). Next, compound candidates were ranked by the “TP score”, which was derived from (1) the maximum “fit” in the parent molecule and *vice versa*, (2) the maximum structure similarity with structure suspects, and (3) *in silico* annotation similarity (fit_compound_, sim_suspects_, and ann_compound_, respectively, see details in Section S1.11). Formula candidates were similarly ranked by the formula fit and annotation similarity (fit_formula_ and ann_formula_, respectively, see details in Section S1.11). All ranking metrics were evaluated with suspect data, and these data were subsequently used to derive thresholds to eliminate unlikely candidates (see Section S1.11 and Figure S2). The candidates were then ranked, removed if below TP score thresholds or outside the top 25, and manually evaluated (Table S8) to obtain a final selection with plausible candidates (Figure 1I steps 3–5 and Figure 1J steps 1–2). Structures were proposed for candidates from formula annotations (Figure 1J step 3) and subsequently verified and scored by *in silico* compound annotation, which used the proposed structures as an input compound database (Figure 1J step 4). Finally, compound TP scores were calculated and further used to score TP candidates with proposed structures (Figure 1J step 5).

The workflow then concluded with semi-quantitation of the identified TPs and automatically reporting all workflow data (Figure 1L,M). Semi-quantitation was performed to estimate molar mass balances by postcomparison with standards for TPs if available, or otherwise patRoon via MS2Quant^46^ (detailed in Section S1.12). The data interpretation was aided by (1) the redesigned general reporting interface of patRoon and (2) a developed specialized reporting tool that automatically summarizes key properties and observations for each TP candidate, such as chromatograms, abundance in samples, and MS^2^ annotations (detailed in Report R1).

## Results and Discussion

### Degradation of Parent Compounds

**Figure 2:**
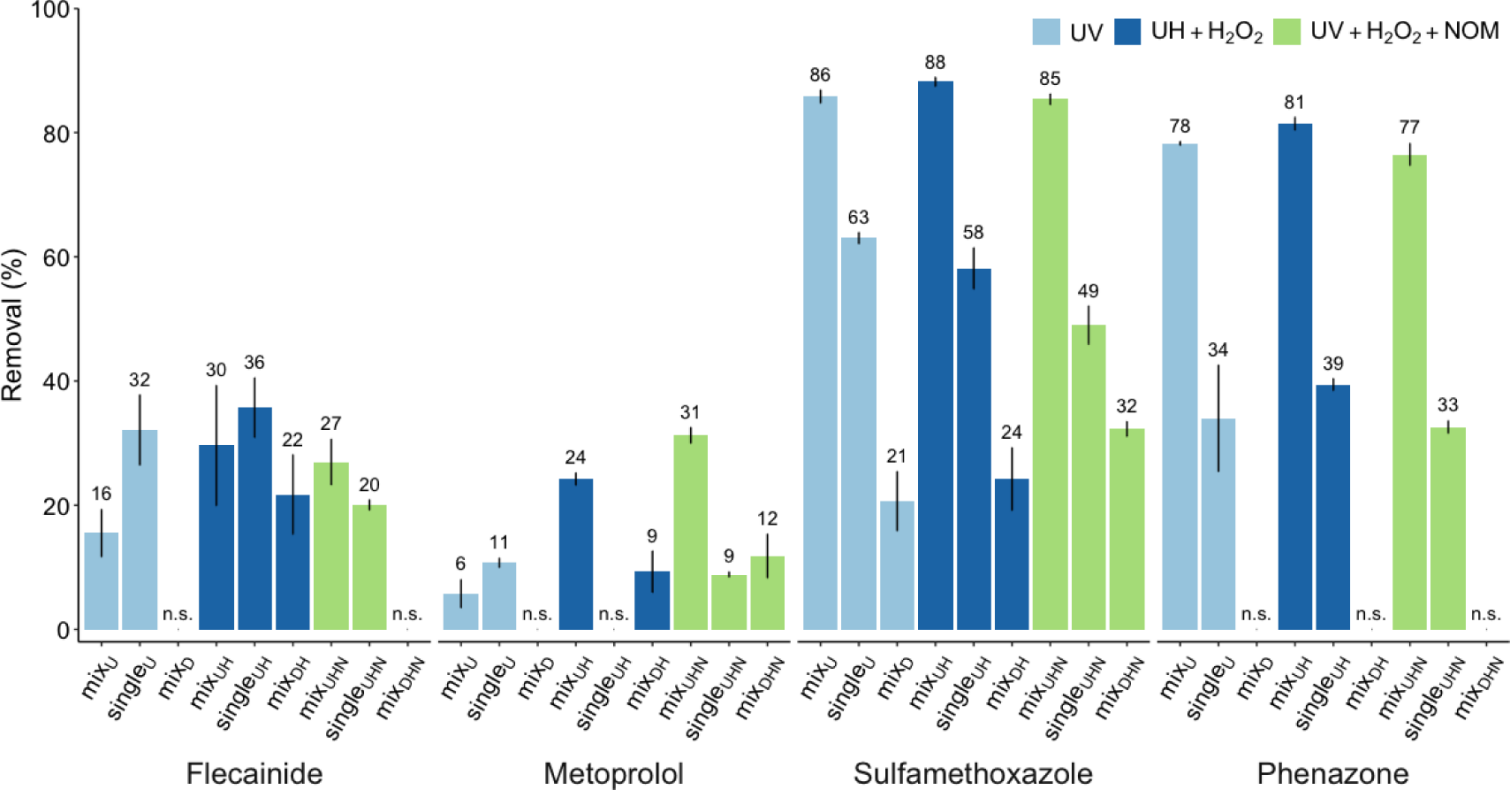
Percent removal of the tested parent compounds (150 μg/L) in experiments with parent mixtures, single-parent solutions, and dark controls, which were exposed to the tested degradation conditions. Results were normalized to 0 h exposure experiments. Error bars represent standard errors between replicates. n.s.: no significant removal (*p* > 0.05).

The removal of parent pharmaceuticals (normalized to 0 h exposure) after exposure to UV, UV and H_2_O_2_, or UV, H_2_O_2_, and NOM for the mixture experiments at 150 μg/L (mix_U_, mix_UH_, and mix_UHN_, respectively), single-parent experiments (single_U_, single_UH_, and single_UHN_, respectively), and dark controls (mix_D_, mix_DH_, and mix_DHN_, respectively) is summarized in Figure 2. The highest removal was observed in mixture experiments for sulfamethoxazole and phenazone (77–88%) and was similar under all photolytic conditions. The degradation of flecainide and metoprolol was much lower in mixture experiments (6–31%). Significantly lower removal for both parents was observed (*p* < 0.05) in mix_U_ (6–16%) than in mix_UH_ and mix_UHN_ (24–31%). The degradation of flecainide and metoprolol was significantly higher in single_U_, when compared to mix_U_ (*p* ≤ 0.05, ∼ 200% difference), indicating that other compounds hampered their direct UV photolysis. In contrast, the opposite was observed for sulfamethoxazole and phenazone in all treatments and for metoprolol in single_UH_ and single_UHN_ (*p* ≤ 0.05, ≥ 27% difference), suggesting that the presence of other compounds enhanced degradation. In dark controls, removal was observed for flecainide in mix_DH_ (22%), metoprolol in mix_DH_ and mix_DHN_ (9–12%), and sulfamethoxazole under all conditions (21–32%) but not for phenazone. The loss of sulfamethoxazole in mix_D_, i.e., where UV, H_2_O_2_, and NOM were absent, suggests that adsorption occurred during the 2 h residence time in the degradation cell. Hence, the actual degradation of sulfamethoxazole may be smaller. The degradation in mixture experiments at lower initial parent concentrations (Figure S4) was generally difficult to assess, as the relatively high variance among replicates often led to insignificant results or measurements were below quantitation limits. Nevertheless, the removal of metoprolol in mix_UHN_ was significantly lower (*p* ≤ 0.05) at 25 μg/L versus 75 and 150 μg/L (21%, 28%, and 31%, respectively), suggesting that the degradation rate of metoprolol was enhanced by increased initial concentrations.

The high removal of sulfamethoxazole and phenazone by direct photolysis,^47−49^ enhanced removal of flecainide and metoprolol by H_2_O_2_,^49,50^ enhanced removal of metoprolol in the presence of NOM,^51^ and the enhanced removal of sulfamethoxazole and other pharmaceuticals in mixtures,^52,53^ matches literature findings. However, the significantly enhanced degradation of sulfamethoxazole and phenazone by H_2_O_2_ and NOM reported in the literature^8,47,49,54−56^ was not observed in this study. Nevertheless, an exact comparison with the literature is difficult due to different reactant dosages, initial parent concentrations, sample matrices, and the unique geometry of the TooCOLD reaction cell with direct sample analysis. Regardless, the often significant removal of the parents demonstrates that the setup was successful in degrading test compounds under various conditions.

### Overview of Non-Target Analysis Results

**Figure 3:**
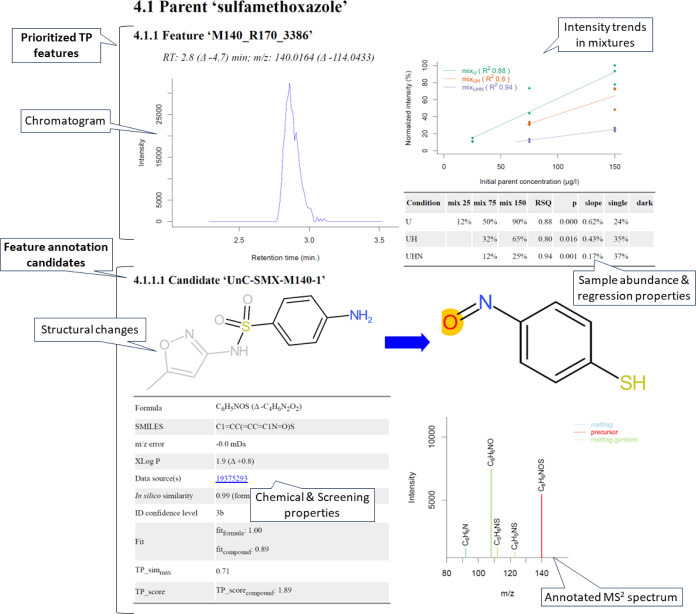
Example layout of the output from the automated reporting tool developed in this study for one TP of sulfamethoxazole. The complete report is provided in Report R1.

The key NTA results for all TP candidates were automatically summarized to ease data interpretation, see Figure 3 and Report R1. The obtained NTA data are summarized in Table 1. The next sections describe the NTA results in further detail.

**Table 1:** Overview of All Non-Target Analysis Results

	Workflow step^a^	Total	FLE	MET	SMX	PHE
Features	Sample grouped (B)	18,608	-	-	-	-
	Prioritized (C)	78	-	-	-	-
Suspect TP screening	Total structure suspects (D)	676^b^	135	218	180	154
	Matched structure suspects (D)	52^b^	3	35	13	2
	Total formula suspects (E)	228^b^	114	83	116	93
	Matched formula suspects (E)	1^b^	0	1	0	0
	Total matched features	27^b^	3	14	9	2
	Total parent/TP links^c^ (G)	67	5	45	15	2
Screening for unknown TPs	Unknown features (F)	51	-	-	-	-
	Unknown parent/TP links^c^ (G)	131	27	26	29	49
	Total compound candidates (I)	87,493	29,046	28,065	33,704	67,211
	Prioritized compound candidates (I)	7	0	0	4	3
	Total formula candidates (J)	314	145	144	152	239
	Prioritized formula candidates (J)	7	1	0	0	6
Identified TPs	Level 1: confirmed structure (K)	4^d^	0	0	2^d^	2^d^
	Level 2: probable structure (K)	0	0	0	0	0
	Level 3: tentative structure (K)	33	0	17	13	3
Semi-quantitative mass balance^e^	With standards (L)	-	-f	5–29%	3–18%^g^	
	MS2Quant prediction (L)	-	0–41%	12–123%	10–75%	4–35%
	Total (L)	-	0–41%	19–152%	18–59%^g^	

a See (Figure 1a).

b Total of unique suspects or features.

c Unique for each parent, TP feature, and suspect (except unknowns).

d Of which one TP was formed by both sulfamethoxazole and phenazone.

e Ratio TP concentration versus parent removal (molar, across experimental conditions).

f No standard available.

g Results were mean averaged since the formation of one TP could not be distinguished from its two parents; FLE: flecainide; MET: metoprolol; SMX: sulfamethoxazole; PHE: phenazone.

### Detected Features and Feature Prioritization

**Figure 4:**
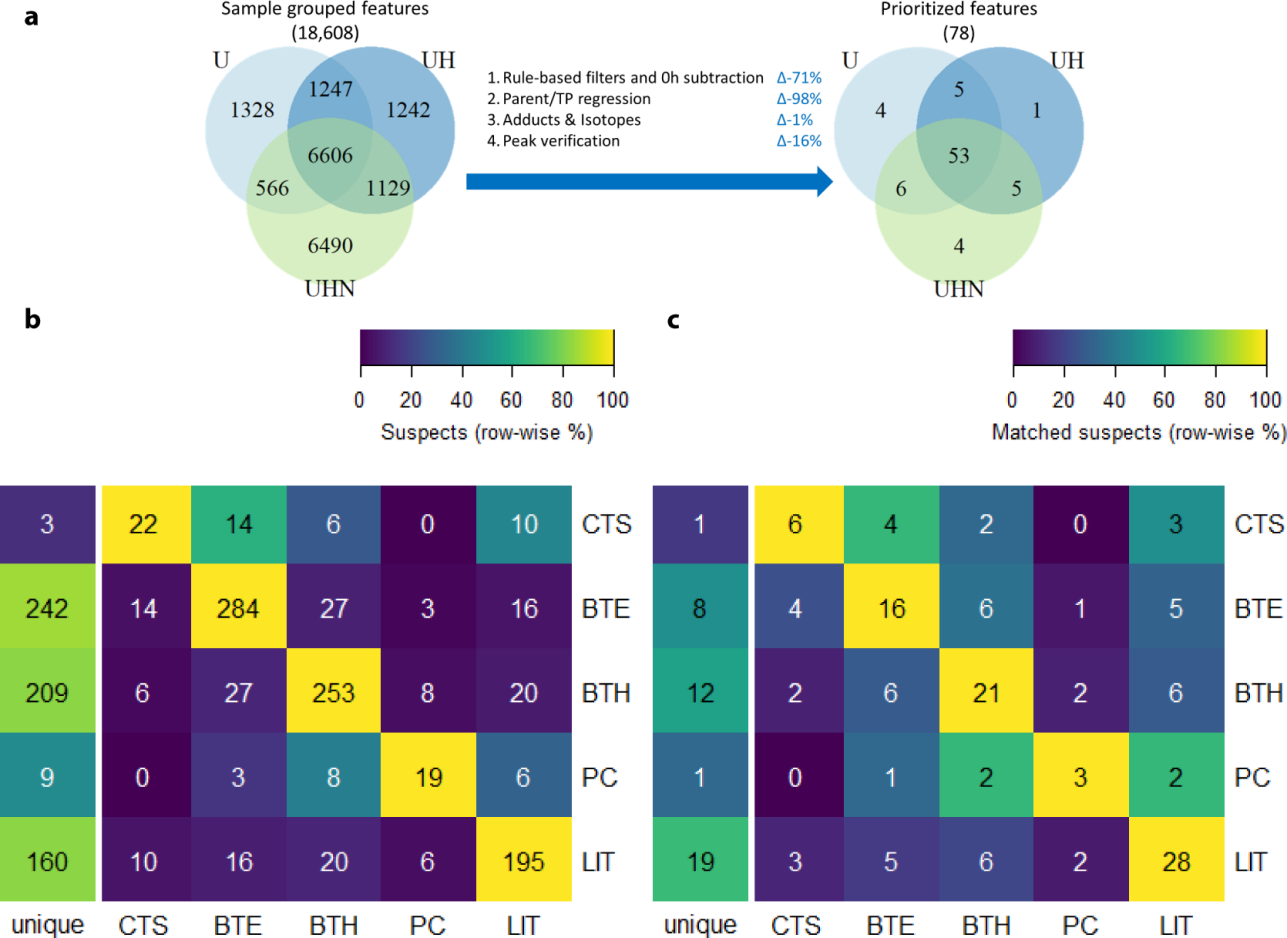
(a) Feature distributions in UV (U), UV and H_2_O_2_ (UH), and UV, H_2_O_2_, and NOM (UHN) experiments (excluding parents) before/after the employed prioritization steps. (b, c) Overlap of suspects (b) and matched suspects (c) between data sources and unique to the data source. The data sources are the Chemical Transformation Simulator (CTS), BioTransformer with environmental, or “allHuman” reaction libraries (BTE and BTH), PubChem (PC), and literature search (LIT).

A total of 256,784 features (excluding parents) were detected and reduced to 18,608 by sample grouping and alignment (Figure 4a). Through subsequent prioritization steps, ∼99% of these features were removed, with the largest decrease occurring through regression analysis (Figure 4a). The remaining 78 features showed a predominantly high correlation with the initial parent concentration (median *R*^2^ of 0.88 for features present at all initial parent concentrations, see Figure S5), yet included a feature with considerable linearity deviations due to MS signal saturation (see M150_R528_2607 in Report R1). Thus, the prioritization steps employed were highly effective in isolating features of interest, even in complex sample matrices with numerous features such as NOM and after the subtraction of 0 h experiments. Furthermore, the number of features prioritized automatically was sufficiently low for manual peak verification (93), and the subsequent manual elimination of features was limited (16%).

The presence of the prioritized features mostly overlaps in all photolytic conditions and in mixture and single-parent experiments (both 53, see Figures 4a and S6), while a minority of features were also detected in dark controls (10, see Figure S6). The discrepancies among degradation conditions are discussed further in the “Identified transformation products” section.

### Suspect TP Screening

From the 676 structure suspects and 228 formula suspects, 53 suspects were matched to 27 features, resulting in 67 unique links between the parent, TP feature, and suspect (see Table 1).

The number of structure suspects across data sources was similar for all parents (Figure S7), except for the literature search (LIT) for flecainide, illustrating that environmental transformations for this compound have been studied less frequently. Most TP suspects were matched to metoprolol and sulfamethoxazole. Of all the suspects, most originated from BioTransformer^57^ with environmental (BTE) or “allHuman” (BTH) reactions and LIT (195–284), see Figure 4b. Most of these (160–242) had one unique source, which demonstrated the complementarity of these data sources. Only a minority (16–28) of the suspects from these data sources could be matched to features (Figure 4c). This is possibly due to different transformation mechanisms, which were biological for BTE and BTH and primarily highly energetic AOP for LIT (Table S13). Furthermore, nearly all TPs from LIT were only tentatively identified and may occasionally include errors (see Table S13). Thus, the use of these data sources indicates the need for suitable prioritization and identification workflows to exclude false positives. Relatively few suspects were from the Chemical Transformation Simulator^58^ (CTS) and PubChem transformations^59−61^ (PC). This is likely a result of relatively small and specialized reaction libraries and an incomplete database, respectively. The structure suspect screening results demonstrate the complementarity of using different predictive (CTS/BTE/BTH) and literature (PC/LIT) approaches based on both biological and abiotic processes. Furthermore, the observed gap between predictive and literature data sources indicates the need to improve prediction algorithms and TP information in open resources.

### Screening for Unknown TPs

The 51 features without a suspect match were linked into 131 unique parent/TP pairs (see Table 1). Subsequent feature annotation resulted in ∼30,000–70,000 compound and 27–82 formula candidates for the unknowns linked to each parent. These were reduced by 1–2 orders of magnitude through the various prioritization steps developed in this study (Table 2) which enabled further manual processing. For 6 out of 7 formula annotation candidates, a structure could be proposed manually, and in total, 14 candidates could be assigned to unknown features (further discussed in the next section).

**Table 2:** Prioritization of the Candidates from the Compound and Formula Annotation Workflows for Unknown Features^a^

	Compound candidates				Formula candidates			
Prioritization	FLE	MET	SMX	PHE	FLE	MET	SMX	PHE
Raw	29,046	28,065	33,704	67,211	82	27	55	49
Element filter	25,881	24,900	30,426	61,978	-	-	-	-
RT filter	12,298	21,341	28,181	56,195	-	-	-	-
fit_formula_	7,102	13,500	1,611	48,003	23	11	8	24
fit_compound/_sim_suspects_	2,329	2,006	382	4,831	-	-	-	-
Top 25 per feature	143	175	100	375	1	0	0	6
Manual evaluation	0	0	4	3	1	0	0	6

a FLE: flecainide; MET: metoprolol; SMX: sulfamethoxazole; PHE: phenazone.

The unique parent chemical properties seemed to influence the effectiveness of the different prioritization steps. For instance, the RT filter was most effective for flecainide and led to ∼50% reduction, since its relatively high log P (4.5 versus 1.2–1.6 for other parents, calculated with rcdk)^62^ could be more easily discerned from log *P* values of TP candidates. The thresholds for the parent similarity metrics (see Figure S2) decimated compound candidates for sulfamethoxazole because of the presence of sulfur and phenazone due to its compact structure with a pyrazoline ring.

The selected annotation candidates were diverse and complementary to the suspect screening results. Most candidates had relatively low structural similarity to suspects (Figure S8). The selection also included candidates with low parent similarity but high identification confidence and high parent similarity but poor annotation confidence (e.g., UnF-PHE-M149-1 and UnC-PHE-M219-1, respectively, see Report R1). The latter also shows successful prioritization of features without MS^2^ data, a typical phenomenon for features with low intensity.

### Overview of Identified Transformation Products

In total, 38 features were matched with 66 structures and two formulas, resulting in 81 unique feature/candidate TP pairs (hereafter “TP candidates”). These are detailed in Report R1, and their identification levels^27^ are summarized in Figure S9. Confirmation with reference standards (see Table S10) led to the assignment of four TP candidates with level 1 (with one structure assigned to two parents), and two candidates matched well with standards but could not be distinguished from structurally close isomeric suspects (level 3a). Thirty-one of the remaining TP candidates were assigned a tentative structure (level 3b-d). MS^2^ library spectra were only available for candidates UnC-SMX-M140-1 and UnF-PHE-M149-1, and candidates SuS-SMX-M110-1, UnC-PHE-M375-1, and UnF-PHE-M150-1 matched well with *in silico* MS^2^ annotation but were disproved by reference standards. This highlights the need for reference standards to confirm TP candidates. Below, the discussion is limited to TP candidates with identification level 3 or better for metoprolol, sulfamethoxazole, and phenazone due to the large number of candidates assigned to these parents.

**Figure 5:**
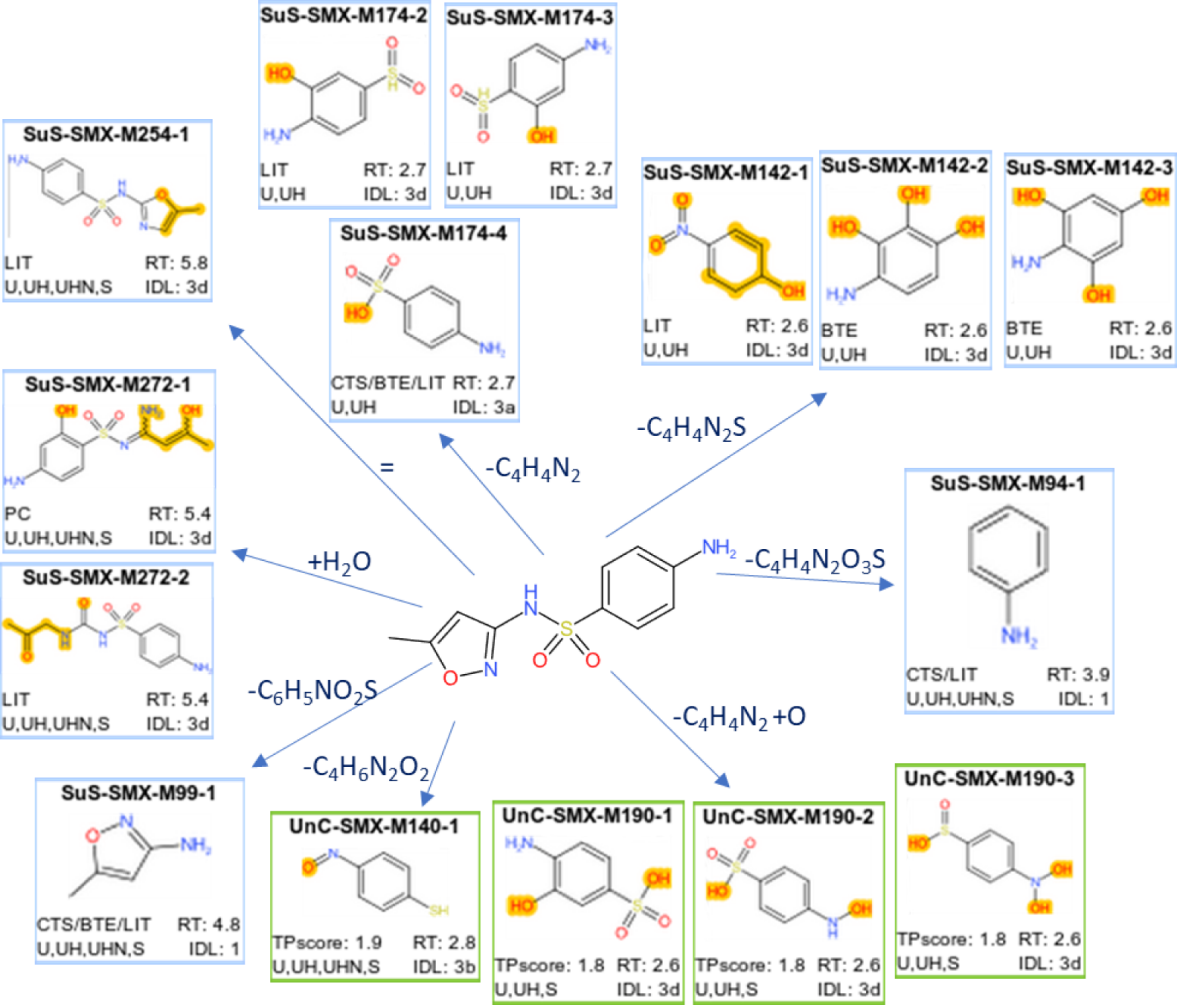
Overview of candidate TPs for sulfamethoxazole (identification confidence level 3 or better). The box color signifies whether the candidate was obtained by structure suspect screening (blue) or screening for unknown TPs with the compound annotation workflow (green). Each candidate is named by a unique TP identifier that refers to Report R1. The yellow shades in the TP structures represent additions or changes in atoms or bond order compared to the parent. Box annotations: CTS, BTE, LIT: suspect from Chemical Transformation Simulator, BioTransformer with environmental reaction library and literature, respectively; U, UH, UHN: present in mix experiments exposed to UV, UV and H_2_O_2_, and UV, H_2_O_2_, and NOM, respectively; S: present in any of the single-parent experiments; RT: retention time (minutes); IDL: identification confidence level, see Section S1.9.

TP candidates could be assigned for all four parent pharmaceuticals (Figures 5 and S10). The 15 candidates for sulfamethoxazole were structurally diverse (Figure 5), two of which had an identification confidence level 1 (SuS-SMX-M94-1, aniline and SuS-SMX-M99-1, 3-amino-5-methylisoxazole) and one level 3a (SuS-SMX-M174-4, sulfanilic acid). The candidates were primarily variations of substructures with the benzoic moiety, yet one TP candidate was a substructure with a five-membered azole ring (SuS-SMX-M99-1). Six candidates were novel, two of which stemmed from predictions (BTE) and four from screening for unknown TPs. For *phenazone*, two out of five TP candidates were assigned level 1, and all TPs resulted from the opening or removal of the five-membered ring (Figure S10a). Aniline (SuS-PHE-M94-1), also assigned to sulfamethoxazole (as SuS-SMX-M94-1), was the only TP candidate from the literature; one was found from CTS predictions and the remaining three from screening for unknown TPs. The TPs of metoprolol were mostly with small elemental changes, resulting in many isomeric TP candidates with tentative structure assignments at best (see Figure S10b). Nevertheless, one TP candidate (SuS-MET-M226-3, 1-amino-3-[4-(2-methoxyethyl)phenoxy]propan-2-ol) matched well with a reference standard and had confidence level 3a. All assigned structures originated from the literature. In addition, a novel metoprolol + H_2_O_2_ formula suspect calculated with metabolic logic was assigned to two features (detailed in Section S2.6). For flecainide, three TP candidates were found (see Figure S10c), but with poor identification confidence (level 4/5) due to the absence of MS^2^ data. However, the TPs resulting from the loss of a C_2_HF_3_ group (SuS-FLE-M333-1 and SuS-FLE-M333-2) may be plausible, since both isomers were predicted by all three algorithms, could be linked to two different features by accurate *m*/*z* (M333_R474_5934 and M333_R393_6915), and were identified in single-parent experiments.

Most of the features from the TP candidates were found in single- and mixed-parent experiments and all treatments, except for sulfamethoxazole TPs (see Figures 5, S10 and S11). The features that were not omnipresent were often close to detection limits or workflow thresholds. This might explain their scattered observations over the experiments and complicates the qualitative assessment of the influence of experimental conditions on transformation pathways. Regardless, for sulfamethoxazole, most TP candidates were detected in all treatments except in the presence of NOM. This could result from the NOM matrix that partially suppressed HRMS detection or indicate alternative transformation pathways, since the removal of sulfamethoxazole in NOM appeared unaffected (see Figure 2). In addition, the aforementioned metoprolol + H_2_O_2_ TP candidate was detected only in experiments with H_2_O_2_, as expected. Only one TP candidate, metoprolol + O, was found in dark controls.

### Semi-Quantitative Mass Balances

**Figure 6:**
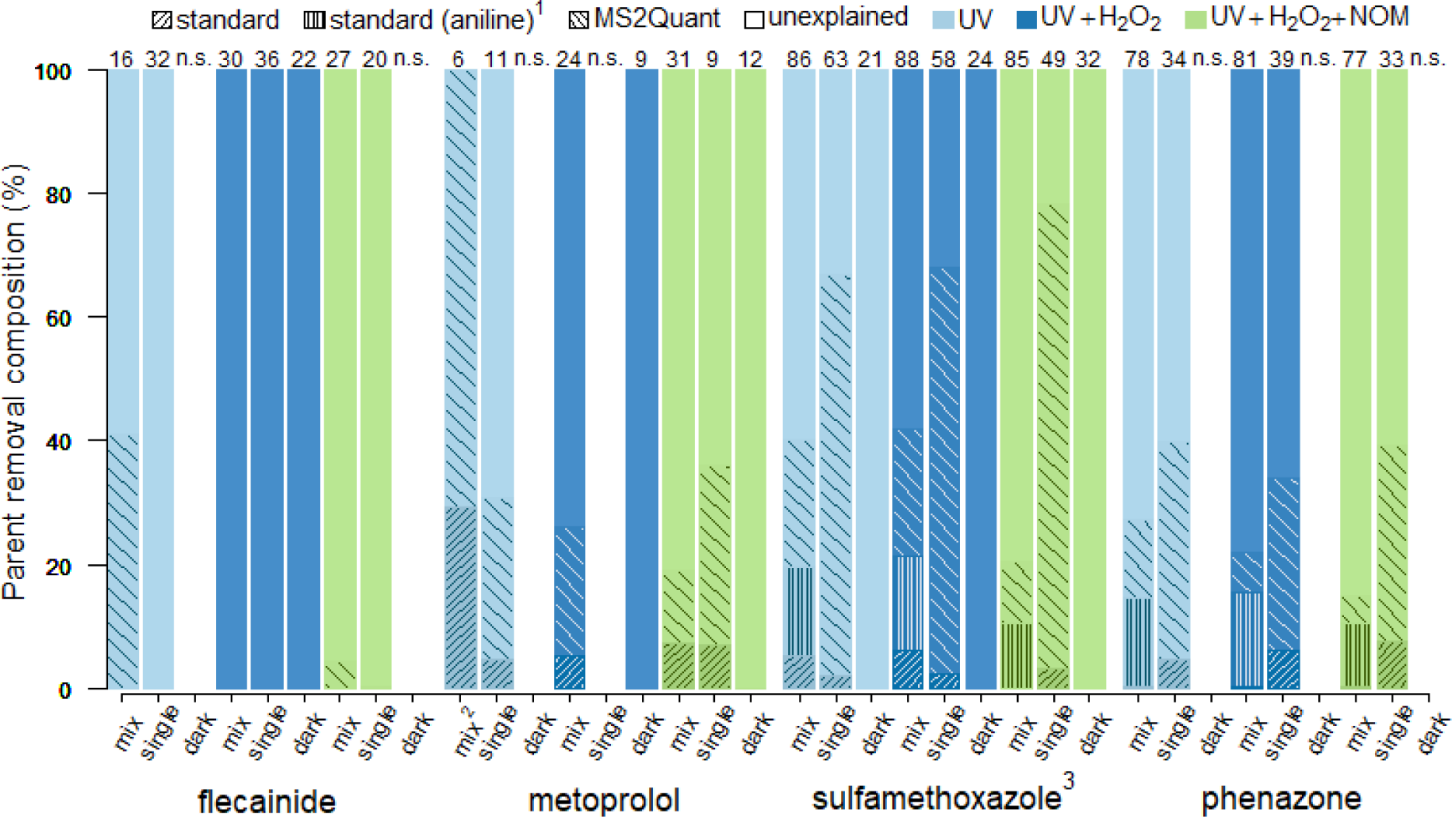
Composition of parent removal explanations from semi-quantitated TPs with standards or MS2Quant predictions. The numbers on top of the bars represent the parent removals (%). n.s.: no significant parent removal; ^1^: total formation of aniline from sulfamethoxazole and phenazone in mixture experiments; ^2^: the value for MS2Quant (123%) is cutoff; ^3^: formation of SuS-SMX-M99-1 (1–17%) was excluded as it could be formed simultaneously with other TPs in the same transformation reaction.

Molar mass balances were estimated for the TPs selected in the previous section by semi-quantitation, see Figure 6, Table S11 and Table S12. However, the results discussed here should be considered indicative due to the tentative structure assignment for most TPs, the prediction errors of MS2Quant that roughly cover a factor of 5,^46^ lack of matrix effect corrections, and other limitations discussed in Section S1.12. A large part of the removal of flecainide in mixture experiments exposed to UV (42%) appeared to be explained by SuS-FLE-M147-1 (see Table S12), while removal in other experiments was left mostly unexplained (<5%). The excess of explained removal for metoprolol in mixture experiments exposed to UV (total value ∼150%) was likely due to the low parent removal (6%) which complicates accurate quantitation. The explained removal for other experiments was lower (19–36%), and single-parent experiments exceeded mixture experiments considerably (36% vs 19%) when treated with UV, H_2_O_2_, and NOM. The explained removal for sulfamethoxazole and phenazone was similar across conditions and higher in experiments with single parents than mixtures (67–78% vs 10–27% and 34–39% vs 4–13% for sulfamethoxazole and phenazone, respectively). The transformation into aniline (SuS-SMX-M94-1/SuS-PHE-M94-1) appeared lower in single-parent experiments (2–7% vs 10–15%), but since aniline was formed from sulfamethoxazole and phenazone, this complicates the assessment of the mass balance in mixture experiments. The discrepancies between single-parent and mixture experiments suggest that transformation pathways for metoprolol, sulfamethoxazole, and phenazone were altered in the presence of other OMPs. The single TP candidate detected in dark controls exposed to H_2_O_2_ and NOM (metoprolol + O) only explained a very minor fraction of the metoprolol removal (<0.1%). Despite the uncertainties in the prediction of TP concentrations, the semi-quantitative mass balances mostly fall within 100%, and a relevant fraction of the removal could be explained by the identified TPs for most experiments and parents.

### Limitations and Future Perspectives

This work demonstrated an approach with automated sample introduction, degradation, LC-HRMS analysis, and thorough NTA screening and prioritization workflows to degrade selected pharmaceuticals under various photolytic conditions and elucidate the resulting TPs. The 81 TP candidates were structurally diverse, of which 45 were novel, 33 could be identified with a tentative structure (level 3), and 4 with a confirmed structure (level 1). The screening for unknowns, an approach developed in this work, successfully prioritized 14 out of the 81 TP candidates, including 4 with poor or no MS^2^ data. Semi-quantitation of the TPs indicated only partially complete mass balances under all studied conditions for all the parent compounds, and out of the 78 prioritized features, a considerable number remained without (tentative) structure assignment (20) or were unrecognized by any of the TP screening approaches (40). Part of the “missing” TPs may be undetected by the applied analytical methodology or due to low abundance or MS sensitivity, which can vary significantly compared to parents.^63−66^ For example, ∼40% of the features were absent in the experiments with the lowest initial parent concentration. The presence of salts and complex organic matter in the NOM experiments could suppress LC-HRMS detection, although this was not observed for the parents, and most of the TPs were also detected in NOM. The NTA workflow may also yield false negatives. For instance, features were prioritized with only a simple linear relationship between intensity and parent concentration (albeit with tolerant constraints). More sophisticated models might be required to prioritize TPs that excessively deviate from this linearity, e.g., when parent concentrations are sufficiently high to induce higher-order kinetics or a saturation of reactants. Furthermore, the thresholds utilized for screening of unknown TPs could result in a minority of false negatives (see Section S1.11) and might exclude TPs with highly dissimilar structural properties compared to their parent and suspect TPs thereof. In addition, the annotation workflows excluded candidates with additional incorporated halogens, which could be of interest for, e.g., certain compound classes or saline environments.^67−70^ Nevertheless, relaxing the stringent criteria applied here may drastically increase the number of tentative TPs for manual review, considerably increasing the manual workload. Current limitations in algorithms for feature detection^71−73^ ultimately necessitate manual review to eliminate false positives. Thus, for studies with a larger number of parents (e.g., 10 s–100 s), the inclusion of additional prioritization strategies may be necessary in the future to eliminate features outside the NTA chemical space,^74,75^ with low estimated identification levels,^76,77^ predicted concentrations, or environmental toxicities^78,79^ or poor chromatographic peak quality metrics.^72^

Several adjustments to this methodology can aid future research to further elucidate the transformation pathways of the OMPs studied here and beyond. The degradation setup could be improved with higher UV transmission (see Section S1.2), which may shorten degradation times, and the intensified conditions could reveal TPs with otherwise low abundance. In addition, experiments with increasing exposure times could be performed to obtain time profiles of TPs, which could reveal more of the intermediate TPs, allowing a more detailed study of transformation pathways and kinetics and potentially strengthening the proof of parent/TP relationships. Advanced analytical techniques can be applied to further improve the detection and identification of TPs with diverse physicochemical properties, such as large volume injection with online SPE, orthogonal chromatography with HILIC,^80^ complementary MS ionization techniques and MS^2^ techniques,^81−83^ and ion mobility coupled to HRMS to enhance analyte separation and identification.^84^ Moreover, adjustment of mobile phase pH and application of negative ionization MS could improve the detection of acidic TPs, which ionize differently than the predominantly basic parents studied here. Finally, more standards could be acquired or synthesized for dedicated target analysis to improve TP identification confidence and quantitation and allow more accurate prioritization and risk assessment of the identified TPs.

The large number of TPs identified for only four OMPs in this work underscores the potential relevance of TPs to the environment. Furthermore, the results suggest that parent removal and transformation pathways are influenced by the presence of other OMPs and possibly by NOM, emphasizing the importance of a relevant chemical background in degradation experiments. The approach demonstrated in this work enables researchers to systematically perform comprehensive transformation studies and identify TPs in complex samples and helps to move beyond sole characterization of parent chemicals in environmental monitoring, fate studies, optimization of water treatment processes, subsequent risk assessment, and chemical registration such as REACH.^85^ With relatively simple hardware modifications, it is envisioned that other types of degradation could be studied, such as ozonation and chlorination in water treatment and natural attenuation by solar irradiation in surface waters. Furthermore, the setup allows direct LC-HRMS measurements after a degradation experiment, consequently allowing elucidation of short-lived TPs that are likely missed with “conventional” offline experiments.^86^ The demonstrated workflows are not limited to water treatment processes, as studied here. They can be easily adopted to study other degradation processes in water treatment or the environment and other research domains such as food and art research. This was, for example, recently demonstrated for food ingredients (vitamins) and natural and synthetic dyes.^32−34,87^

The presented data processing workflows are expected to be widely applicable for non-target analysis studies. For instance, the approaches to prioritize and identify unknown TPs, perform semi-quantitative mass balances, and automatically report all workflow data can be used separately or in different combinations and do not rely on the TooCOLD degradation setup or experiments with increasing parent concentration. This work demonstrated the effectiveness of patRoon to easily aggregate TP suspects from various sources (even from unrelated pathways) to increase the numbers of detected and identified TPs. The data processing tools developed in this work are openly available and are currently being integrated within patRoon, which further assists in providing FAIR data to the community.^88^ Consequently, broader adoption could increase the availability of TP data to enhance openly available TP libraries and provide insights to improve prediction algorithms, thereby closing the gap between both that was observed in this study. In addition, the new data can enhance MS^2^ libraries and therefore assist future TP identifications in, e.g., future environmental monitoring studies. The TPs confidently identified in this work have been submitted to the PubChem transformation database^61^ and MassBank Europe,^89^ and we hope that future users will consider doing the same.

## Data Availability

The code associated with this manuscript is hosted on GitHub (https://github.com/rickhelmus/TC_photodeg) and archived on Zenodo (DOI: 10.5281/zenodo.14671663).

## Supporting Information Available

Additional details on Methods and Materials (Section S1), Results and Discussion (Section S2), Tables S1–12: additional information on chemicals used for target analysis; chemical parameters from the NOM sampling point; feature prioritization constraints; elemental transformations for formula suspect calculation; example MS^2^ cleanup by formula annotations; rules for identification confidence level assignment; ranking metrics for candidates from screening of unknowns; manual elimination criteria for implausible TP candidates from unknown screening workflows; semi-quantitation model parameters; results of TP confirmations with standards; semi-quantitative mass balance obtained by standards approach; semi-quantitative mass balance obtained by MS2Quant approach, and Figures S1–12: example MS^2^ cleanup by formula annotations; evaluation results of ranking metrics for TPs from screening of unknowns; response factors versus predicted ionization efficiencies; parent removal in mixture concentrations at 25, 75, and 150 μg/L; histogram of observed correlation coefficients between feature intensity and initial parent concentration; distribution of prioritized features across experiments; number of (matched) suspects across parents and suspect data sources; observed ranking metrics for the candidates from screening of unknowns; histogram of assigned identification confidence levels; overview of TP candidates for phenazone, metoprolol, and flecainide; feature intensities of identified TPs across experimental conditions; chromatogram for metoprolol + H_2_O_2_ TP candidates (PDF)
Report R1: automatically generated report with technical details of its creation and an overview of chromatograms, spectra, and other chemical and identification properties for the TP candidates from this study (PDF)
Table S13: overview of all transformation products found in this study through literature search (XLSX)
NTA_SRT: NTA study reporting tool^90^ (XLSX)
