# Supplementary material for: Comprehensive Mass Spectrometry Workflows to Systematically Elucidate Transformation Processes of Organic Micropollutants: A Case Study on the Photodegradation of Four Pharmaceuticals
Source: Environ Sci Technol. 2025 Feb 14;59(7):3723–36. doi: 10.1021/acs.est.4c09121 (PMC11866921; doi:10.1021/acs.est.4c09121)
Supplement: Supplementary file 1 — es4c09121_si_001.pdf [file es4c09121_si_001.pdf]

Supporting Information:

Comprehensive mass spectrometry workflows to systematically elucidate transformation processes of organic micropollutants: a case study on photodegradation of four pharmaceuticals

Rick Helmus‡*[1], Ingrida Bagdonaite‡[1,2,3,4], Pim de Voogt[1], Maarten R. van Bommel[3,4,5], Emma L. Schymanski[6], Annemarie P. van Wezel[1], Thomas L. ter Laak[1,7]

[1] Institute for Biodiversity and Ecosystem Dynamics, University of Amsterdam, Science Park 904, 1098 XH Amsterdam, the Netherlands
[2] Amsterdam Institute for Life and Environment, Vrije Universiteit Amsterdam, De Boelelaan 1108, 1081 HZ, Amsterdam, the Netherlands
[3] Analytical-Chemistry Group, van 't Hoff Institute for Molecular Sciences, University of Amsterdam, Science Park 904, 1098 XH Amsterdam, the Netherlands
[4] Centre for Analytical Sciences Amsterdam, Science Park 904, 1098 XH Amsterdam, the Netherlands
[5] Amsterdam School for Heritage, Memory and Material Culture, Conservation and Restoration of Cultural Heritage, University of Amsterdam, P.O. Box 94522, 1090 GN, Amsterdam, the Netherlands
[6] Luxembourg Centre for Systems Biomedicine, University of Luxembourg, 6 avenue du Swing, L-4367 Belvaux, Luxembourg
[7] KWR Water Research Institute, Groningenhaven 7, 3430 BB, Nieuwegein, the Netherlands

‡ These authors contributed equally.
* Corresponding Author: r.helmus@uva.nl

Number of Pages: 26

Numbers of Figures: 12

Number of Tables: 12

Table of Contents

S1 Materials and Methods 3
S1.1 Materials 3
Table S1 3
Table S2 4
S1.2 Adjustments for the TooCOLD box for light-induced degradation 5
S1.3 LC-HRMS methodology 5
S1.4 Analytical repeatability 6
S1.5 Feature prioritization 6
Table S3 6
S1.6 Retrieval of TP suspects 6
Table S4 7
S1.7 Post-processing of MS^2 data 7
Figure S1 8
Table S5 9
S1.8 Formula and compound annotation workflows for suspects 9
S1.9 Assignment of identification confidence levels 10
Table S6 10
S1.10 Retention time direction filtering of structure candidates for unknowns 11
S1.11 Ranking of unknown TP candidates 11
Table S7 11
Figure S2 12
Table S8 12
S1.12 Semi-quantitation of identified TPs 13
Table S9 13
Figure S3 14
S2 Results and Discussion 14
S2.1 Degradation of parent compounds 14
Figure S4 14
S2.2 Detected features and feature prioritization 15
Figure S5 15
Figure S6 15
Suspect TP screening 16
Figure S7 16
S2.3 Screening for unknown TPs 16
Figure S8 16
S2.4 Overview of identified transformation products 17
Figure S9 17
Table S10 18
Figure S10 19
Figure S11 20
S2.5 Detection of novel metoprolol+H_2O_2 TP candidates 20
Figure S12 20
S2.6 Semi-quantitative mass balances 21
Table S11 21
Table S12 22
References 24

S1 Materials and Methods

S1.1 Materials

The analytical standards used for quantitation and confirmation of transformation products were purchased from Sigma-Aldrich (Zwijndrecht, the Netherlands), TCI Europe (Zwijndrecht, Belgium) and VWR (Amsterdam, the Netherlands), see Table S1. Methanol (ULC/MS-CC/SFS grade), 2-propanol (ULC-MS grade), and glacial acetic acid (ULC/MS-CC/SFS grade) were supplied by Biosolve (Valkenswaard, the Netherlands), H_2O_2 was supplied by Sigma-Aldrich and ultrapure water was obtained from a Milli-Q Reference device (Merck Chemicals B.V., Amsterdam, the Netherlands). Stocks of analytical standards were prepared by dissolving the solid compound in methanol, and diluted with ultrapure water prior to analysis.

Table S1. Additional information on parent compounds and compounds used for identification confirmation of TPs.

Chemical	Supplier (purity)	SMILES	RT[a]	Quantifier m/z
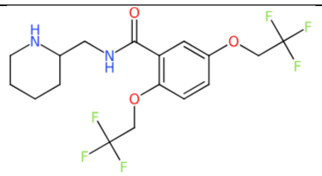 Flecainide ($C_{17}H_{20}F_6N_2O_3$)	Sigma-Aldrich (≥98%)	C1CCNC(C1)CNC(=O)C2=C(C=CC(=C2)OCC(F)(F)F)OCC(F)(F)F	8.2	417.1509[b]
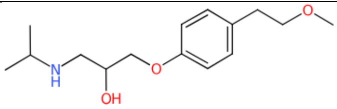 Metoprolol ($C_{15}H_{25}NO_3$)	Sigma-Aldrich (≥99%)	CC(C)NCC(COC1=CC=C(C=C1)CCOC)O	6.7	270.1965[b]
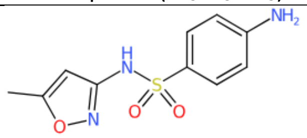 Sulfamethoxazole ($C_{10}H_{11}N_3O_3S$)	Sigma-Aldrich (≥98%)	CC1=CC(=NO1)NS(=O)(=O)C2=CC=C(C=C2)N	7.5	191.1079[b]
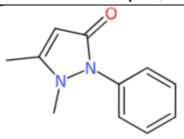 Phenazone ($C_{11}H_{12}N_2O$)	Sigma-Aldrich (≥97.5%)	CC1=CC(=O)N(N1C)C2=CC=CC=C2	7.4	256.0573[b]
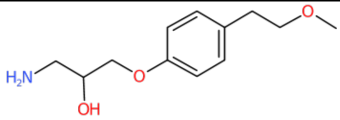 1-amino-3-[4-(2-methoxyethyl)phenoxy]propan-2-ol ($C_{12}H_{19}NO_3$)	Sigma-Aldrich (95%)	NCC(COC1=CC=C(C=C1)CCOC)O	6.1	226.1438
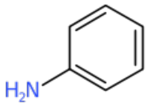 Aniline (C_6H_7N)	Sigma-Aldrich (≥99.5%)	Nc1ccccc1	3.9	94.0651
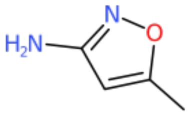 3-amino-5-methylisoxazole ($C_4H_6N_2O$)	TCI Europe (≥97%)	Cc1cc(N)no1	4.7	99.0553

Chemical	Supplier (purity)	SMILES	RT[a]	Quantifier m/z
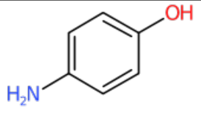 p-aminophenol (C_6H_7NO)	TCI Europe (>98%)	C1(=CC=C(C=C1)N)O	1.9	110.0600
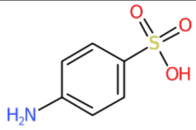 Sulfanilic acid ($C_6H_7NO_3S$)	VWR (≥99-102%)	Nc1ccc(cc1)S(O)(=O)=O	2.8	174.0219
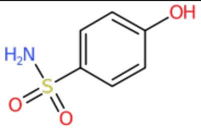 4-hydroxybenzenesulfonamide ($C_6H_7NO_3S$)	Sigma-Aldrich (unspecified)	NS(=O)(=O)c1ccc(O)cc1	4.8	174.0219
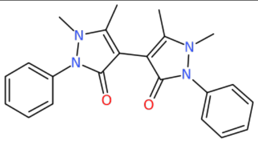 Forbisen ($C_{22}H_{22}N_4O_2$)	Sigma-Aldrich (unspecified)	CC1=C(C(=O)N(N1C)C2=CC=CC=C2)C3 =C(N(N(C3=O)C4=CC=CC=C4)C)C	9.4	375.1816
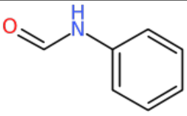 Formanilide (C_7H_7NO)	VWR (≥99%)	C1=CC=C(C=C1)NC=O	7.9	122.0600
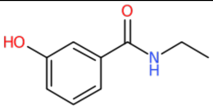 n-ethyl-3-hydroxybenzamide ($C_9H_{11}NO_2$)	Sigma-Aldrich (95%)	CCNC(=O)C1=CC(=CC=C1)O	7.2	166.0863
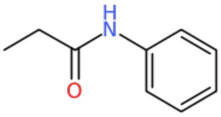 n-phenylpropanamide ($C_9H_{11}NO$)	Sigma-Aldrich (98%)	CCC(=O)NC1=CC=CC=C1	8.9	150.0913

[a]: LC retention time (minutes); [b]: The *m/z* of the M+2 isotope.

Table S2. Chemical parameters of the water used as source for the infiltration site where the water sample of this study was taken.

Chemical parameter	Value
pH	8.49 (at 11.7°C)
Total organic carbon	6.39 mg/L
Dissolved organic carbon	6.02 mg/L
Chloride	83 mg/L
Bromide	0.19 mg/L
Conductivity	61.8 mS/m (at 10.8 °C)

S1.2 Adjustments for the TooCOLD box for light-induced degradation

Several method development steps were undertaken to address sample carryover and improve sample recovery. During the initial testing of the setup, significant carryover (4.6-16.3%) was observed for all analytes and additional cleaning steps were therefore incorporated. The cleaning solvent was changed from 75% to 100% methanol, and a cleaning step with 10% 2-propanol solution was added. These supplementary cleaning procedures effectively minimized any residual traces of previous samples (0.3-0.8 %). The degradation setup was equipped with a larger sample loop (from 20 µL to 200 µL), which allowed LC-HRMS analysis of the full sample volume from the reaction cell (60 µL) and consequently improve detection limits.

The irradiation source was changed to a Short-Arc Mercury lamp (USH-102D, Ushio, Tokyo, Japan) with a characteristic UV wavelength of 254 nm. Several adjustments to the setup were made to accommodate this change, since the shorter wavelengths of UV exhibit significant and complex variations in bending angles when passing through a refractive medium, such as a lens element[1]. To ensure precise light focusing and maximize transmission, the position of the lens in the lamp-house was optimized, the inner diameter of the optical fiber was increased to 600 µm (SMA905, 0.50 NA, Thorlabs, Newton, NJ, United States) and mounted UVFS plano-convex lenses (LA4052-ML, f=35 mm, Thorlabs) were added to the filter wheel to couple the UV light from the source into the liquid-core waveguide. With these optimizations the UV transmission at 254 nm from the lamp towards the inlet of the liquid core waveguide was increased from 4% (0.8 mW) to 17% (3.5 mW), as measured with a power and energy meter (Nova II, Ophir, the Netherlands).

To conduct degradation experiments with H_2O_2, a fresh dilution of this reactant was prepared daily, and automatically added to samples (final concentration 10 mg/L) and mixed by the sample introduction system 30s prior to the start of the experiment.

S1.3 LC-HRMS methodology

Chromatographic separation was performed with a ZORBAX Eclipse Plus C18 column (Agilent, 2.1 x 150mm, Zorbax C18 packing, particle size 3.5 µm) equipped with a ZORBAX security guard column (Agilent, 2.1 x 50mm, Zorbax C18 packing, particle size 1.8 µm). The guard column contained smaller particles than the analytical column to add an additional “filtering” step and extend the lifetime of the latter. The guard column was periodically exchanged as a precautionary measure to avoid and minimize the potential interaction between H_2O_2 and the column materials. The mobile phase consisted of ultrapure water acidified with 0.05% acetic acid (A) and methanol (B). The eluent gradient started at 5% B and increased to 100% B using a linear incline until 13 min, held at 100% B for 1 min and then returned to the initial conditions. The flow rate was set to 300 µL/min and the column was kept at room temperature. The whole volume of the TooCOLD box sample loop was injected (200 µL).

The typical resolving power of the HRMS was 30,000–60,000 FWHM. The HRMS was mass calibrated with a sodium acetate in ultrapure water/methanol (1:1) mixture to ensure good mass accuracy. The instrument was calibrated at the start of each analysis batch by directly infusing a 2 mM calibrant solution. In addition, 20 µl of a 50 µM calibrant solution was injected automatically prior to each analysis and used for internal mass re-calibration. After calibrant injection, the LC flow was diverted to waste from 1.0-2.0 min to protect the MS ionization source from e.g. unretained salts in the sample matrix. Acquisition of spectra were within a range of 20 to 1000 m/z at 5 Hz rate, and MS^2 data was recorded with data-dependent mode (‘autoMS/MS’). The ionization source settings were set as follows: End plate offset 500 V, Capillary voltage 3500 V, Nebulizer 1 Bar, Dry Gas flow 8.0 L/min and Dry Gas temperature 200 °C.

S1.4 Analytical repeatability

To assess the repeatability and accuracy of the measurements, standards of 25 and 125 µg/L were systematically analyzed in all experimental batches. Freshly prepared standards were employed before each measurement to ensure consistency. The measurement precision was evaluated by performing five replicates, from which the standard deviations were determined. The resulting standard errors were found to be 4.9% for flecainide, 3.2% for metoprolol, 1.1% for phenazone, and 4.0% for sulfamethoxazole. These values reflect the degree of variability associated with the measurements and serve as indicators of the reliability of the analytical method.

S1.5 Feature prioritization

The features were prioritized in four steps, see Table S3. Firstly, common rule-based filters of patRoon, such as blank subtraction and required presence in all replicates, were applied to improve the overall quality of the feature dataset. Secondly, features of interest were selected by parent/TP regression, as described in the main text. A tolerant minimum correlation coefficient ($R^2 \geq 0.5$) was chosen to account for small linear deviations due to features with intensities (partially) outside the linear MS detection range or transformations with higher order (n>1) dependence on the initial parent concentration. Thirdly, features were annotated with adduct and isotope information with the 'componentization' functionality of patRoon[2,3] via cliqueMS[4], which employs a similarity network algorithm to group features with similar LC elution profiles and subsequently uses their *m/z* differences to assign and score putative adducts and isotopes. Next, features were subsequently removed if without 'preferential adducts/isotopes' (defined in Table S3). Fourthly, the chromatography of features was manually assessed and features with poor peak shape (e.g. false positives due to noise) were removed accordingly.

Table S3. Applied feature prioritization constraints.

Step	Approach	Feature constraint
1	Rule-based filters	Elute after system dead volume (60 seconds)
		Peak intensity ≥1000 detector counts
		Peak intensity ≥5x higher compared to solvent blanks and 0h control samples
		Present in all replicates with intensity variation of ≤75% RSD
2	Parent/TP regression	Positive slope
		Significant linearity (p<0.05)
		Detected in at least the two highest test concentrations of mix samples
		Correlation coefficient (R^2) ≥0.5
3	Adduct & Isotopes	Is annotated with 'preferential' adduct/isotope (M+H, monoisotope) *OR* the most intense if no preferential adduct/isotope was detected *OR* without any annotations
4	Peak verification	Manual visual inspection peak shapes with the 'checkFeatures()' peak inspection tool of patRoon

S1.6 Retrieval of TP suspects

Suspect TPs with structural information were predicted with the Chemical Transformation Simulator[5] (CTS) and BioTransformer[6], using the environmental (BTE) or "allHuman" (BTH) reaction libraries. In addition, TPs were collected automatically from the PubChem library[7–9] (PC) and by manual literature search (LIT; see Table S13). All TPs were combined with the consensus functionality of patRoon and can be obtained from[10].

A 'metabolic logic' approach[11] was used to generate formula TP suspects by applying a set of rules with elemental transformations (see Table S4). The default rules provided in patRoon (based on Schollée *et al.*[11]) were taken verbatim, and amended the addition of H_2O_2 as a potential transformation in the experiments where hydrogen peroxide was present. The 'genFormulaTPLibrary()' function of patRoon was then used to automatically generate a database with calculated TPs from the aforementioned transformation rules. The database was then amended with TPs from literature search, and cleared of candidates also present as structure suspect and is available from[10].

The retrieval of TPs was repeated for four generations for CTS, BTE and PC, while two generations were considered for BTH to avoid excessive numbers of suspect TPs. All TPs of the reported pathways were considered from LIT. For metabolic logic, which inherently lacks chemical specificity, only two generations were considered to avoid excessive predictions of suspects that are likely chemically inconceivable.

Table S4. Elemental transformation rules applied to calculate formula suspects. The rules (except dihydroxylation) were taken verbatim from the default of patRoon (based on Schollée et al.[11]).

**Transformation**	**Elemental change**	**Transformation**	**Elemental change**
Hydroxylation	+O	Acetylation	$+C_2H_2O$
Demethylation	$-CH_2$	Deacetylation	$-C_2H_2O$
Deethylation	$-C_2H_4$	Glucoronidation	$+C_6H_8O_6$
Dehydrogenation	$-H_2$	Deglucoronidation	$-C_6H_8O_6$
Hydrogenation	$+H_2$	Sulfonation	$+SO_3$
Dehydration	$-H_2O$	Desulfonation	$-SO_3$
Chlorine reduction	+H -Cl	Dihydroxylation	$+H_2O_2$

S1.7 Post-processing of MS^2 data

The MS^2 data was first filtered with the 'filter()' function of patRoon to remove mass peaks with <5% intensity and outside the top 25 most intense mass peaks. Next, an algorithm was developed for the detection and removal of common MS^2 background peaks. For each blank sample, all MS^2 spectra were loaded using mzR[12], post-processed to remove peaks with low intensity (<10000 detector counts) or outside the top twenty-five most intense, and finally averaged by hierarchical clustering (using fastcluster[13]). The averaging step was repeated to combine the averaged MS^2 spectra of all blank samples. Mass peaks present in ≥80% of the blank samples and ≥10% of all MS^2 spectra were considered as common background and removed from experimental spectra accordingly.

Further MS^2 cleanup was achieved by the 'annotatedBy' filter of patRoon, which employs a noise removal approach similar to Stravs *et al.*[14], extended to any matching candidate formula. Formula annotations were performed with GenForm[15] according to the main workflow (see Section S1.8) with the addition of P (i.e. CHNOPSF) to widen the candidate search range. In brief, GenForm first employs a mathematical approach to calculate all possible elemental combinations that fit the *m/z* value of the feature (5 ppm tolerance). Formula candidates are subsequently removed that (1) do not fulfill common valency rules (e.g. with disconnected bonds), (2) do not contain at least one carbon atom and heteroatom and (3) are outside the top 50 highest ranked formulae. Next, GenForm performs a similar approach to calculate candidate formulae for each MS^2 peak of the feature, and candidates that do not fulfill the aforementioned valency rules and are not radicals are discarded. This resulted in MS^2 annotations for a wide range of formula candidates assigned to each feature (10-15 on average) without taking any structural information into account, and improved annotation scores for all candidates (demonstrated in Figure S1 and Table S5). The dataset was then enlarged with the MS^2 annotations from the compound annotation candidates obtained throughout the main workflow. Any

experimental MS^2 peaks that were left fully unexplained by any of the candidate annotations were considered noise and removed accordingly.

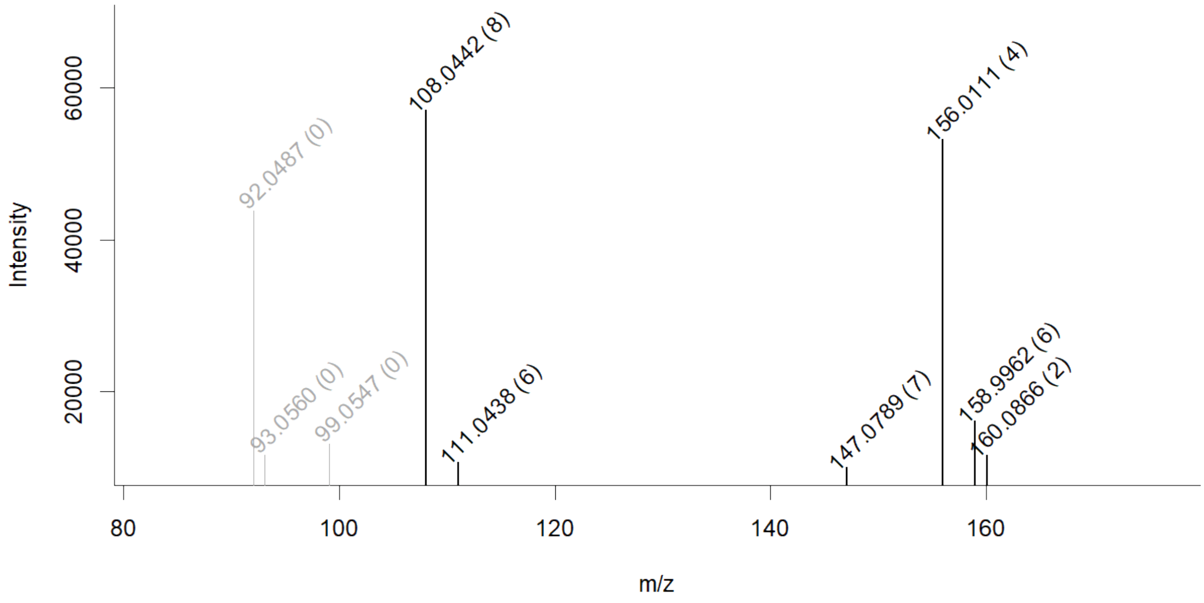

Figure S1. Example of MS^2 spectrum cleanup for sulfamethoxazole with the formula annotation cleanup procedure described above. The peak annotations are m/z (total number of formula MS^2 annotations, see Table S5). The light grey peaks with zero annotations are removed. The unfragmented ion was omitted for clarity.

Table S5.Example of MS^2 spectrum cleanup for sulfamethoxazole (experimental m/z 254.0589, data obtained from mixture experiment with UV, H_2O_2 and NOM at 25 µg/L) with the formula annotation cleanup procedure described above. Annotations for the unfragmented ion were omitted for clarity.

**Candidate**[a]	**ΔMass (ppm)**	**MS^2 annotations**[b]	**$ann_{formula}$** [c]	
			**Before**	**After**
<u>$C_{10}H_{12}N_3O_3S$</u>	1.3	108.0442 (C_6H_6NO), 111.0438 ($C_6H_7O_2$), 147.0789 ($C_8H_9N_3$), 156.0111 ($C_6H_6NO_2S$), 160.0866 ($C_9H_{10}N_3$)	0.85	0.98
$C_3H_9FN_9O_2S$	-4.8	108.0442 (CH_5FN_4O), 111.0438 ($CH_6FN_3O_2$), 147.0789 ($C_3H_8FN_6$), 156.0111 ($CH_5FN_4O_2S$), 158.9962 (CHN_7OS)	0.86	0.99
$C_7H_{10}F_2N_3O_5$	-3.0	108.0442 (C_6H_6NO), 111.0438 ($CH_6FN_3O_2$), 158.9962 ($C_5H_2FNO_4$)	0.64	0.74
$C_8H_{14}FNO_5P$	-1.0	108.0442 (C_6H_6NO), 111.0438 ($C_6H_7O_2$), 158.9962 ($C_5H_2FNO_4$)	0.64	0.74
$C_9H_{10}FN_5OP$	4.3	108.0442 (CH_5FN_4O), 147.0789 ($C_8H_9N_3$), 160.0866 ($C_9H_{10}N_3$)	0.63	0.72
$C_3H_{11}FN_9P_2$	0.2	147.0789 ($C_3H_8FN_6$)	0.10	0.12
$C_4H_{13}N_7O_2PS$	-2.8	147.0789 ($C_4H_{12}N_4P$), 156.0111 ($C_2H_9N_2O_2PS$), 158.9962 (CHN_7O_S)	0.60	0.69
$C_5H_{12}F_4N_3O_2S$	-3.9	111.0438 ($CH_6FN_3O_2$), 158.9962 ($C_3H_4F_3NOS$)	0.20	0.24
$C_{12}H_9N_5P$	-0.2	147.0789 ($C_8H_9N_3$)	0.10	0.12
$C_8H_6F_2N_7O$	2.3	108.0442 (CH_5FN_4O), 156.0111 ($C_4F_2N_5$)	0.83	0.95
$C_2H_7F_2N_{11}P$	-1.8	n.a.	n.a.	n.a.
$C_4H_{10}F_5N_5P$	-0.8	n.a.	n.a.	n.a.
$C_2H_8N_9O_6$	0.6	n.a.	n.a.	n.a.
$C_4H_{11}F_3N_3O_6$	1.5	n.a.	n.a.	n.a.
$C_{10}H_9F_5NO$	3.2	108.0442 (C_6H_6NO)	0.60	0.70
$C_{11}H_5FN_7$	-2.2	n.a.	n.a.	n.a.
$C_{13}H_8F_4N$	-1.3	n.a.	n.a.	n.a.
$C_3H_6F_6N_7$	-2.9	n.a.	n.a.	n.a.
$C_8H_{14}F_2N_3S_2$	0.4	n.a.	n.a.	n.a.
$C_{18}H_8NO$	3.9	108.0442 (C_6H_6NO)	0.60	0.70
$C_4H_{15}N_7P_3$	2.3	147.0789 ($C_4H_{12}N_4P$)	0.10	0.12
$C_3H_{12}N_9OS_2$	4.0	158.9962 (CHN_7OS)	0.17	0.20
$CH_3F_3N_{13}$	-3.8	n.a.	n.a.	n.a.
$C_9H_{18}FNPS_2$	2.5	n.a.	n.a.	n.a.
$C_5H_{14}F_4N_3P_2$	1.2	n.a.	n.a.	n.a.

[a]: of M+H ion; [b]: MS^2 peak *m/z* (calculated formula); [c]: formula annotation similarity (see Section S1.11) before and after removal of fully unexplained MS^2 peaks; *italic*: present in PubChem [16,17] (as of January 2025); <u>underlined</u>: correct candidate. n.a.: not available

S1.8 Formula and compound annotation workflows for suspects

Formula annotation was performed with GenForm[15], and candidates were only considered if with elements also present in the parent formula (i.e. CHNO and additionally F/S for flecainide/sulfamethoxazole, see SI-1-1). Compound annotation was performed with MetFrag[18], using a compound database automatically generated by patRoon with all suspect TPs. This ensured all suspects could be assessed with *in silico* annotation, as their structure may be missing in commonly used compound databases[3] such as PubChem[17].

S1.9 Assignment of identification confidence levels

The identification confidence level[19] for each TP candidate was determined according to the rules from Table S6. These were largely derived from the default ruleset of patRoon[3], and included several sub-levels to highlight differences in annotation confidence among candidates of the same level. The assignment of identification levels was largely automated (see Table S6), using the 'annotateSuspects()' function of patRoon for suspect candidates, and an extension of this algorithm for the assignment of unknowns (code available in [10]). The reference standards which were used for the level 1 and 3a assignments were bought based on commercial availability and price and are summarized in section S1.

Table S6. Identification confidence scheme applied in this study. All match value constraints range from zero (no match) to one (full match). Bold levels deviate from the default rules supplied by patRoon.

**Category**	**Identification level**	**Constraints**	**Determination**
Confirmed structure	1	Retention time match with reference standard (±0.1 min)	Target analysis reference standard
		High cosine MS^2 similarity with reference standard (≥0.90)	patRoon[a]
		Unequivocal isomer assignment	Manual
Probable structure	2a	High MetFrag 'OfflineIndividualMoNAScore' score[b] (≥0.9)	patRoon[c]
		Top ranked in compound annotations	patRoon[c]
		Unequivocal isomer assignment	Manual
Tentative structure	3a	As level 1, but without unequivocal isomer assignment	See level 1
	3b	High *in silico* MS^2 compound annotation match (≥0.90)	patRoon[c]
		Top ranked in compound annotations	patRoon[c]
	3c	Fair MetFrag 'OfflineIndividualMoNAScore' score[b] (≥0.7)	patRoon[c]
	3d	Good *in silico* MS^2 compound annotation match (≥0.7)	patRoon[c]
Unequivocal formula	4a	Good *in silico* MS^2 formula annotation match (≥0.7)	patRoon[c]
		Fair isotopic pattern match (≥0.5)	patRoon[c]
		Isotopic match at least ≥0.2 compared to other candidates	patRoon[c]
		Top ranked in formula annotations	patRoon[c]
Tentative formula	4b	High *in silico* MS^2 formula annotation match (≥0.9)	patRoon[c]
		Fair isotopic pattern match (≥0.5)	patRoon[c]
		Top ranked in formula annotations	patRoon[c]
	4c	High isotopic pattern match (≥0.9)	patRoon[c]
		Isotopic match at least ≥0.2 compared to other candidates	patRoonc[3]
		Top ranked in formula annotations	patRoon[c]
Exact mass	5	None	patRoon[c]

[a]: Spectral similarities calculated with patRoon; [b]: spectral match in a curated subset of MoNA MassBank[20,21]
[c]: Using the rule based identification confidence level estimation approach of patRoon.

S1.10 Retention time direction filtering of structure candidates for unknowns

The expected LC retention order of a candidate compared to its parent was used to discard unlikely candidates, using an algorithm that was based on the 'retDirMatch' component filter of patRoon[3]. In this study LC separation was achieved with reversed phase, hence, polar compounds generally elute prior to less polar molecules. The difference in polarity between the parent and candidate was assessed by comparing their log P values (calculated as 'XLogP' with rcdk[22]). Differences ≤1 unit were considered acceptable to allow a tolerance regarding imperfect log P predictions and LC elution behavior not solely explained by polarity. Otherwise, the candidate was assumed to elute prior to its parent for smaller log P values and *vice versa*. The observed retention order was similarly determined by comparing the retention times of the parent and candidate, and a difference of ≤30 s was considered acceptable. The candidate was then removed if (1) the log P values and retention times differed (beyond the indicated tolerances) from the parent *and* (2) there was a mismatch between expected and observed elution order.

S1.11 Ranking of unknown TP candidates

The definitions of the metrics used to rank TP candidates for unknowns is summarized in Table S7.

Table S7. Definition of the metrics used to rank TP annotation candidates for unknowns.

	**Metric**	**Calculation**
Compounds	$fit_{compound}$	Overlap coefficient calculated with fmcsR[23].
	$sim_{suspects}$	Tanimoto distance[24] of the parent/TP extended molecular fingerprints (calculated with rcdk[22]).
	$ann_{compound}$	Cosine similarity[25] of the intensity normalized MS^2 spectrum with only annotated peaks by MetFrag[18] and the full MS^2 spectrum (derived from patRoon).
	TP $score_{compound}$	$\max\{fit_{compound}, sim_{suspects}\} + ann_{compound}$
Formulas	$fit_{formula}$	Common element count divided by total element count. The maximum is taken for parent/TP and TP/parent fits.
	$ann_{formula}$	Cosine similarity[25] of the intensity normalized MS^2 spectrum with only annotated peaks by GenForm[15] and the full MS^2 spectrum (derived from patRoon).
	TP $score_{formula}$	$fit_{formula} + ann_{formula}$

The suitability of $fit_{compoun}$d, $sim_{suspsects}$ and $fit_{formula}$ was evaluated with the structure suspect data. Since the annotation similarity metrics ($ann_{compound}$ and $ann_{formula}$) were applied previously[26–28] they were not evaluated further here. The $fit_{compound}$ and $fit_{formula}$ metrics were evaluated by comparing fits for each suspect with its parent (true positives) and with any of the other parents (true negatives). Similarly, $sim_{suspects}$ was evaluated by comparing similarities calculated between suspects of the same parent (true positives) with similarities between suspects of different parents (true negatives). One suspect TP (ammonium) resulted in calculation errors, and was not considered in this evaluation. True positives for all metrics were high (median 0.8-1.0), and significantly higher than true negatives for $fit_{compound}$ and $sim_{suspects}$ (see Figure S2). However, true negatives for $fit_{formula}$ differed little (median 0.9), which is likely attributed to inherently fewer characteristics of formulas compared to structures and similar elements of the evaluated parents (i.e. primarily CHNO, see SI-1.1). Nevertheless, overlap between true positives and negatives was little, which suggest that $fit_{formula}$ can still aid in prioritization. The results of this evaluation were subsequently used to derive thresholds for each metric, see vertical bars in Figure S2.

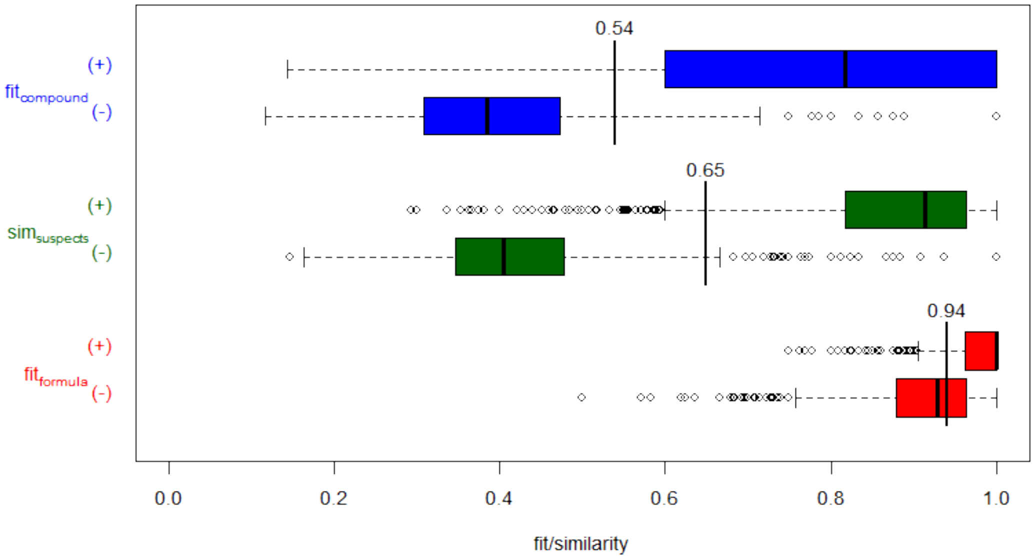

Figure S2. Evaluation results for TP ranking metrics for unknowns, tested with structure suspects data with true positives (+) and true negatives (-). The whiskers represent 1.5 times the interquartile range. The vertical black bars represent selected thresholds. For $fit_{compound}$ and $sims_{uspects}$, the thresholds were derived from the mean of the first quartile (+) and third quartile (-). The threshold for $fit_{formula}$ was derived from the mean of the first quartile (+) and median (-), and therefore less strict to accommodate the overlap between (+) and (-).

The final candidates obtained by the screening for unknown workflows were manually evaluated and eliminated when appropriate (see Table S8).

Table S8. Criteria to eliminate implausible TP candidates from the screening for unknown workflows.

Elimination criterium	Examples
Unsuitable for LC-HRMS analysis	$C_{17}H_{20}F_6N_2O_3$ → $\mathbf{C_3H_3F_5}$ (Δ-$C_{14}H_{17}F_1N_2O_3$)
	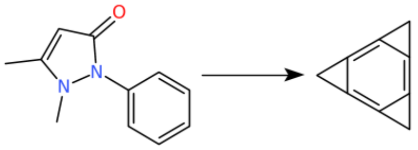
Inconceivable transformation	$C_{10}H_{11}N_3O_3S$ → $C_{12}H_{27}N_7O_4S$ (Δ$C_2\mathbf{H_{16}}N_4O$)
	$C_{15}H_{25}NO_3$ → $C_{12}H_{25}NO_3$ (Δ-$\mathbf{C_3}$)
	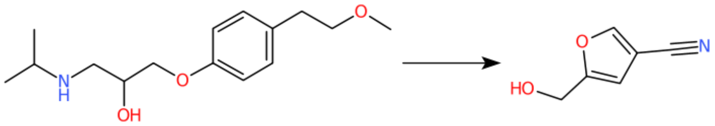
Unstable and/or kinetically unfavorable	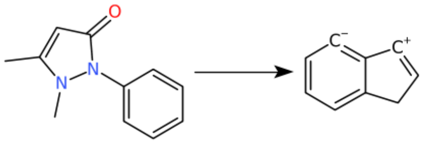
	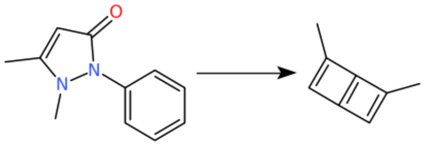

S1.12 Semi-quantitation of identified TPs

Two semi-quantitation approaches were applied in this study. The first approach was performed for TPs identified with level 1 or 3a and utilized target analysis as described in the Materials and Methods section of the main text. This is considered a semi-quantitative approach, since (1) matrix effects on MS detector response were not investigated and (2) MS detector sensitivity variability over time was not taken into account, since the standards were measured in a separate LC-MS acquisition run performed several months later.

The second semi-quantitative approach utilized MS2Quant, a machine learning based approach to predict MS ionization efficiencies[29]. The software was interfaced via patRoon, and used to obtain quantities for the remaining TPs for which no standards were available. The predictions were based on the structure model, which has a median prediction error of ~5x [29]. The MS2Quant algorithm first performs a calibration to convert instrument specific response factors to ionization efficiencies, and requires LC-MS quantitative data of multiple compounds measured at several concentrations[30]. For this purpose, dilution series of all parent and TP standards were analyzed (see Table S9). To improve the model performance, concentration points for a compound were removed if (1) no signal response, (2) MS signal overload or (3) residuals in a LC-HRMS peak area *versus* concentration regression model were >20%. Furthermore, p-aminophenol could not be included as its retention time was below the measurement range (<2 minutes), and flecainide and metoprolol were excluded as these resulted in a poor linearity of the calibration model (R^2 0.33-0.54). The performance of the final calibration model was deemed adequate, with residuals <6% and a correlation coefficient (R^2) of 0.68, which was comparable to what was described by Sepman et al.[29] (see Table S9 and Figure S3).

Table S9. Model input and output for the ionization efficiency prediction calibration procedure with MS2Quant.

Compound	Concentration points		Log RF	Log IE	Residual (%)
	Range[1] (µg/L)	n			
Sulfamethoxazole	0.29 - 19	7	13.40	2.09	2.2
Phenazone	0.07 - 4.7	6	13.79	3.42	-1.7
1-amino-3-[4-(2-methoxyethyl)phenoxy]propan-2-ol	0.2 - 10	7	13.77	2.00	5.3
3-amino-5-methylisoxazole	0.3 - 21	7	13.16	2.39	-1.2
4-hydroxybenzenesulfonamide	5.3 - 85	5	11.80	0.87	-3.8
Forbisen	0.08 - 5.2	7	14.26	3.59	0.8
Formanilide	0.33 - 21	7	13.01	2.27	-1.7
n-phenylpropanamide	0.08 - 5.2	7	13.64	2.64	1.2
n-ethyl-3-hydroxybenzamide	0.32 - 21	7	13.10	2.69	-3.3
Sulfanilic acid	2.6 - 83	6	12.31	1.43	-2.6
Aniline	0.08 - 10	8	13.20	1.49	3.8

[1]: Selection after application of the constraints from the text; RF: response factor (LC-HRMS peak area); IE: predicted ionization efficiency

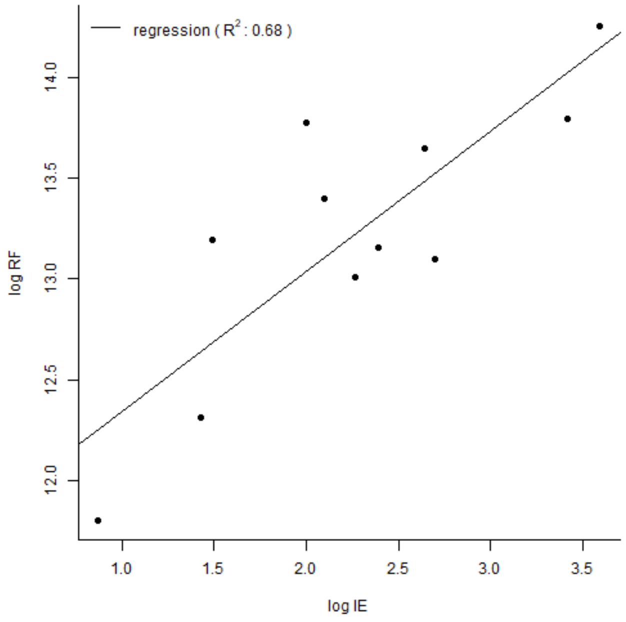

Figure S3. Observed MS response factors versus predicted ionization efficiencies from MS2Quant (both logarithmic). The regression model (black line) was used for the MS2Quant calibration procedure as described in the text.

S2 Results and Discussion

S2.1 Degradation of parent compounds

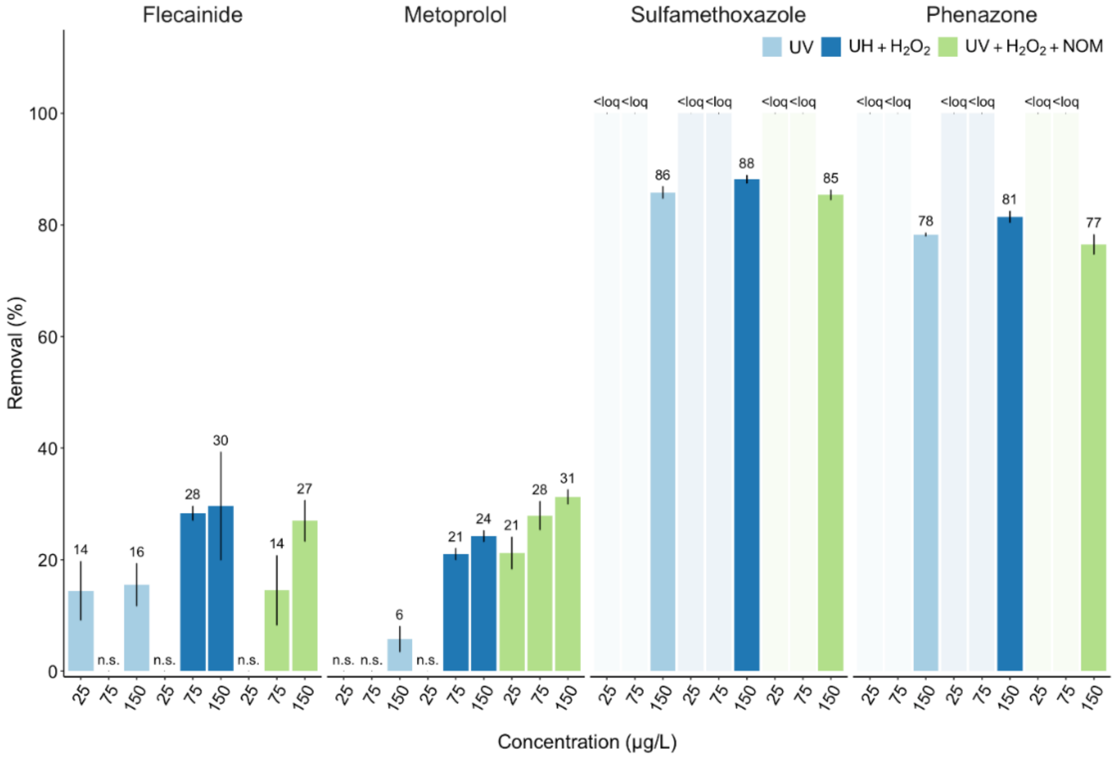

Figure S4. Percent removal of the tested parent compounds in experiments with parent mixtures at 25, 75 and 150 µg/L, which were exposed to the tested degradation conditions. Error bars represent standard errors between replicates. n.s.: no significant removal (p>0.05). <loq: measurement was below quantitation limit.

S2.2 Detected features and feature prioritization

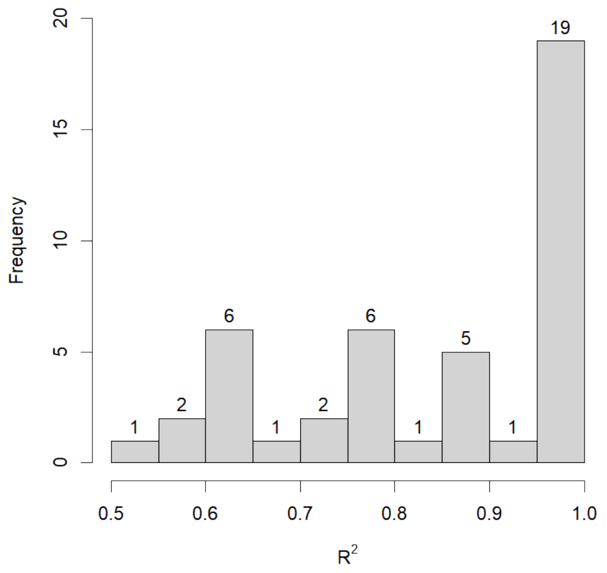

Figure S5. Frequencies for observed correlation coefficients (R^2) of the intensities of the prioritized features versus the initial parent concentration. Features present at only two initial parent concentrations were omitted as these give poor estimates of correlation coefficients.

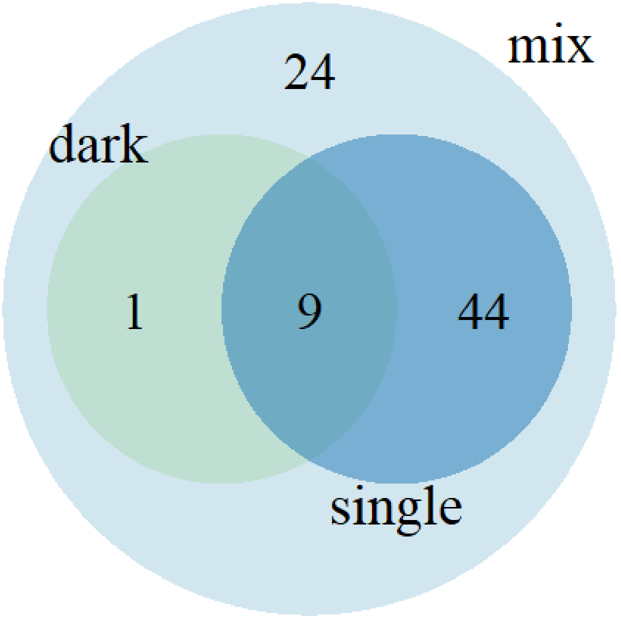

Figure S6.Distributions of prioritized features in mixture and single parent experiments and dark controls.

Suspect TP screening

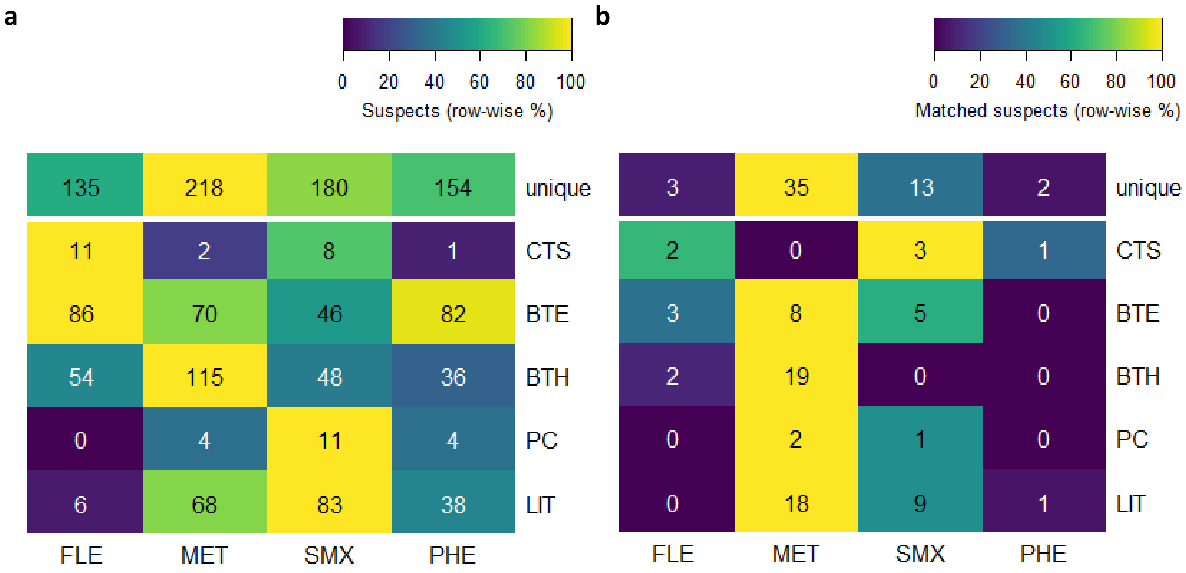

Figure S7. The number of suspects (a) and matched suspect (b) from each data source and the unique total for the parent compounds flecainide (FLE), metoprolol (MET), sulfamethoxazole (SMX) and phenazone (PHE). The data sources are the Chemical Transformation Simulator (CTS), BioTransformer with environmental or "allHuman" reaction libraries (BTE and BTH), PubChem (PC) and literature search (LIT).

S2.3 Screening for unknown TPs

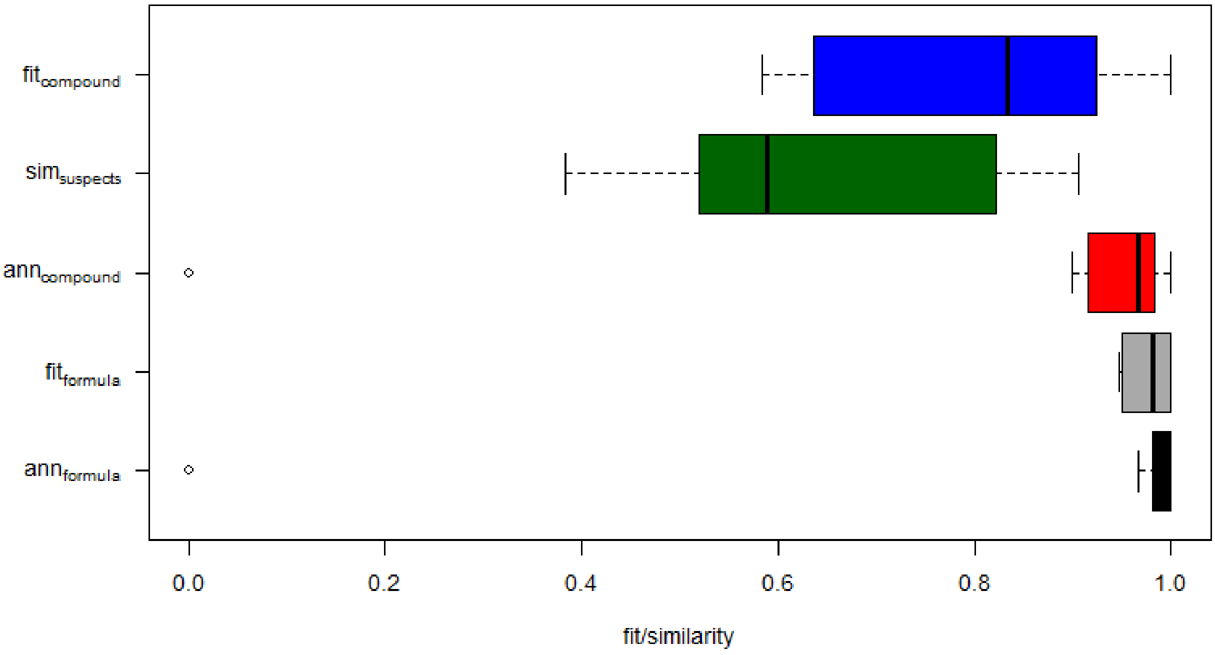

Figure S8. Observed values for the metrics used to rank candidates for unknowns. The two outliers with a zero value for $ann_{compound}$ and $ann_{formula}$ are from one feature (M122_R471_3775) with poor MS^2 data.

S2.4 Overview of identified transformation products

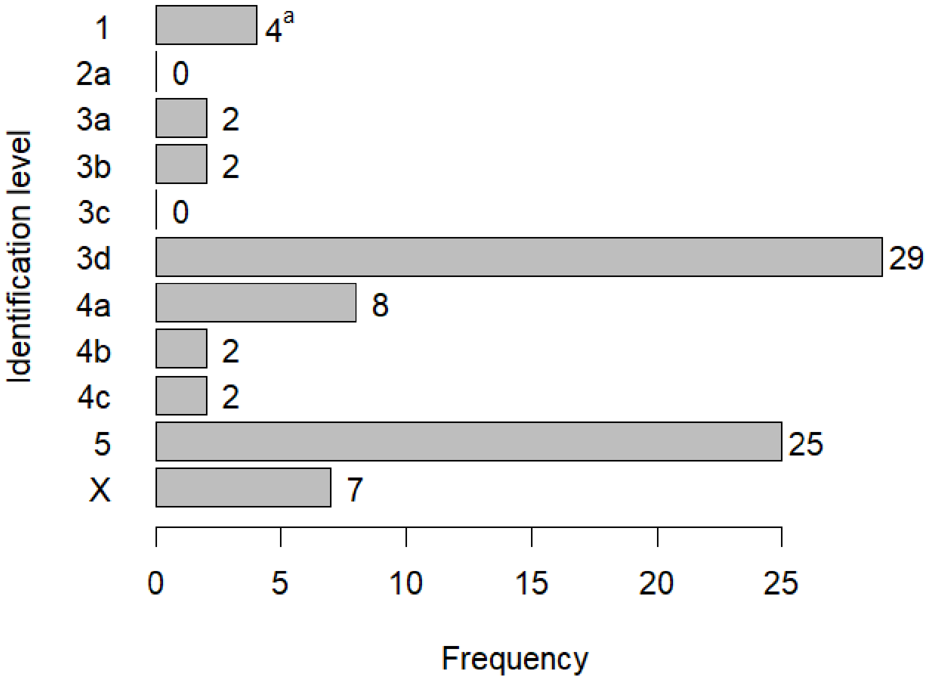

Figure S9. Distribution of identification confidence levels for all TP candidates. X: disproved by reference standard. [a]: One TP structure was formed by both sulfamethoxazole and phenazone and is therefore reported twice.

Table S10. Overview of TP candidate confirmation results with reference standards.

Parent	TP candidate	Feature	ΔRT[a]	MS^2 similarity[b]	IDL[c]
Flecainide	UnF-FLE-M166-1	M166_R473_6824	-0.6	n.a.	Disproved
Metoprolol	SuS-MET-M226-3	M226_R366_2919	0.0	0.92	3a
Sulfamethoxazole	SuS-SMX-M94-1	M94_R231_2478	0.0	0.98	1
	SuS-SMX-M99-1	M99_R289_3167	-0.1	0.99	1
		M99_R136_3553	2.5	0.99	Disproved
	SuS-SMX-M110-1[d]	M110_R177_3768	-1.1	0.73	Disproved
		M110_R126_3769	-0.2	n.a.	Disproved
	SuS-SMX-M174-4	M174_R162_5656	0.1	0.91	3a
	SuS-SMX-M174-1	M174_R162_5656	2.1	n.a.	Disproved
Phenazone	SuS-PHE-M94-1	M94_R231_2478	0.0	0.98	1
	UnS-PHE-M375-1	M375_R598_4727	-0.6	0.01	Disproved
	UnF-PHE-M122-1	M122_R471_3775	0.1	0.93[e]	1
	UnF-PHE-M150-1	M150_R528_2607	0.1	0.0	Disproved

[a]: LC retention time difference (min) between reference standard and experiments (threshold ±0.1) ; [b]: Cosine MS^2 similarity (threshold ≥0.90); [c]: identification confidence level; [d]: the LC retention time of the reference standard (1.9 min) was outside the measurement window of the experiments (≥2 min), hence, it is unknown if this TP was formed; [e]: from MS^2 data of a repeated single parent UV degradation experiment with higher initial parent concentration (1200 µg/L); n.a.: no reference and/or experimental MS^2 data available

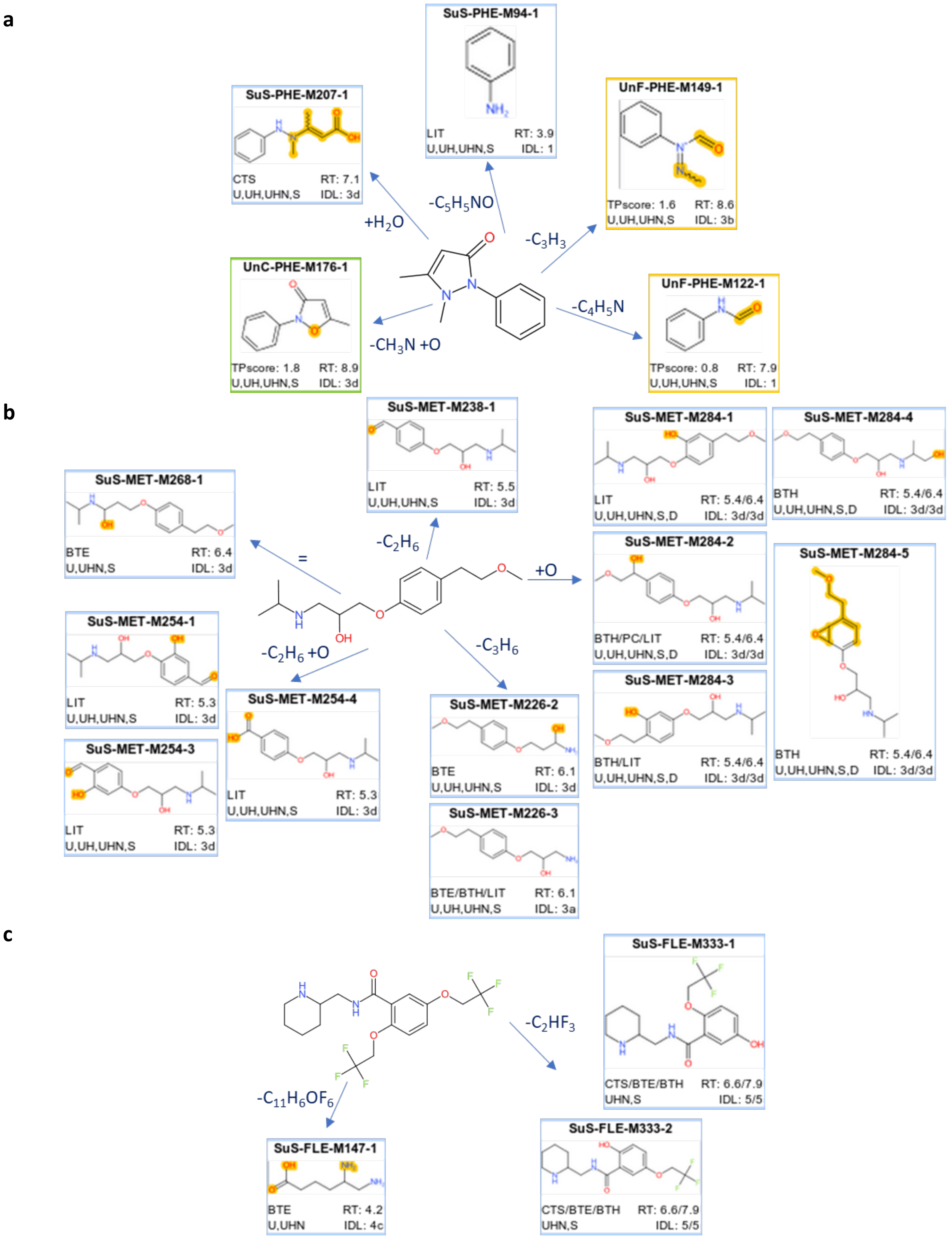

Figure S10. Overview all candidate TPs for (a) phenazone, (b) metoprolol and (c) flecainide (for a-b only candidates with identification confidence level 3 or better are shown). The box color signifies if the candidate was obtained by structure suspect screening (blue) or unknown screening with formula (yellow) or compound (green) annotation workflow. Each candidate structure is named by a unique TP identifier that refers to SI-2. The yellow shades in the TP structures represent additions or changes in atoms or bond order as compared to the parent. Box annotations: CTS, BTE, BTH, LIT: suspect from Chemical Transformation Simulator, BioTransformer with environmental or "allHuman" reaction libraries, and literature, respectively; U, UH, UHN: presence in mix experiments exposed to UV, UV and H_2O_2, UV, H_2O_2 and NOM, respectively; S or D: present in any of the single parent experiments or dark controls; RT: retention time (minutes); IDL: identification confidence level, see section S1.9. Candidates matched to multiple feature are reported with the retention times, the best identification level, and the sum of all experimental conditions for all features.

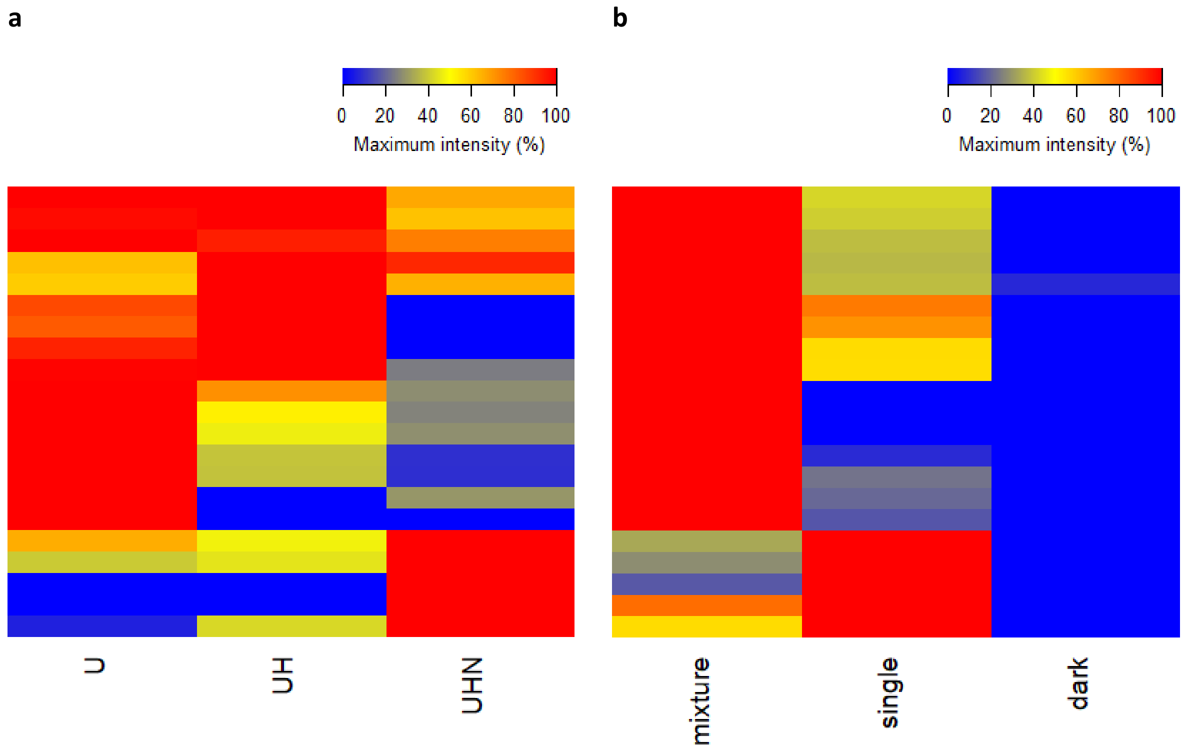

Figure S11. Feature Intensities of the candidate TPs discussed in the 'Overview of identified transformation products' from the main text for (a) mixture experiments that were degraded with UV (U), UV and H_2O_2 (UH) and UV, H_2O_2 and NOM (UHN) and (b) mixture and single parent experiments and dark controls (maximum from all experimental conditions). All data was from experiments with an initial parent concentration of 150 µg/L.

S2.5 Detection of novel metoprolol+H_2O_2 TP candidates

A novel metoprolol+H_2O_2 formula suspect that was calculated with metabolic logic was matched with two closely eluting features (peaks 2-3 in Figure S12 and features M302_R337_5079 and M302_R316_5723 in Report R1). These features were detected only in the presence of H_2O_2, detected in both single and mixed parent experiments, and the one feature with MS^2 data (M302_R337_5079) showed good formula annotation confidence (level 4a). The features eluted prior to metoprolol, which (a) suggests that these TPs are more polar, which can be expected from the addition of two hydroxyl groups, (b) excludes that the observed features can be MS adducts, and (c) indicates formation of different isomers.

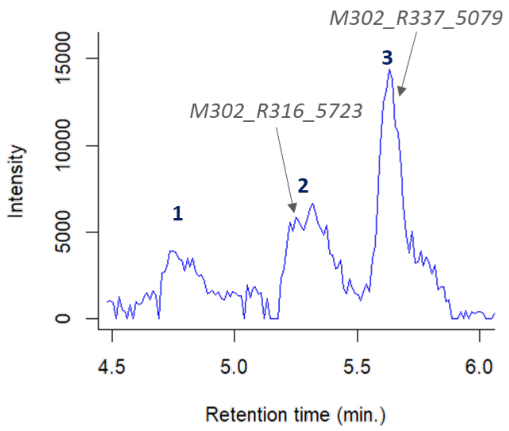

Figure S12. Extracted ion chromatogram with the m/z of [metoprolol+H_2O_2+H]+. The corresponding feature name for each peak is labelled in dark grey. The first peak was removed from the feature dataset during prioritization.

S2.6 Semi-quantitative mass balances

Table S11. Semi-quantitative molar mass balance for the TP candidates with identification confidence level 3 or better derived from semi-quantitation with standards for parent mixture and single-parent experiments.

**Parent[a]**	**Feature**	**TP candidate(s)[b]**	**Parent removal composition (%)[c]**					
			**mix$_{U}$**	**mix$_{UH}$**	**mix$_{UHN}$**	**single$_{U}$**	**single$_{UH}$**	**single$_{UHN}$**
Metoprolol	M226_R366_2919	SuS-MET-M226-3 (3a)<br>SuS-MET-M226-2 (3d)	29 (7)	5.2 (0.4)	7.4 (0.5)	4.8 (0.6)	n.a.	6.9 (0.9)
	**Total**		29 (7)	5.2 (0.4)	7.4 (0.5)	4.8 (0.6)	n.a.	6.9 (0.9)
Sulfamethoxazole	M174_R162_5656	SuS-SMX-M174-4 (3a)<br>SuS-SMX-M174-2 (3d)<br>SuS-SMX-M174-3 (3d)	5.38 (0.16)	6.3 (0.5)	n.a.	n.a.	n.a.	n.a.
	M94_R231_2478[d]	SuS-SMX-M94-1 (1)	-	-	-	2.07 (0.03)	2.4 (0.2)	3.2 (0.3)
	**Total**		5.38 (0.16)	6.3 (0.5)	n.a.	2.07 (0.03)	2.4 (0.2)	3.2 (0.3)
	M99_R289_3167[e]	SuS-SMX-M99-1 (1)	5.9 (0.5)	6.4 (0.3)	1.38 (0.06)	16.4 (0.3)	18.1 (2)	n.a.
Phenazone	M122_R471_3775	UnF-PHE-M122-1 (1)	0.34 (0.05)	0.276 (0.018)	0.149 (0.008)	0.24 (0.05)	0.23 (0.012)	0.43 (0.15)
	M94_R231_2478[d]	SuS-PHE-M94-1 (1)	n.a.	n.a.	n.a.	4.4 (1.4)	5.8 (1.1)	7.1 (0.6)
	**Total**		0.34 (0.05)	0.276 (0.018)	0.149 (0.008)	4.7 (1.4)	6.1 (1.1)	7.6 (0.6)
Sulfamethoxazole & Phenazone	M94_R231_2478[d]	SuS-SMX-M94-1 (1) &<br>SuS-PHE-M94-1 (1)	14.2 (1.2)	15 (2)	10.5 (0.7)	-	-	-
	**Total**		14.2 (1.2)	15 (2)	10.5 (0.7)	-	-	-

[a]: Flecainide is absent since no standards were available for the identified TPs. [b]: (Identification confidence level); [c]: Ratio between the molar concentration of a TP and the removal of its parent from experiments with an initial parent concentration of 150 µg/L (standard deviation). If multiple TP candidates were assigned to the same feature then the results are from the candidate with identification level 3a; [d]: Candidate was assigned to sulfamethoxazole and phenazone: mixture results are from the sum removal of both parents; [e]: This candidate was not included in totals as its structure does not overlap with other TPs and may therefore be formed simultaneously; U, UH, UHN: degradation conditions for mixture and single parent experiments, i.e. UV, UV and H_2O_2 and UV, H_2O_2 and NOM, respectively; n.a.: parent and/or TP not detected, below quantitation limit or insignificant parent removal.

Table S12. Semi-quantitative molar mass balance for the TP candidates with identification confidence level 3 or better (except flecainide) derived from semi-quantitation with MS2Quant predictions for parent mixture and single-parent experiments.

Parent	Feature	TP candidate(s)[a]	Parent removal composition (%)[b]					
			mix_U	mix_{UH}	mix_{UHN}	$single_U$	$single_{UH}$	$single_{UHN}$
Flecainide	M147_R255_4120	SuS-FLE-M147-1 (4c)	41 (9)	n.a.	4.0 (0.5)	n.a.	n.a.	n.a.
	M333_R393_6915	SuS-FLE-M333-1 (5)<br>SuS-FLE-M333-2 (5)	n.a.	n.a.	0.163 (0.012)	n.a.	n.a.	0.132 (0.003)
	M333_R474_5934	SuS-FLE-M333-1 (5)<br>SuS-FLE-M333-2 (5)	n.a.	n.a.	0.3 (0.02)	n.a.	n.a.	0.146 (0.005)
	**Total**		41 (9)	n.a.	4.4 (0.5)	n.a.	n.a.	0.278 (0.006)
Metoprolol	M238_R327_2828	SuS-MET-M238-1 (3d)	76 (18)	7.8 (0.6)	1.31 (0.08)	20 (3)	n.a.	3.2 (0.5)
	M254_R321_3770	SuS-MET-M254-4 (3d)<br>SuS-MET-M254-3 (3d)<br>SuS-MET-M254-1 (3d)	30 (4)	3.19 (0.16)	0.57 (0.02)	1.87 (0.13)	n.a.	n.a.
	M268_R384_4267	SuS-MET-M268-1 (3d)	2.6 (0.6)	n.a.	n.a.	0.9 (0.2)	n.a.	0.94 (0.13)
	M284_R326_2405	SuS-MET-M284-4 (3d)<br>SuS-MET-M284-3 (3d)<br>SuS-MET-M284-2 (3d)<br>SuS-MET-M284-5 (3d)<br>SuS-MET-M284-1 (3d)	10.2 (1.3)	4.14 (0.2)	1.79 (0.06)	2.34 (0.16)	n.a.	4.4 (0.3)
	M284_R387_3283	SuS-MET-M284-4 (3d)<br>SuS-MET-M284-3 (3d)<br>SuS-MET-M284-2 (3d)<br>SuS-MET-M284-5 (3d)<br>SuS-MET-M284-1 (3d)	3.6 (0.5)	5.7 (0.6)	8.1 (0.3)	0.64 (0.04)	n.a.	20.7 (1.2)
	**Total**		123 (19)	20.8 (0.9)	11.7 (0.3)	26 (3)	n.a.	29.2 (1.4)
Sulfamethoxazole	M142_R157_5040	SuS-SMX-M142-2 (3d)<br>SuS-SMX-M142-3 (3d)<br>SuS-SMX-M142-1 (3d)	3.43 (0.07)	3.35 (0.17)	n.a.	n.a.	n.a.	n.a.
	M254_R349_3557	SuS-SMX-M254-1 (3d)	1.95 (0.09)	2.41 (0.16)	5.27 (0.18)	46.1 (0.7)	40 (5)	44 (4)
	M272_R322_3885	SuS-SMX-M272-1 (3d)<br>SuS-SMX-M272-2 (3d)	1.17 (0.03)	2.0 (0.6)	1.82 (0.03)	10.91 (0.16)	12.2 (1.4)	7.7 (0.6)
	M140_R170_3386	UnC-SMX-M140-1 (3b)	7.6 (0.5)	5.7 (0.6)	2.76 (0.14)	5.8 (0.7)	11.1 (1.5)	24 (12)

Parent	Feature	TP candidate(s)[a]	Parent removal composition (%)[b]					
			mix_U	mix_{UH}	mix_{UHN}	$single_U$	$single_{UH}$	$single_{UHN}$
Sulfamethoxazole	M190_R159_4149	UnC-SMX-M190-1 (3d)<br>UnC-SMX-M190-3 (3d)<br>UnC-SMX-M190-2 (3d)	6.35 (0.18)	7.1 (0.5)	n.a.	2.0 (0.4)	2.35 (0.15)	n.a.
	**Total**		20.5 (0.6)	20.6 (1.0)	9.8 (0.2)	64.8 (1.1)	66 (6)	75 (12)
Phenazone	M207_R424_3387	SuS-PHE-M207-1 (3d)	7.65 (0.07)	3.4 (0.3)	2.19 (0.13)	23 (5)	15.7 (1)	18.1 (1.2)
	M149_R515_2917	UnF-PHE-M149-1 (3b)	3.18 (0.11)	1.53 (0.11)	0.9 (0.07)	1.7 (0.6)	2.02 (0.13)	2.18 (0.14)
	M176_R533_3000	UnC-PHE-M176-1 (3d)	1.78 (0.02)	1.73 (0.12)	1.15 (0.07)	11 (3)	10.2 (0.6)	11.4 (0.8)
	**Total**		12.62 (0.13)	6.7 (0.3)	4.24 (0.16)	35 (5)	27.9 (1.2)	31.6 (1.5)

[a]: (Identification confidence level); [b]: Ratio between the molar concentration of a TP and the removal of its parent from experiments with an initial parent concentration of 150 µg/L (standard deviation). Results are mean averaged for all TP candidates to the feature.; U, UH, UHN: degradation conditions for mixture and single parent experiments, i.e. UV, UV and H_2O_2 and UV, H_2O_2 and NOM, respectively; n.a.: parent and/or TP not detected, below quantitation level or insignificant parent removal.

References

(1) Paschotta, R. Ultraviolet Optics - an Encyclopedia Article. *RP Photonics Encyclopedia* **2023**. https://doi.org/10.61835/PYP.

(2) Helmus, R.; ter Laak, T. L.; van Wezel, A. P.; de Voogt, P.; Schymanski, E. L. patRoon: Open Source Software Platform for Environmental Mass Spectrometry Based Non-Target Screening. *J Cheminform* **2021**, *13* (1), 1–25. https://doi.org/10.1186/s13321-020-00477-w.

(3) Helmus, R.; van de Velde, B.; Brunner, A. M.; ter Laak, T. L.; van Wezel, A. P.; Schymanski, E. L. patRoon 2.0: Improved Non-Target Analysis Workflows Including Automated Transformation Product Screening. *J Open Source Softw* **2022**, *7* (71), 4029. https://doi.org/10.21105/joss.04029.

(4) Senan, O.; Aguilar-Mogas, A.; Navarro, M.; Capellades, J.; Noon, L.; Burks, D.; Yanes, O.; Guimerà, R.; Sales-Pardo, M. CliqueMS: A Computational Tool for Annotating in-Source Metabolite Ions from LC-MS Untargeted Metabolomics Data Based on a Coelution Similarity Network. *Bioinformatics* **2019**, *35* (20), 4089–4097. https://doi.org/10.1093/bioinformatics/btz207.

(5) Wolfe, K.; Pope, N.; Parmar, R.; Galvin, M.; Stevens, C.; Weber, E.; Flaishans, J.; Purucker, T. Chemical Transformation System: Cloud Based Cheminformatic Services to Support Integrated Environmental Modeling. In *Proceedings of the 8th International Congress on Environmental Modelling and Software*; Toulouse, France, 2016.

(6) Djoumbou-Feunang, Y.; Fiamoncini, J.; Gil-de-la-Fuente, A.; Greiner, R.; Manach, C.; Wishart, D. S. BioTransformer: A Comprehensive Computational Tool for Small Molecule Metabolism Prediction and Metabolite Identification. *J Cheminform* **2019**, *11* (1), 2. https://doi.org/10.1186/s13321-018-0324-5.

(7) Krier, J.; Singh, R. R.; Kondić, T.; Lai, A.; Diderich, P.; Zhang, J.; Thiessen, P. A.; Bolton, E. E.; Schymanski, E. L. Discovering Pesticides and Their TPs in Luxembourg Waters Using Open Cheminformatics Approaches. *Environ Int* **2022**, *158*, 106885. https://doi.org/10.1016/J.ENVINT.2021.106885.

(8) Schymanski, E. L.; Kondić, T.; Neumann, S.; Thiessen, P. A.; Zhang, J.; Bolton, E. E. Empowering Large Chemical Knowledge Bases for Exposomics: PubChemLite Meets MetFrag. *J Cheminform* **2021**, *13* (1), 1–15. https://doi.org/10.1186/S13321-021-00489-0.

(9) Schymanski, E.; Bolton, E.; Cheng, T.; Thiessen, P.; Zhang, J. (Jeff); Helmus, R.; Blanke, G. Transformations in PubChem - Full Dataset. Zenodo December 2023. https://doi.org/10.5281/zenodo.10377222.

(10) Helmus, R.; Bagdonaite, I.; de Voogt, P.; van Bommel, M.; Schymanski, E. L.; van Wezel, A.; ter Laak, T. Code Accompanying the Manuscript "Comprehensive Mass Spectrometry to Systematically Elucidate Transformation Processes of Organic Micropollutants: A Case Study on photodegradation of Four Pharmaceuticals." Zenodo January 2025. https://doi.org/10.5281/zenodo.14671663.

(11) Schollée, J. E.; Schymanski, E. L.; Avak, S. E.; Loos, M.; Hollender, J. Prioritizing Unknown Transformation Products from Biologically-Treated Wastewater Using High-

Resolution Mass Spectrometry, Multivariate Statistics, and Metabolic Logic. *Anal Chem* **2015**, *87* (24), 12121–12129. https://doi.org/10.1021/acs.analchem.5b02905.

(12) Fischer, B.; Neumann, S.; Gatto, L.; Kou, Q.; Rainer, J. MzR: Parser for NetCDF, MzXML, MzData and MzML and MzIdentML Files (Mass Spectrometry Data). 2020. https://doi.org/10.18129/B9.bioc.mzR.

(13) Müllner, D. Fastcluster: Fast Hierarchical, Agglomerative Clustering Routines for R and Python. *J Stat Softw* **2013**, *53* (9), 1–18. https://doi.org/10.18637/jss.v053.i09.

(14) Stravs, M. A.; Schymanski, E. L.; Singer, H. P.; Hollender, J. Automatic Recalibration and Processing of Tandem Mass Spectra Using Formula Annotation. *Journal of Mass Spectrometry* **2013**, *48* (1), 89–99. https://doi.org/10.1002/JMS.3131.

(15) Meringer, M.; Reinker, S.; Zhang, J.; Muller, A. MS/MS Data Improves Automated Determination of Molecular Formulas by Mass Spectrometry. *MATCH Commun. Math. Comput. Chem* **2011**, 259–290.

(16) *PubChem Home Page*. https://pubchem.ncbi.nlm.nih.gov/ (accessed 2024-03-25).

(17) Kim, S.; Chen, J.; Cheng, T.; Gindulyte, A.; He, J.; He, S.; Li, Q.; Shoemaker, B. A.; Thiessen, P. A.; Yu, B.; Zaslavsky, L.; Zhang, J.; Bolton, E. E. PubChem 2023 Update. *Nucleic Acids Res* **2023**, *51* (D1), D1373–D1380. https://doi.org/10.1093/NAR/GKAC956.

(18) Ruttkies, C.; Schymanski, E. L.; Wolf, S.; Hollender, J.; Neumann, S. MetFrag Relaunched: Incorporating Strategies beyond in Silico Fragmentation. *J Cheminform* **2016**, *8* (1), 3. https://doi.org/10.1186/s13321-016-0115-9.

(19) Schymanski, E. L.; Jeon, J.; Gulde, R.; Fenner, K.; Ruff, M.; Singer, H. P.; Hollender, J. Identifying Small Molecules via High Resolution Mass Spectrometry: Communicating Confidence. *Environ Sci Technol* **2014**, *48* (4), 2097–2098. https://doi.org/10.1021/es5002105.

(20) MassBank of North America. *MassBank of North America*. https://mona.fiehnlab.ucdavis.edu/ (accessed 2024-03-25).

(21) *MetFragRelaunched/MetFragLib/src/main/resources/MoNA-export-LC-MS.mb at master · ipb-halle/MetFragRelaunched*. https://github.com/ipb-halle/MetFragRelaunched/blob/master/MetFragLib/src/main/resources/MoNA-export-LC-MS.mb (accessed 2024-10-16).

(22) Guha, R. Chemical Informatics Functionality in R. *J Stat Softw* **2007**, *18* (5), 1–16.

(23) Wang, Y.; Backman, T. W. H.; Horan, K.; Girke, T. FmcsR: Mismatch Tolerant Maximum Common Substructure Searching in R. *Bioinformatics* **2013**, *29* (21), 2792–2794. https://doi.org/10.1093/BIOINFORMATICS/BTT475.

(24) Bajusz, D.; Rácz, A.; Héberger, K. Why Is Tanimoto Index an Appropriate Choice for Fingerprint-Based Similarity Calculations? *J Cheminform* **2015**, *7* (1), 1–13. https://doi.org/10.1186/s13321-015-0069-3.

(25) Stein, S. E.; Scott, D. R. Optimization and Testing of Mass Spectral Library Search Algorithms for Compound Identification. *J Am Soc Mass Spectrom* **1994**, *5* (9), 859–866. https://doi.org/10.1016/1044-0305(94)87009-8.

(26) Das, S.; Helmus, R.; Dong, Y.; Beijer, S.; Praetorius, A.; Parsons, J. R.; Jansen, B. Organic Contaminants in Bio-Based Fertilizer Treated Soil: Target and Suspect Screening Approaches. *Chemosphere* **2023**, *337*, 139261. https://doi.org/10.1016/J.CHEMOSPHERE.2023.139261.

(27) Menger, F.; Celma, A.; Schymanski, E. L.; Lai, F. Y.; Bijlsma, L.; Wiberg, K.; Hernández, F.; Sancho, J. V.; Ahrens, L. Enhancing Spectral Quality in Complex Environmental Matrices: Supporting Suspect and Non-Target Screening in Zebra Mussels with Ion Mobility. *Environ Int* **2022**, *170*, 107585. https://doi.org/10.1016/J.ENVINT.2022.107585.

(28) Beijer, A. S.; Das, S.; Helmus, R.; Scheer, P.; Jansen, B.; Slootweg, J. C. Urine as a Biobased Fertilizer: The Netherlands as Case Study. *Sustainability & Circularity NOW* **2024**, *01* (continuous publication). https://doi.org/10.1055/A-2334-6930.

(29) Sepman, H.; Malm, L.; Peets, P.; MacLeod, M.; Martin, J.; Breitholtz, M.; Kruve, A. Bypassing the Identification: MS2Quant for Concentration Estimations of Chemicals Detected with Nontarget LC-HRMS from MS2 Data. *Anal Chem* **2023**, *95* (33), 12329–12338. https://doi.org/10.1021/acs.analchem.3c01744.

(30) Sepman, H. *GitHub - kruvelab/MS2Quant*. https://github.com/kruvelab/MS2Quant (accessed 2024-03-25).
