# Supplementary material for: Comprehensive Mass Spectrometry Workflows to Systematically Elucidate Transformation Processes of Organic Micropollutants: A Case Study on the Photodegradation of Four Pharmaceuticals
Source: Environ Sci Technol. 2025 Feb 14;59(7):3723–36. doi: 10.1021/acs.est.4c09121 (PMC11866921; doi:10.1021/acs.est.4c09121)
Supplement: Supplementary file 2 — es4c09121_si_002.pdf [file es4c09121_si_002.pdf]

Report R1: Overview of transformation product screening results

Rick Helmus‡*[1], Ingrida Bagdonaite‡[1,2,3,4], Pim de Voogt[1], Maarten R. van Bommel[3,4,5], Emma L. Schymanski[6], Annemarie P. van Wezel[1], Thomas L. ter Laak[1,7]

[1] Institute for Biodiversity and Ecosystem Dynamics, University of Amsterdam, Science Park 904, 1098 XH Amsterdam, the Netherlands
[2] Amsterdam Institute for Life and Environment, Vrije Universiteit Amsterdam, De Boelelaan 1108, 1081 HZ, Amsterdam, the Netherlands
[3] Analytical-Chemistry Group, van 't Hoff Institute for Molecular Sciences, University of Amsterdam, Science Park 904, 1098 XH Amsterdam, the Netherlands
[4] Centre for Analytical Sciences Amsterdam, Science Park 904, 1098 XH Amsterdam, the Netherlands
[5] Amsterdam School for Heritage, Memory and Material Culture, Conservation and Restoration of Cultural Heritage, University of Amsterdam, P.O. Box 94522, 1090 GN, Amsterdam, the Netherlands
[6] Luxembourg Centre for Systems Biomedicine, University of Luxembourg, 6 avenue du Swing, L-4367 Belvaux, Luxembourg
[7] KWR Water Research Institute, Groningenhaven 7, 3430 BB, Nieuwegein, the Netherlands

‡ These authors contributed equally.
* Corresponding Author: r.helmus@uva.nl

1 Introduction 2
- 1.1 Data for candidate TP features 2
- 1.2 Data for annotation candidates 3
- 1.3 Additional notes and limitations 4

2 Candidates from structure suspect screening 5
- 2.1 Parent 'flecainide' 5
- 2.2 Parent 'metoprolol' 10
- 2.3 Parent 'sulfamethoxazole' 38
- 2.4 Parent 'phenazone' 51

3 Candidates from formula suspect screening 53
- 3.1 Parent 'metoprolol' 53

4 Candidates for unknowns from compound annotations 55
- 4.1 Parent 'sulfamethoxazole' 55
- 4.2 Parent 'phenazone' 58

5 Candidates for unknowns from formula annotations 61
- 5.1 Parent 'flecainide' 61
- 5.2 Parent 'phenazone' 62

References 68

1 Introduction

This document summarizes the results of the transformation product (TP) screening workflows. The document was automatically generated with knitr[1–3], Rmarkdown[4–6] and pagedown[7], and converted from HTML to PDF format with Chrome[8]. The full code to generate this report and a complete listing of all used software is available in[9].

The sections in this document are organized as follows:

- First level: the screening workflow approach (structure/formula suspect screening and structure/formula unknowns)
- Second level: the parent compound (flecainide, metoprolol, sulfamethoxazole or phenazone)
- Third level: candidate TP feature
- Fourth level: annotation candidates for the TP feature

1.1 Data for candidate TP features

The candidate TP feature sections are named by the feature name assigned by patRoon, which is formatted as MX_RY_Z, with X being the m/z (rounded to zero decimal places), Y the retention time in seconds (rounded to zero decimal places) and Z a unique number to discriminate features with equal X/Y. The candidate TP feature sections include the following data:

- **Chromatogram**. Displays the peak of the feature from the sample with the highest intensity.
- **Regression plot**. A scatter plot with regression lines of normalized feature intensity *versus* initial parent concentration from the mix experiments with significant regression.
- **Experimental table**. Summarizes relative intensities in the experiments with parent mixture (‘mix’), single parents (‘single’) and dark controls (‘dark’). Furthermore, the table contains the correlation coefficient (R^2) and significance (p) for the tested initial parent concentrations of mix experiments. Each row describes the data for an experiment, and is greyed out if the observed regression was insignificant. Empty cells indicate no observations were available (e.g. feature intensity below threshold). Experimental remarks are shown as superscript numbers, which are explained in Table 1.

Table 1. Description of remarks that are referred to inside the experimental tables.

Remark	Description
#1	Features removed during post-processing due to high intensity deviation in replicates (>75% RSD).
#2	Features removed during post-processing as some were missing in replicates.
#3	The feature intensity was below set thresholds and therefore not detected or post-filtered.
#4	Poor regression due to high variability caused by low intensity features.
#5	Poor regression due to an outlier.
#6	Poor regression due to intensity variability caused by poor chromatography.
#7	There were two very closely eluting features observed with the same *m/z* in all experiments. The feature detection algorithm only assigned one of the two features in each sample, and which one was assigned varied across samples. As a result, a negative slope was observed for U and poor regression for UH. Nevertheless, the MS^2 data used for annotation was consistently taken from the second feature, hence, the annotation results reported here only concern this feature.
#8	The reported intensity is less accurate due to wrong peak assignment in one out of the two replicates.

1.2 Data for annotation candidates

The annotation candidates are automatically named with a format consisting of four parts:

```
[SuS|SuF|UnC|UnF]-[FLE|MET|SMX|PHE]-MXXX-Y
```

The four parts are:

1. the screening type
   - SuS, SuF: suspect screening of structures or formulas, respectively
   - UnC, UnF: screening of unknowns from compound and formula candidates, respectively
2. the parent compound
   - FLE: flecainide
   - MET: metoprolol
   - SMX: sulfamethoxazole
   - PHE: phenazone
3. the *m/z* rounded to zero decimals
4. a unique numeric identifier to discern TPs with equal rounded mass

For instance, **SuF-MET-M302-1** is the first unique TP candidate with *m/z* 302 for metoprolol and found by structure suspect screening.

The annotation candidate sections include the following data:

- **Structural transformation** (only for candidates with structure information). This includes highlights of atoms and chemical bonds removed from the parent (greyed out) or different in the TP (marked yellow).
- **Formula transformation** (only for candidates without structure information).
- **Summary table**. Summarizes key chemistry, transformation and annotation properties (described in Table 2).
- **Annotated MS^2 spectrum** (if available). The mass peaks are marked as explained by compound annotation ('metfrag'), formula annotation ('genform'), both ('metfrag,genform'), the unfragmented precursor ion ('precursor') or otherwise unexplained ('unassigned'). Mass peaks are annotated with fragment formulas if available. The *m/z* axis of the spectrum is zoomed in on the detected MS^2 peaks to improve the readability of the formula annotations.

Table 2. Property descriptions for the summary tables in the annotation candidate sections.

Property	Description
Formula	The formula of the TP and its difference to the parent.
SMILES	The SMILES of the TP (if available).
m/z error	The difference between the observed and expected *m/z*.
XLog P	The Log P value (calculated as XLog P with rcdk[10]) and its difference to the parent.
Data source(s)	Data sources such as prediction algorithm, literature references and PubChem[11] identifiers.
In silico similarity	The cosine similarity between the MS^2 spectrum with only annotated peaks and the full MS^2 spectrum. Reported for formula annotations and compound annotations (if available).
ID confidence level	The identification confidence level assigned as described in Supporting Information section S1.9.
Fit, TP_sim, TP_score	TP similarity and scoring values, as described in the Materials & Method section.
Other matches	Names of features that were also matched with this candidate structure/formula.

1.3 Additional notes and limitations

The graphics for molecular transformation highlight changes in aromaticity, which may lead to unexpected markings in some cases (see e.g. *SuS-MET-M282-5*). Furthermore, since the software used to depict the molecular structures lacks support to fully align parent and TP structures, the orientation of these may differ.

2 Candidates from structure suspect screening

2.1 Parent ‘flecainide’

2.1.1 Feature ‘M147_R255_4120’

RT: 4.2 (Δ -4.1) min; m/z: 147.1128 (Δ -268.0335)

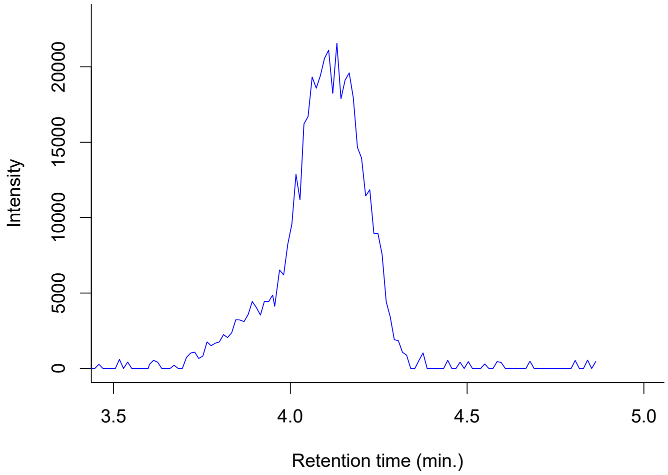

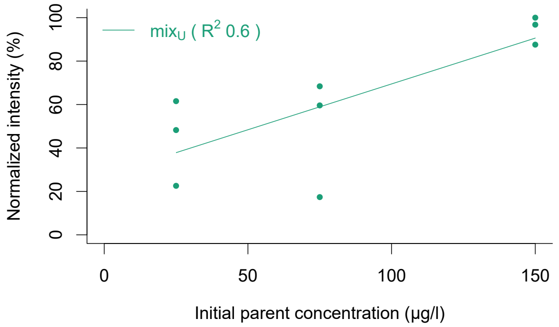

Condition	mix 25	mix 75	mix 150	RSQ	p	slope	single	dark
U[6]	44%	48%	95%	0.60	0.015	0.42%		
UH								
UHN			28%					

2.1.1.1 Candidate ‘SuS-FLE-M147-1’

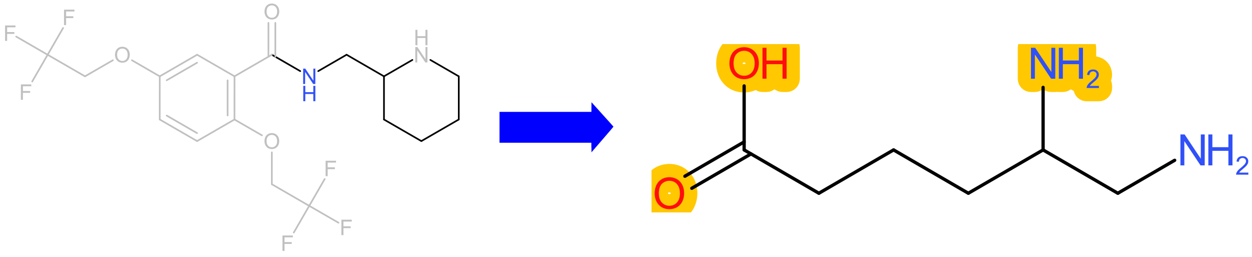

Formula	$C_6H_{14}N_2O_2$ (Δ -$C_{11}H_6OF_6$)
SMILES	C(CC(=O)O)CC(N)CN
m/z error	+0.0 mDa
XLog P	-1.0 (Δ -5.7)
Data source(s)	BTE
In silico similarity	0.00 (formula)
ID confidence level	4c
Fit	$fit_{formula}$: 1.00<br>$fit_{compound}$: 0.70

No MS/MS annotations

2.1.2 Feature ‘M333_R474_5934’

RT: 7.9 (Δ -0.4) min; m/z: 333.1417 (Δ -82.0046)

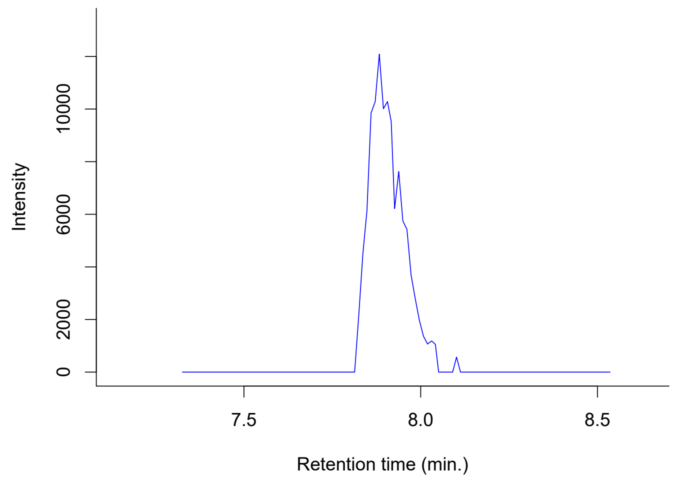

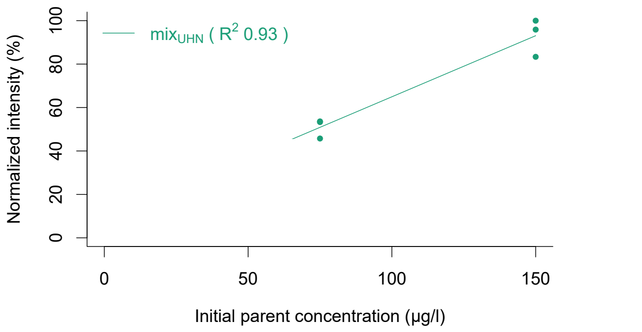

Condition	mix 25	mix 75	mix 150	RSQ	p	slope	single	dark
U								
UH								
UHN		51%	93%	0.93	0.002	0.56%	39%	

2.1.2.1 Candidate ‘SuS-FLE-M333-1’

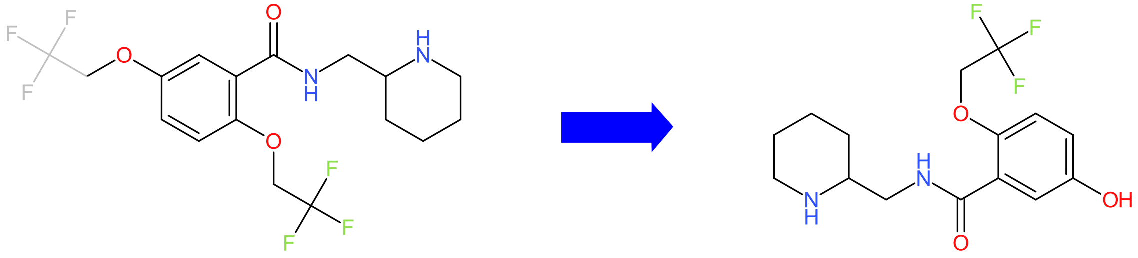

Formula	$C_{15}H_{19}F_3N_2O_3$ (Δ $-C_2HF_3$)
SMILES	Oc1ccc(OCC(F)(F)F)c(c1)C(=O)NCC1CCCCN1
m/z error	-0.3 mDa
XLog P	2.9 (Δ -1.7)
Data source(s)	CTS, BTE, BTH
In silico similarity	0.00 (formula)
ID confidence level	5
Fit	$fit_{formula}$: 1.00<br>$fit_{compound}$: 1.00
Other matches	M333_R393_6915

No MS/MS annotations

2.1.2.2 Candidate 'SuS-FLE-M333-2'

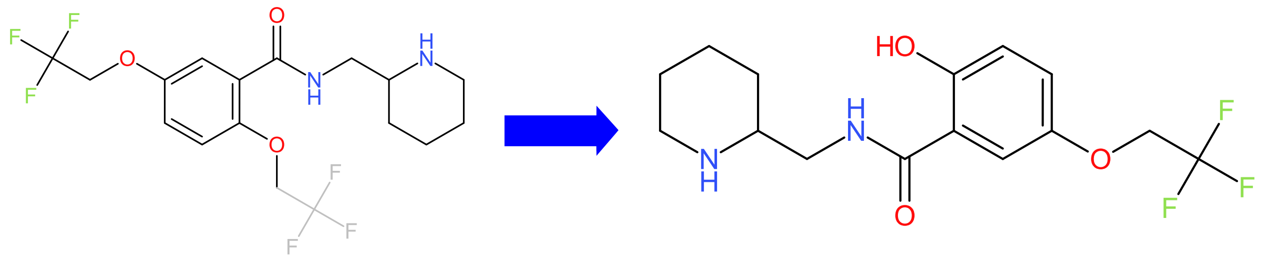

Formula	$C_{15}H_{19}F_3N_2O_3$ (Δ -C_2HF_3)
SMILES	Oc1ccc(OCC(F)(F)F)cc1C(=O)NCC1CCCCN1
m/z error	-0.3 mDa
XLog P	3.7 (Δ -0.9)
Data source(s)	CTS, BTE, BTH
In silico similarity	0.00 (formula)
ID confidence level	5
Fit	$fit_{formula}$: 1.00<br>$fit_{compound}$: 1.00
Other matches	M333_R393_6915

No MS/MS annotations

2.1.3 Feature ‘M333_R393_6915’

RT: 6.6 (Δ -1.8) min; m/z: 333.1423 (Δ -82.0040)

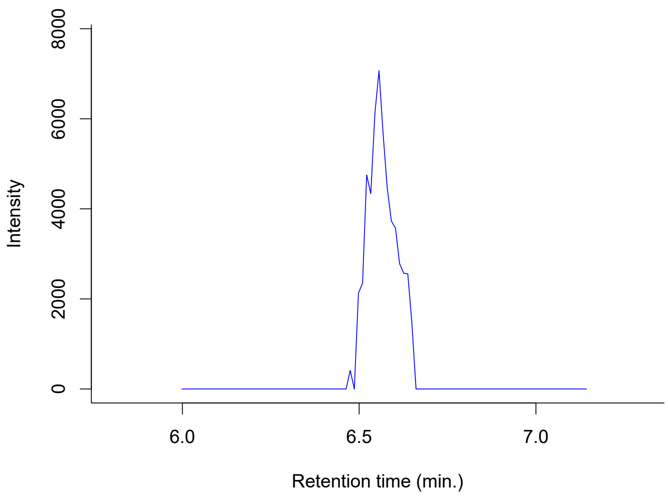

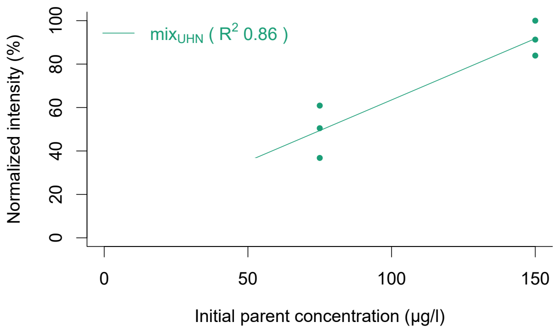

Condition	mix 25	mix 75	mix 150	RSQ	p	slope	single	dark
U								
UH								
UHN		49%	92%	0.86	0.007	0.56%	65%	

2.1.3.1 Candidate ‘SuS-FLE-M333-1’

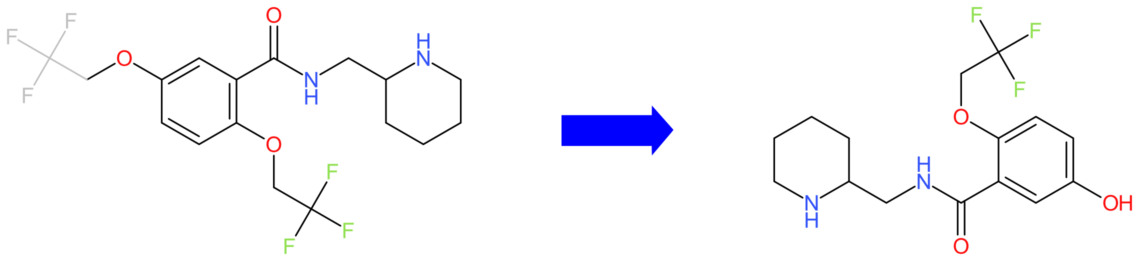

Formula	$C_{15}H_{19}F_3N_2O_3$ (Δ -C_2HF_3)
SMILES	Oc1ccc(OCC(F)(F)F)c(c1)C(=O)NCC1CCCCN1
m/z error	+0.2 mDa
XLog P	2.9 (Δ -1.7)
Data source(s)	CTS, BTE, BTH
In silico similarity	0.00 (formula)
ID confidence level	5
Fit	$fit_{formula}$: 1.00<br>$fit_{compound}$: 1.00
Other matches	M333_R474_5934

No MS/MS annotations

2.1.3.2 Candidate 'SuS-FLE-M333-2'

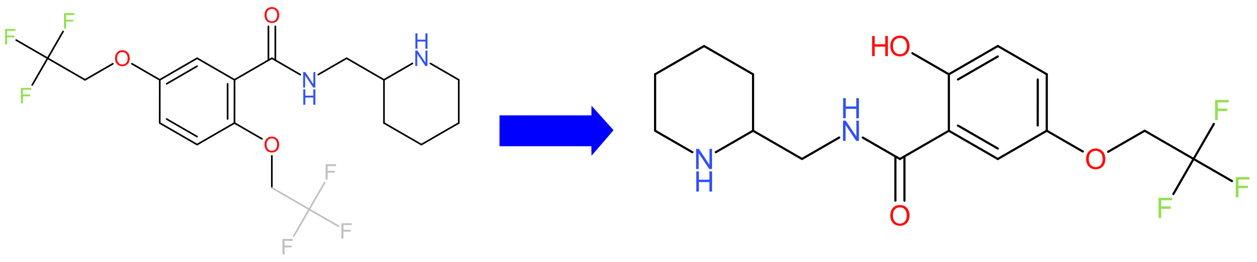

Formula	$C_{15}H_{19}F_3N_2O_3$ (Δ -C_2HF_3)
SMILES	Oc1ccc(OCC(F)(F)F)cc1C(=O)NCC1CCCCN1
m/z error	+0.2 mDa
XLog P	3.7 (Δ -0.9)
Data source(s)	CTS, BTE, BTH
In silico similarity	0.00 (formula)
ID confidence level	5
Fit	$fit_{formula}$: 1.00<br>$fit_{compound}$: 1.00
Other matches	M333_R474_5934

No MS/MS annotations

2.2 Parent ‘metoprolol’

2.2.1 Feature ‘M74_R251_2797’

RT: 4.2 (Δ -2.7) min; m/z: 74.0589 (Δ -194.1328)

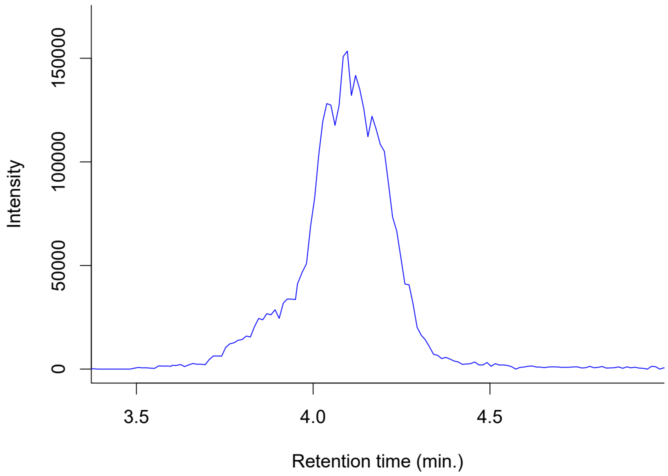

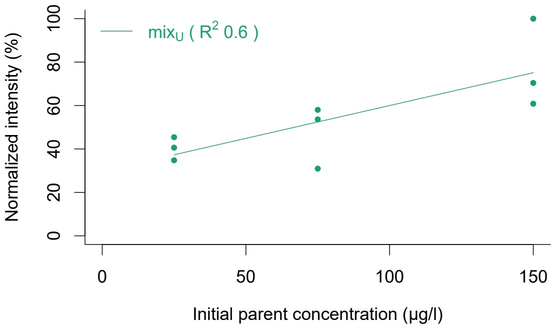

Condition	mix 25	mix 75	mix 150	RSQ	p	slope	single	dark
U[6]	40%	48%	77%	0.60	0.014	0.3%		2%
UH			[2]					4%
UHN			29%					3%

2.2.1.1 Candidate ‘SuS-MET-M74-1’

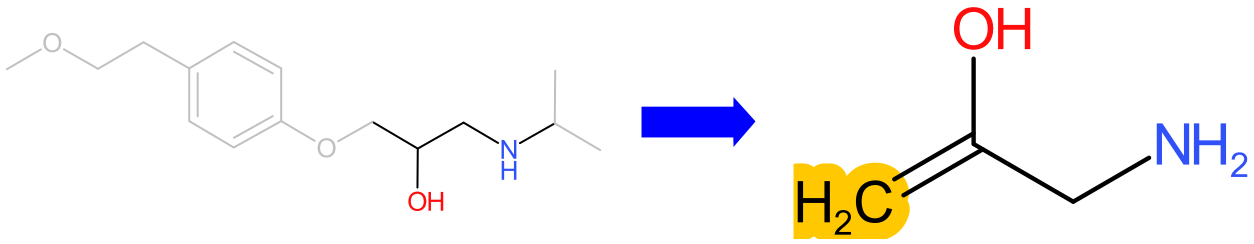

Formula	C_3H_7NO (Δ -$C_{12}H_{18}O_2$)
SMILES	C=C(O)CN
m/z error	-1.1 mDa
XLog P	-0.4 (Δ -2.0)
Data source(s)	LIT (a)
In silico similarity	NA
ID confidence level	5
Fit	$fit_{formula}$: 1.00<br>$fit_{compound}$: 0.80

No MS/MS annotations

2.2.2 Feature 'M226_R366_2919'

RT: 6.1 (Δ -0.7) min; m/z: 226.1437 (Δ -42.0481)

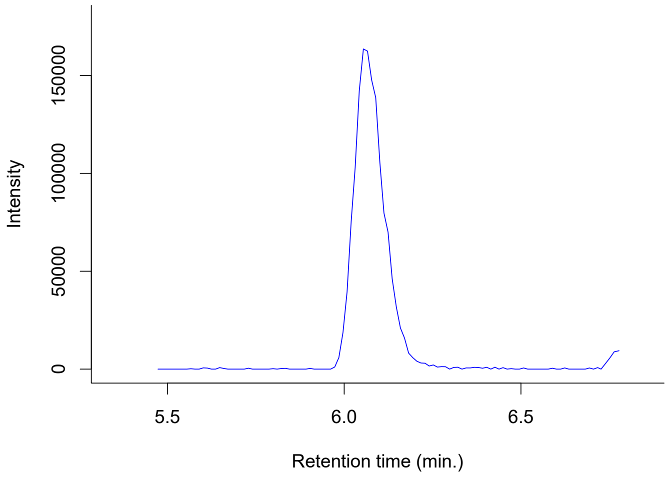

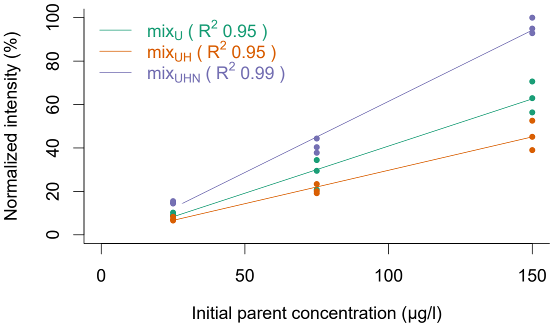

Condition	mix 25	mix 75	mix 150	RSQ	p	slope	single	dark
U	9%	28%	63%	0.95	0.000	0.43%	16%	
UH	7%	21%	46%	0.95	0.000	0.31%	7%	
UHN	15%	41%	96%	0.99	0.000	0.66%	20%	

2.2.2.1 Candidate 'SuS-MET-M226-3'

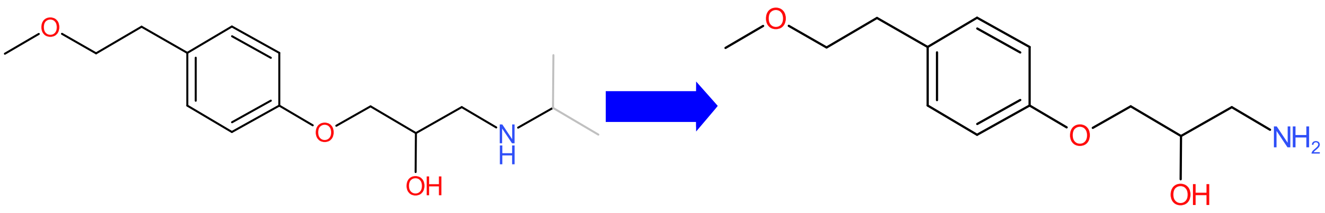

Formula	$C_{12}H_{19}NO_3$ (Δ -C_3H_6)
SMILES	NCC(COC1=CC=C(C=C1)CCOC)O
m/z error	-0.1 mDa
XLog P	0.4 (Δ -1.3)
Data source(s)	BTE, BTH, LIT (a, b, c, d, e, f)
In silico similarity	1.00 (formula), 0.95 (compound)
ID confidence level	3a
Fit	$fit_{formula}$: 1.00<br>$fit_{compound}$: 1.00

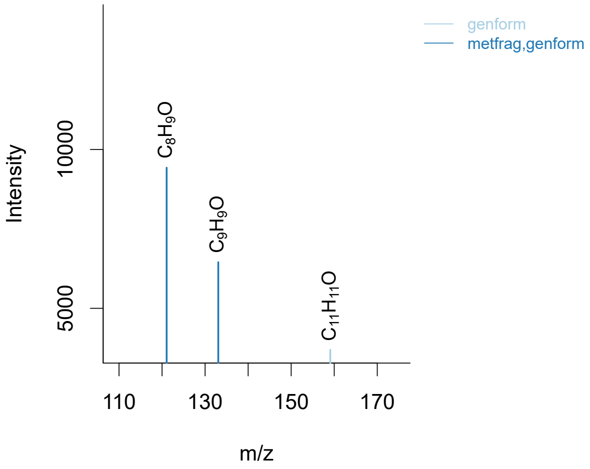

2.2.2.2 Candidate 'SuS-MET-M226-2'

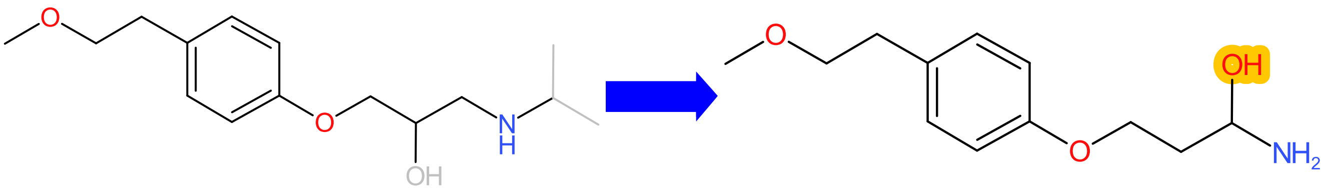

Formula	$C_{12}H_{19}NO_3$ (Δ -C_3H_6)
SMILES	NC(CCOC1=CC=C(C=C1)CCOC)O
m/z error	-0.1 mDa
XLog P	0.8 (Δ -0.8)
Data source(s)	BTE
In silico similarity	1.00 (formula), 0.95 (compound)
ID confidence level	3d
Fit	$fit_{formula}$: 1.00<br>$fit_{compound}$: 0.94

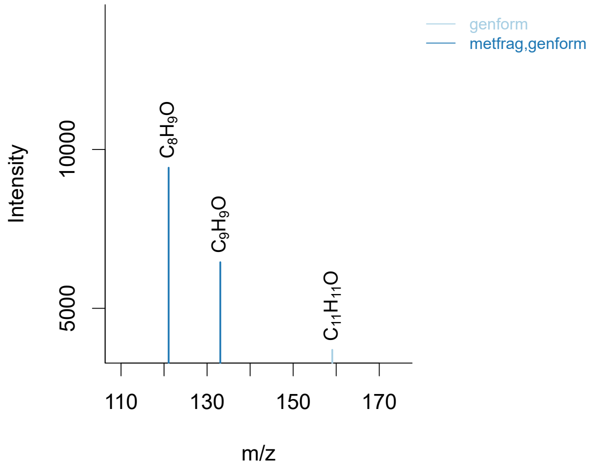

2.2.2.3 Candidate 'SuS-MET-M226-1'

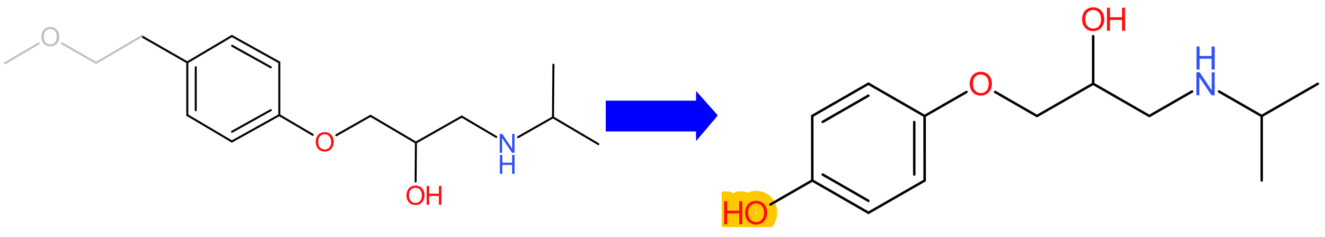

Formula	$C_{12}H_{19}NO_3$ (Δ -C_3H_6)
SMILES	CC(C)NCC(O)COc1ccc(O)cc1
m/z error	-0.1 mDa
XLog P	1.4 (Δ -0.3)
Data source(s)	LIT (a, b)
In silico similarity	1.00 (formula), 0.00 (compound)
ID confidence level	4a
Fit	$fit_{formula}$: 1.00<br>$fit_{compound}$: 0.94

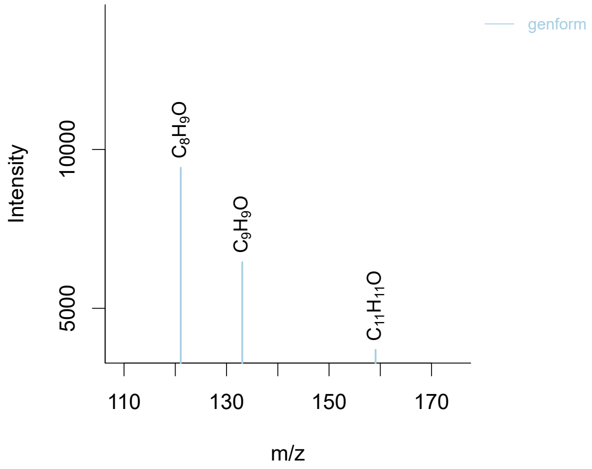

2.2.3 Feature 'M238_R327_2828'

RT: 5.5 (Δ -1.4) min; m/z: 238.1437 (Δ -30.0481)

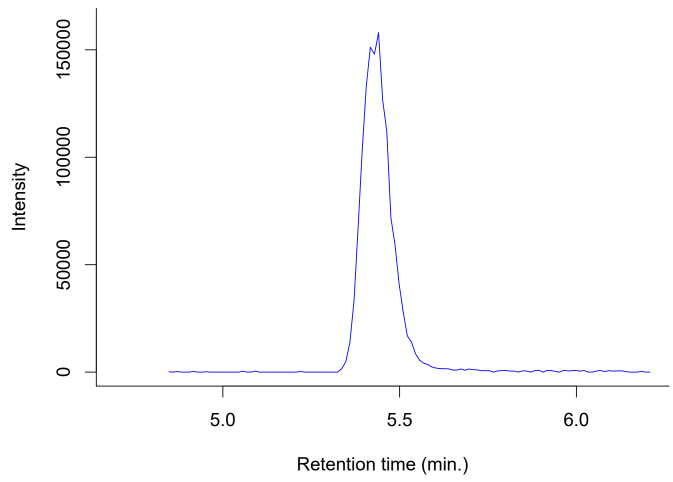

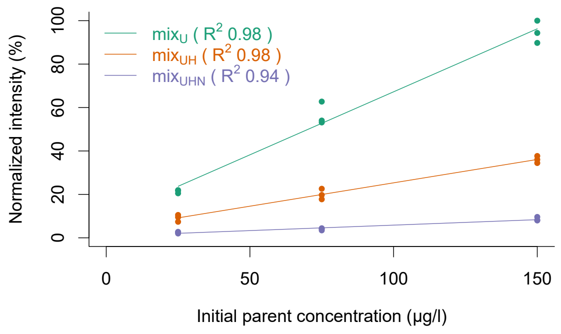

Condition	mix 25	mix 75	mix 150	RSQ	p	slope	single	dark
U	21%	57%	95%	0.98	0.000	0.58%	34%	
UH	9%	20%	36%	0.98	0.000	0.22%	16%	
UHN	2%	4%	9%	0.94	0.000	0.05%	5%	

2.2.3.1 Candidate 'SuS-MET-M238-1'

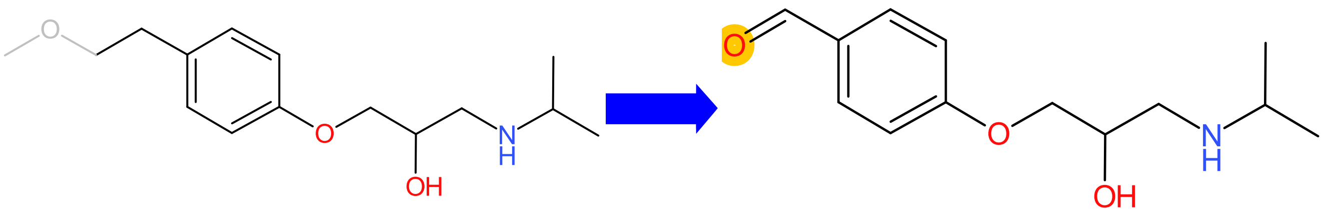

Formula	$C_{13}H_{19}NO_3$ (Δ -C_2H_6)
SMILES	CC(C)NCC(O)COc1ccc(C=O)cc1
m/z error	-0.1 mDa
XLog P	1.5 (Δ -0.2)
Data source(s)	LIT (a, b)
In silico similarity	1.00 (formula), 0.72 (compound)
ID confidence level	3d
Fit	$fit_{formula}$: 1.00<br>$fit_{compound}$: 0.94

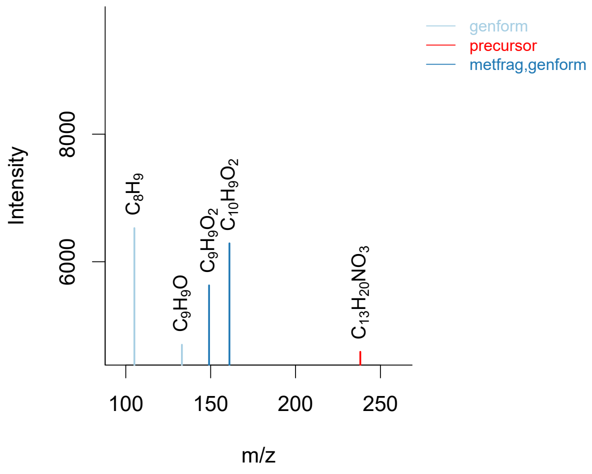

2.2.4 Feature 'M240_R303_6188'

RT: 5.0 (Δ -1.8) min; m/z: 240.1592 (Δ -28.0325)

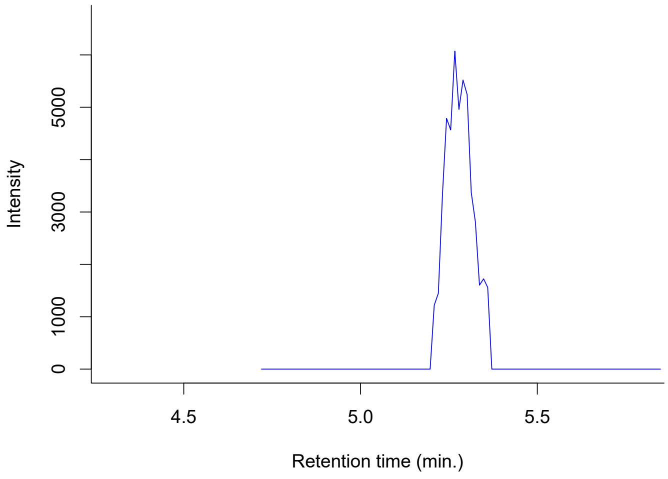

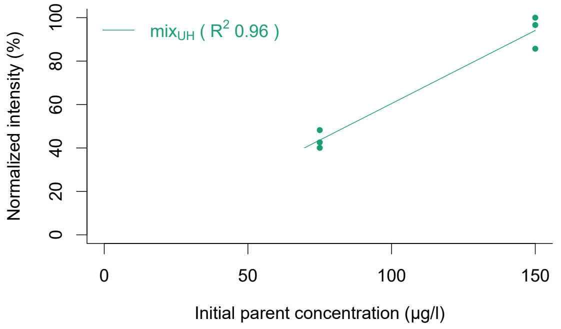

Condition	mix 25	mix 75	mix 150	RSQ	p	slope	single	dark
U								
UH		44%	94%	0.96	0.001	0.67%		
UHN			59%					

2.2.4.1 Candidate 'SuS-MET-M240-1'

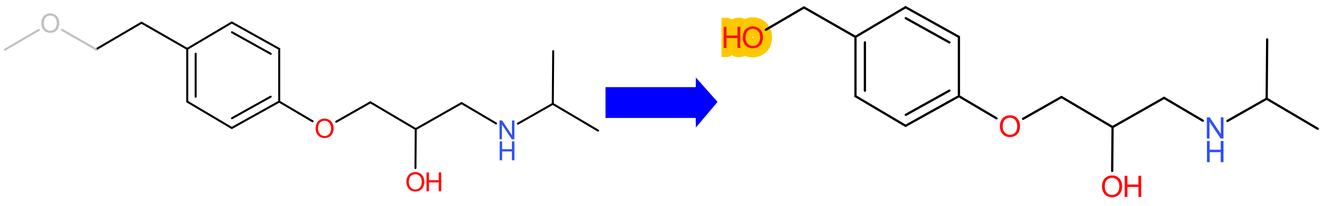

Formula	$C_{13}H_{21}NO_3$ (Δ -C_2H_4)
SMILES	CC(C)NCC(O)COc1ccc(CO)cc1
m/z error	-0.2 mDa
XLog P	1.0 (Δ -0.7)
Data source(s)	LIT (a, b, c, d)
In silico similarity	0.00 (formula), 0.00 (compound)
ID confidence level	5
Fit	$fit_{formula}$: 1.00<br>$fit_{compound}$: 0.94

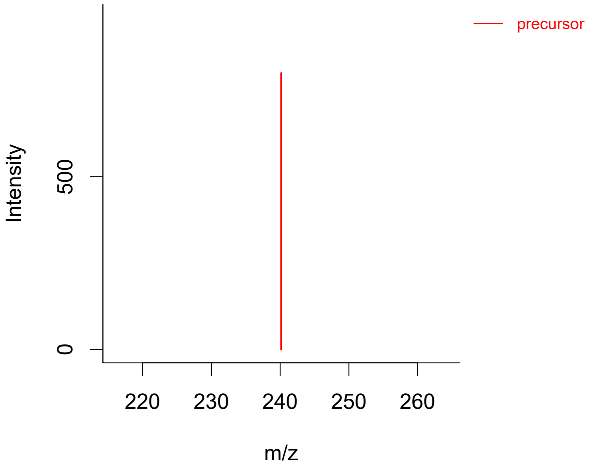

2.2.5 Feature ‘M252_R350_3882’

RT: 5.8 (Δ -1.0) min; m/z: 252.1593 (Δ -16.0324)

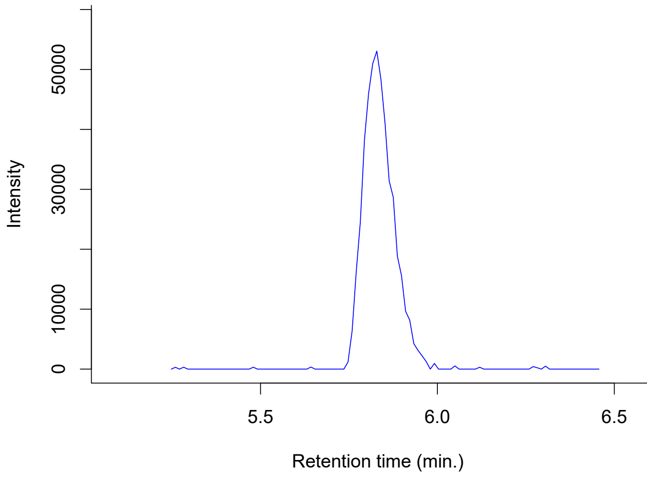

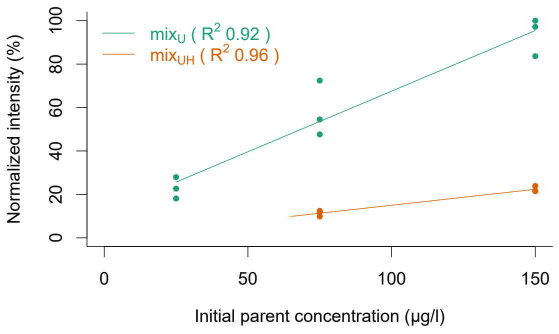

Condition	mix 25	mix 75	mix 150	RSQ	p	slope	single	dark
U	23%	58%	94%	0.92	0.000	0.56%	49%	
UH		11%	22%	0.96	0.001	0.15%	15%	
UHN			6%					

2.2.5.1 Candidate ‘SuS-MET-M252-1’

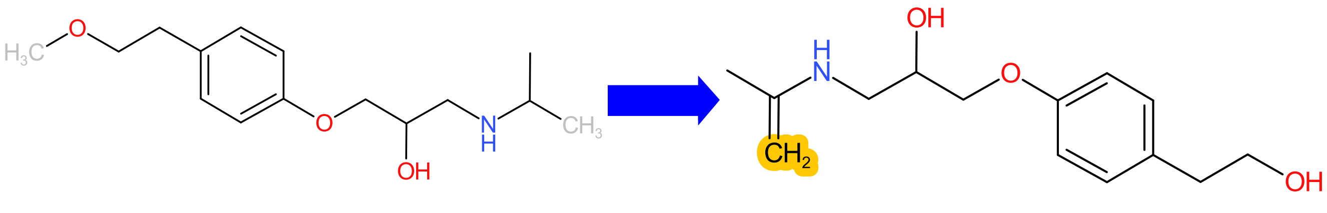

Formula	$C_{14}H_{21}NO_3$ (Δ -CH_4)
SMILES	C=C(C)NCC(COC1=CC=C(C=C1)CCO)O
m/z error	-0.1 mDa
XLog P	1.2 (Δ -0.4)
Data source(s)	BTH
In silico similarity	1.00 (formula), 0.00 (compound)
ID confidence level	4a
Fit	$fit_{formula}$: 1.00<br>$fit_{compound}$: 0.94

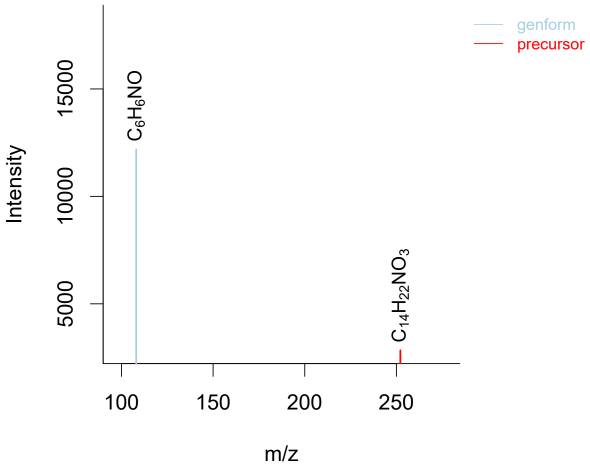

2.2.5.2 Candidate 'SuS-MET-M252-2'

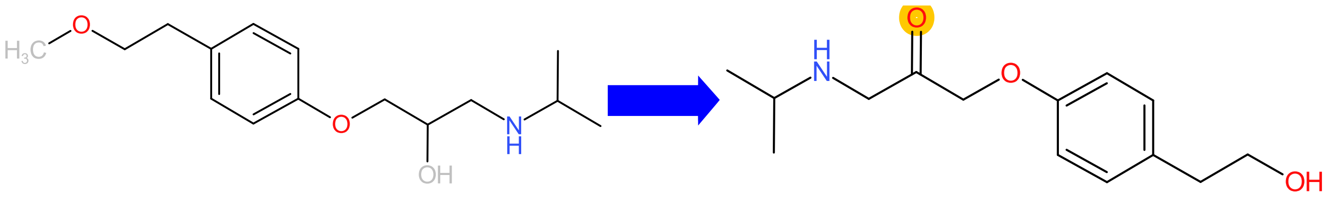

Formula	$C_{14}H_{21}NO_3$ (Δ -CH_4)
SMILES	CC(C)NCC(COC1=CC=C(C=C1)CCO)=O
m/z error	-0.1 mDa
XLog P	1.1 (Δ -0.5)
Data source(s)	BTE, BTH
In silico similarity	1.00 (formula), 0.00 (compound)
ID confidence level	4a
Fit	$fit_{formula}$: 1.00<br>$fit_{compound}$: 0.94

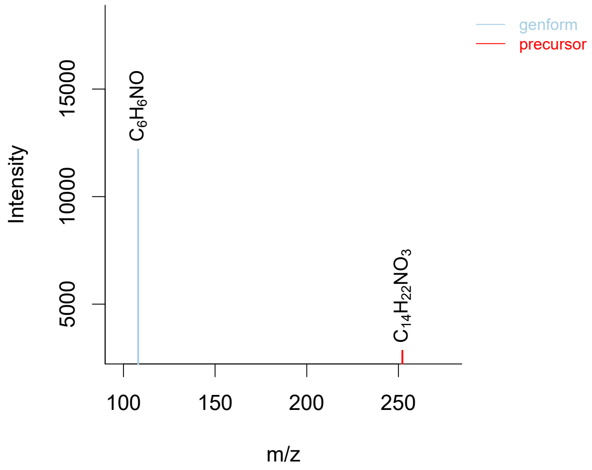

2.2.5.3 Candidate 'SuS-MET-M252-3'

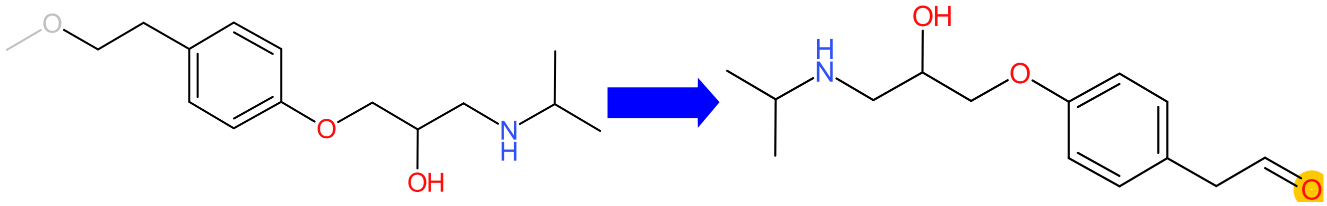

Formula	$C_{14}H_{21}NO_3$ (Δ -CH_4)
SMILES	CC(C)NCC(COC1=CC=C(C=C1)CC=O)O
m/z error	-0.1 mDa
XLog P	1.6 (Δ -0.0)
Data source(s)	BTE, BTH, LIT (a, b, c, d)
In silico similarity	1.00 (formula), 0.00 (compound)
ID confidence level	4a
Fit	$fit_{formula}$: 1.00<br>$fit_{compound}$: 0.94

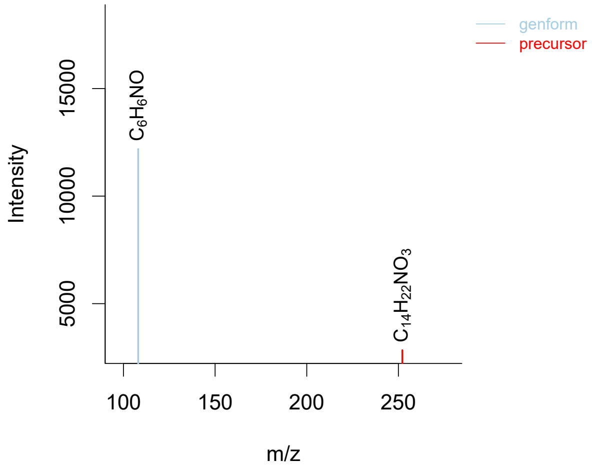

2.2.5.4 Candidate 'SuS-MET-M252-4'

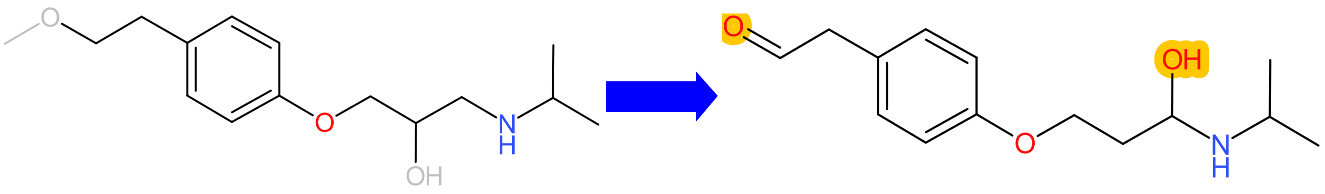

Formula	$C_{14}H_{21}NO_3$ (Δ -CH_4)
SMILES	CC(C)NC(CCOC1=CC=C(C=C1)CC=O)O
m/z error	-0.1 mDa
XLog P	2.1 (Δ +0.5)
Data source(s)	BTE
In silico similarity	1.00 (formula), 0.00 (compound)
ID confidence level	4a
Fit	$fit_{formula}$: 1.00<br>$fit_{compound}$: 0.89

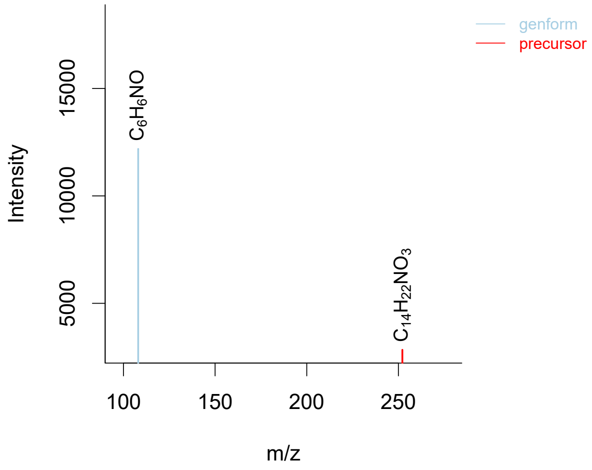

2.2.6 Feature ‘M254_R347_8601’

RT: 5.8 (Δ -1.1) min; m/z: 254.1384 (Δ -14.0534)

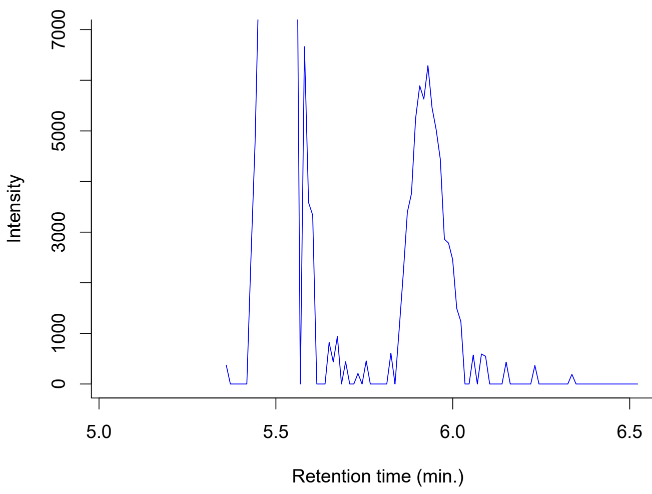

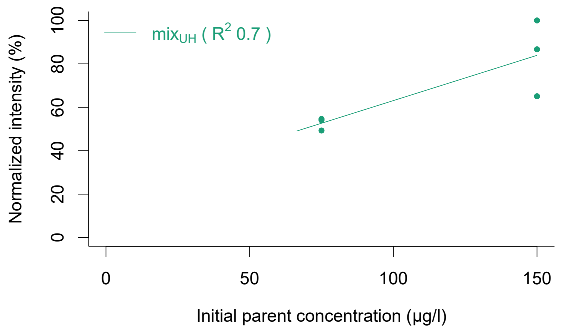

Condition	mix 25	mix 75	mix 150	RSQ	p	slope	single	dark
U								
UH		53%	84%	0.70	0.039	0.42%		
UHN								

2.2.6.1 Candidate ‘SuS-MET-M254-1’

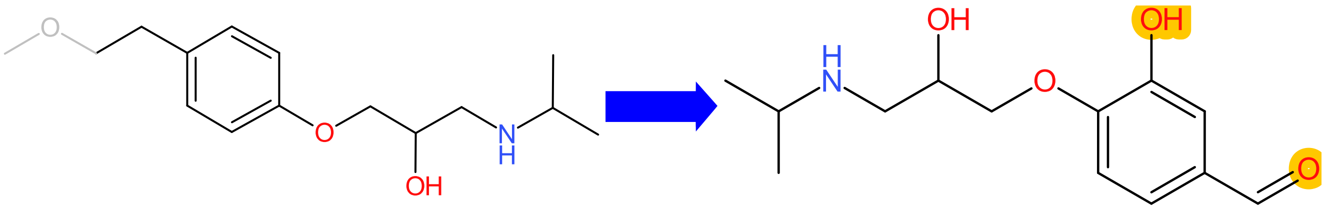

Formula	$C_{13}H_{19}NO_4$ (Δ $-C_2H_6$ +O)
SMILES	CC(C)NCC(O)COc1ccc(C=O)cc1O
m/z error	-0.3 mDa
XLog P	0.8 (Δ -0.8)
Data source(s)	LIT (a, b)
In silico similarity	0.96 (formula), 0.27 (compound)
ID confidence level	4a
Fit	$fit_{formula}$: 0.97<br>$fit_{compound}$: 0.89
Other matches	M254_R321_3770

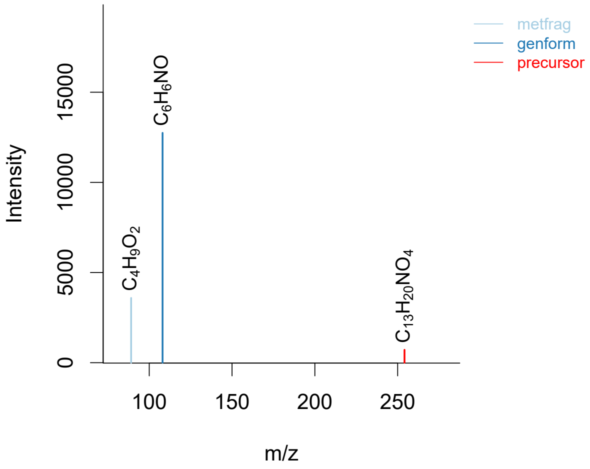

2.2.6.2 Candidate 'SuS-MET-M254-3'

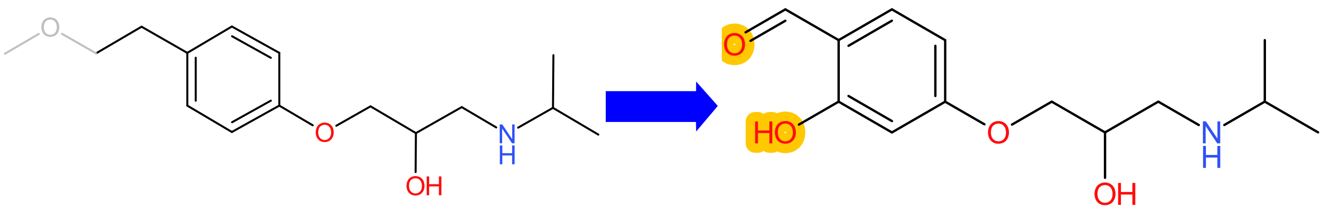

Formula	$C_{13}H_{19}NO_4$ (Δ -C_2H_6 +O)
SMILES	CC(C)NCC(O)COc1ccc(C=O)c(O)c1
m/z error	-0.3 mDa
XLog P	1.5 (Δ -0.1)
Data source(s)	LIT (a, b)
In silico similarity	0.96 (formula), 0.27 (compound)
ID confidence level	4a
Fit	$\text{fit}_{\text{formula}}$: 0.97<br>$\text{fit}_{\text{compound}}$: 0.89
Other matches	M254_R321_3770

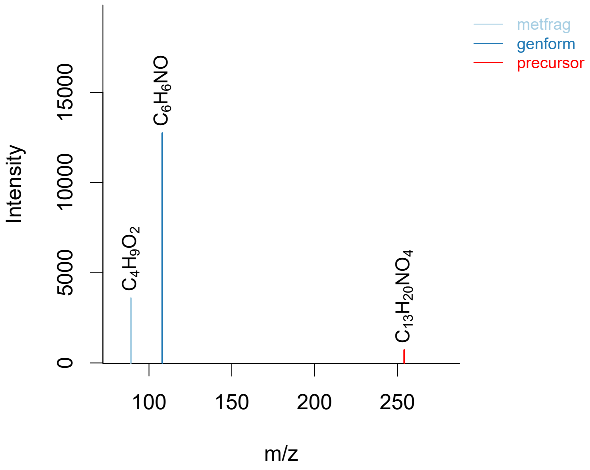

2.2.6.3 Candidate 'SuS-MET-M254-4'

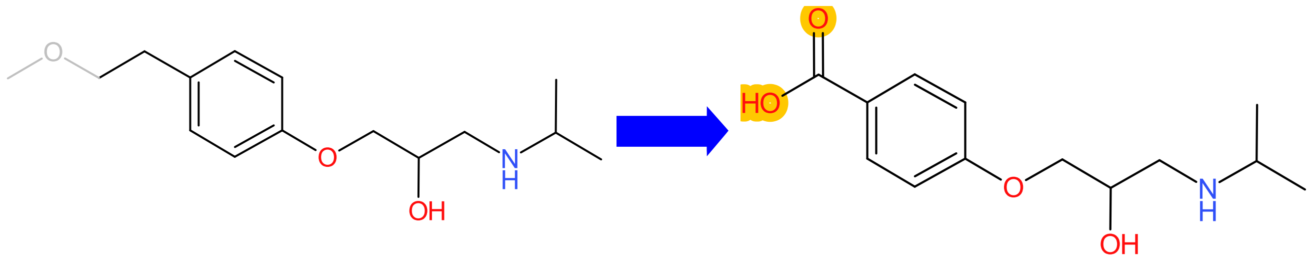

Formula	$C_{13}H_{19}NO_4$ (Δ -C_2H_6 +O)
SMILES	CC(C)NCC(O)COc1ccc(C(=O)O)cc1
m/z error	-0.3 mDa
XLog P	1.4 (Δ -0.3)
Data source(s)	LIT (a)
In silico similarity	0.96 (formula), 0.27 (compound)
ID confidence level	4a
Fit	$\text{fit}_{\text{formula}}$: 0.97<br>$\text{fit}_{\text{compound}}$: 0.89
Other matches	M254_R321_3770

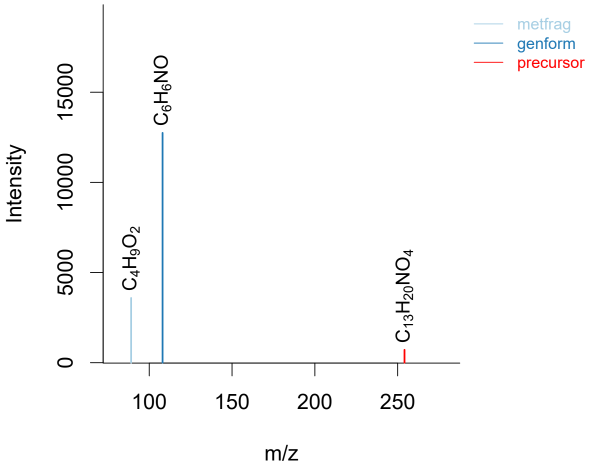

2.2.7 Feature 'M254_R321_3770'

RT: 5.3 (Δ -1.5) min; m/z: 254.1386 (Δ -14.0532)

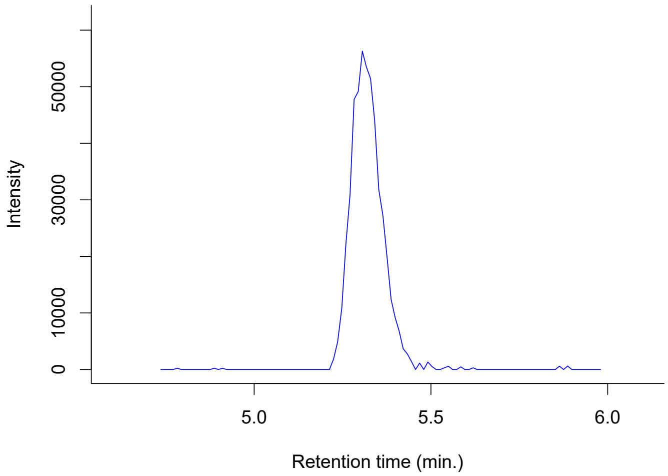

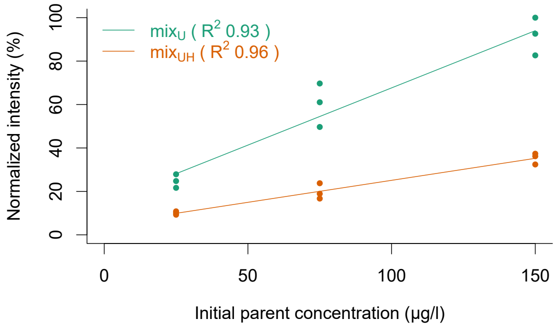

Condition	mix 25	mix 75	mix 150	RSQ	p	slope	single	dark
U	25%	60%	92%	0.93	0.000	0.53%	8%	
UH	10%	20%	35%	0.96	0.000	0.2%		
UHN			9%					

2.2.7.1 Candidate 'SuS-MET-M254-1'

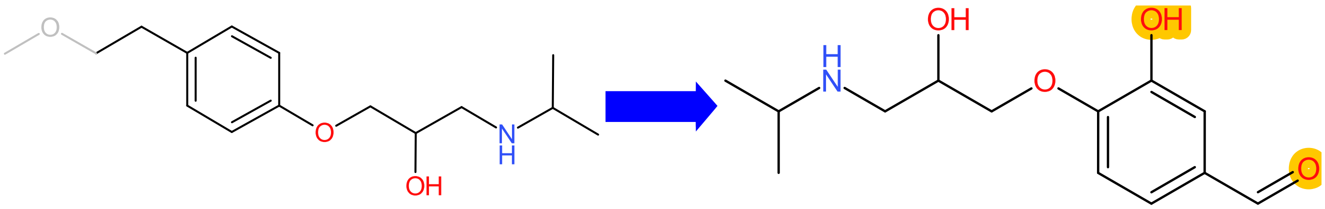

Formula	$C_{13}H_{19}NO_4$ (Δ $-C_2H_6$ +O)
SMILES	CC(C)NCC(O)COc1ccc(C=O)cc1O
m/z error	-0.1 mDa
XLog P	0.8 (Δ -0.8)
Data source(s)	LIT (a, b)
In silico similarity	1.00 (formula), 0.85 (compound)
ID confidence level	3d
Fit	$fit_{formula}$: 0.97<br>$fit_{compound}$: 0.89
Other matches	M254_R347_8601

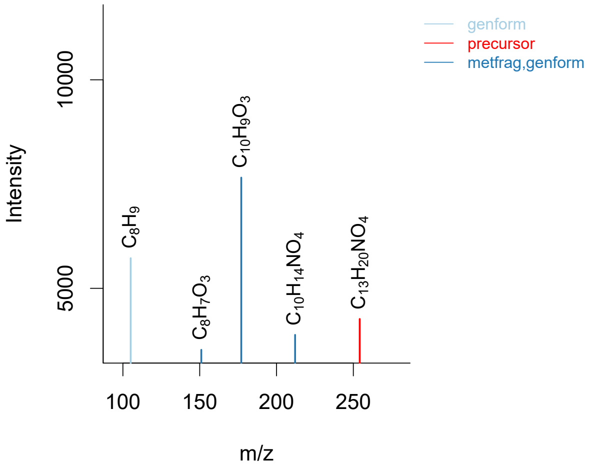

2.2.7.2 Candidate 'SuS-MET-M254-3'

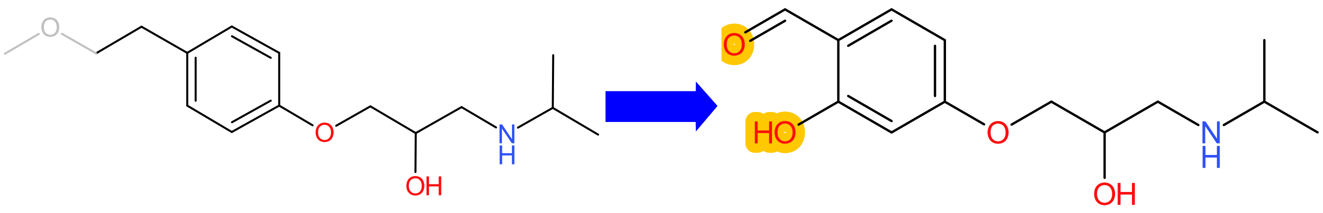

Formula	$C_{13}H_{19}NO_4$ (Δ $-C_2H_6$ +O)
SMILES	CC(C)NCC(O)COc1ccc(C=O)c(O)c1
m/z error	-0.1 mDa
XLog P	1.5 (Δ -0.1)
Data source(s)	LIT (a, b)
In silico similarity	1.00 (formula), 0.85 (compound)
ID confidence level	3d
Fit	$fit_{formula}$: 0.97<br>$fit_{compound}$: 0.89
Other matches	M254_R347_8601

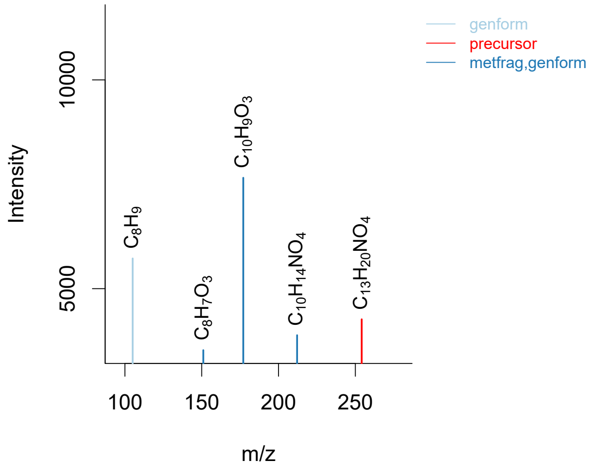

2.2.7.3 Candidate 'SuS-MET-M254-4'

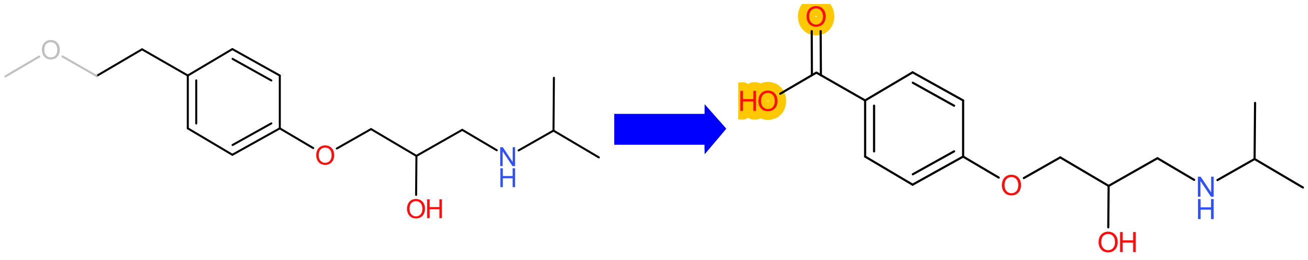

Formula	$C_{13}H_{19}NO_4$ (Δ $-C_2H_6$ +O)
SMILES	CC(C)NCC(O)COc1ccc(C(=O)O)cc1
m/z error	-0.1 mDa
XLog P	1.4 (Δ -0.3)
Data source(s)	LIT (a)
In silico similarity	1.00 (formula), 0.85 (compound)
ID confidence level	3d
Fit	$fit_{formula}$: 0.97<br>$fit_{compound}$: 0.89
Other matches	M254_R347_8601

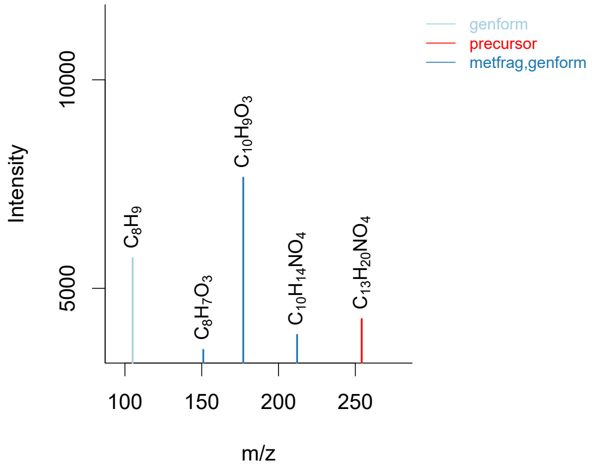

2.2.8 Feature 'M254_R336_4164'

RT: 5.6 (Δ -1.2) min; m/z: 254.1750 (Δ -14.0168)

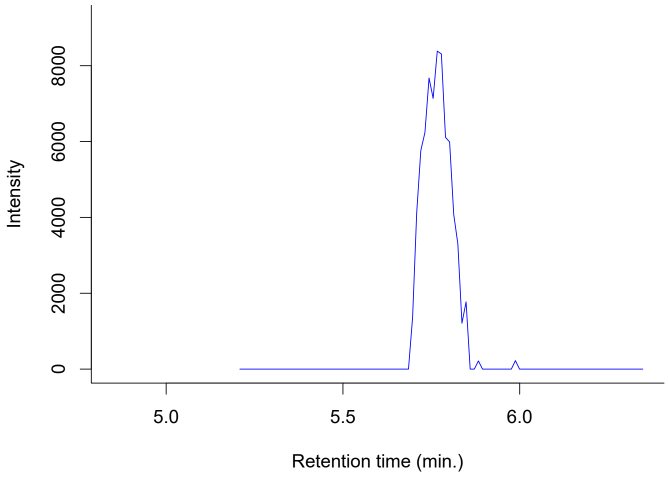

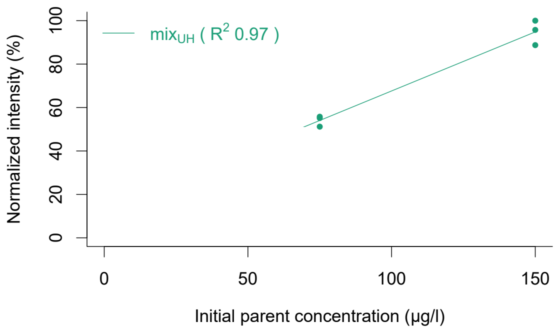

Condition	mix 25	mix 75	mix 150	RSQ	p	slope	single	dark
U			55%				40%	
UH		54%	95%	0.97	0.000	0.54%	41%	
UHN			56%				44%	

2.2.8.1 Candidate 'SuS-MET-M254-2'

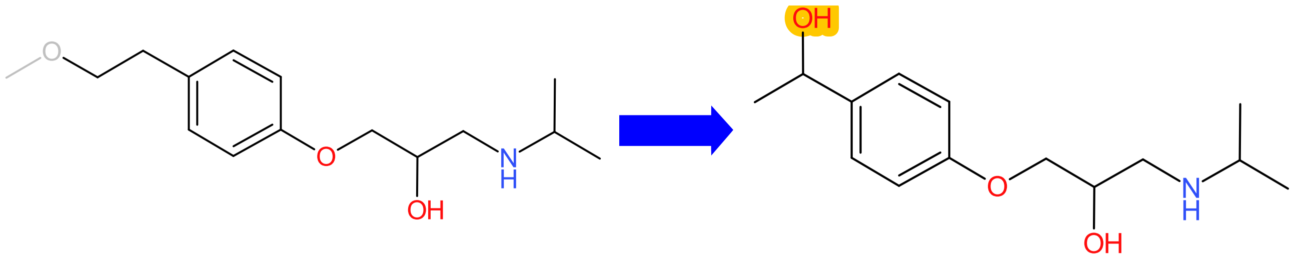

Formula	$C_{14}H_{23}NO_3$ (Δ -CH_2)
SMILES	CC(C)NCC(O)COc1ccc(C(C)O)cc1
m/z error	-0.1 mDa
XLog P	1.5 (Δ -0.2)
Data source(s)	LIT (a)
In silico similarity	0.00 (formula), 0.00 (compound)
ID confidence level	5
Fit	$fit_{formula}$: 1.00<br>$fit_{compound}$: 0.94

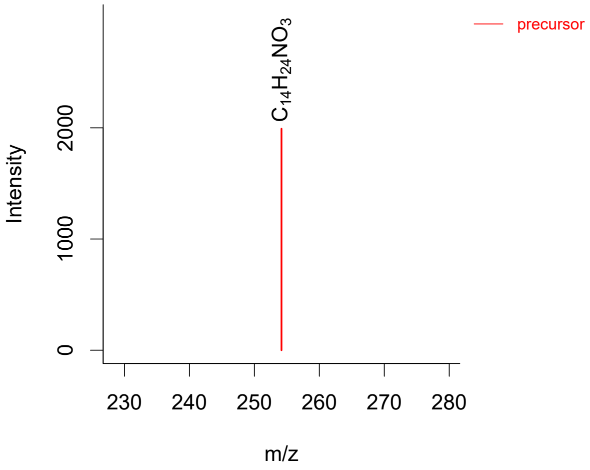

2.2.8.2 Candidate 'SuS-MET-M254-5'

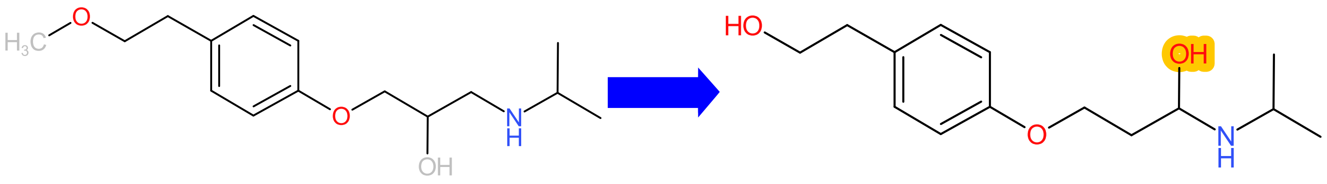

Formula	$C_{14}H_{23}NO_3$ (Δ -CH_2)
SMILES	CC(C)NC(CCOC1=CC=C(C=C1)CCO)O
m/z error	-0.1 mDa
XLog P	1.6 (Δ -0.0)
Data source(s)	BTE
In silico similarity	0.00 (formula), 0.00 (compound)
ID confidence level	5
Fit	$fit_{formula}$: 1.00<br>$fit_{compound}$: 0.94

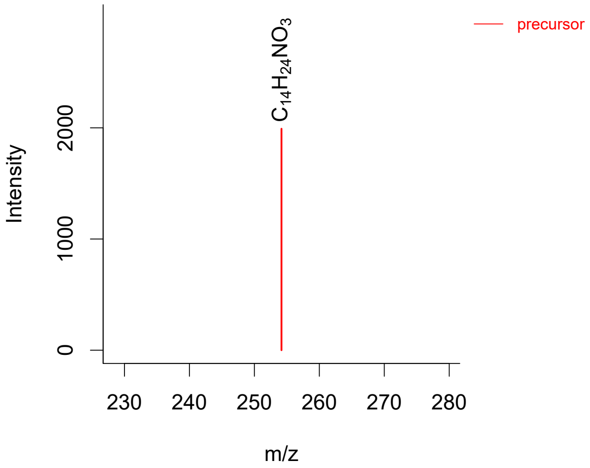

2.2.8.3 Candidate 'SuS-MET-M254-6'

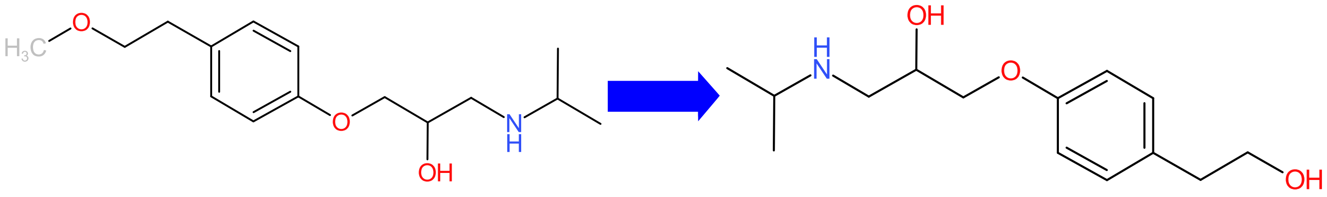

Formula	$C_{14}H_{23}NO_3$ (Δ -CH_2)
SMILES	CC(C)NCC(COC1=CC=C(C=C1)CCO)O
m/z error	-0.1 mDa
XLog P	1.1 (Δ -0.5)
Data source(s)	BTE, BTH, PC (162181), LIT (a, b, c)
In silico similarity	0.00 (formula), 0.00 (compound)
ID confidence level	5
Fit	$fit_{formula}$: 1.00<br>$fit_{compound}$: 1.00

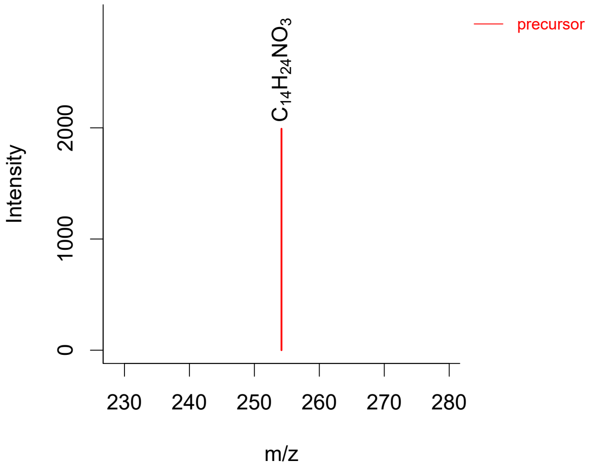

2.2.9 Feature ‘M268_R384_4267’

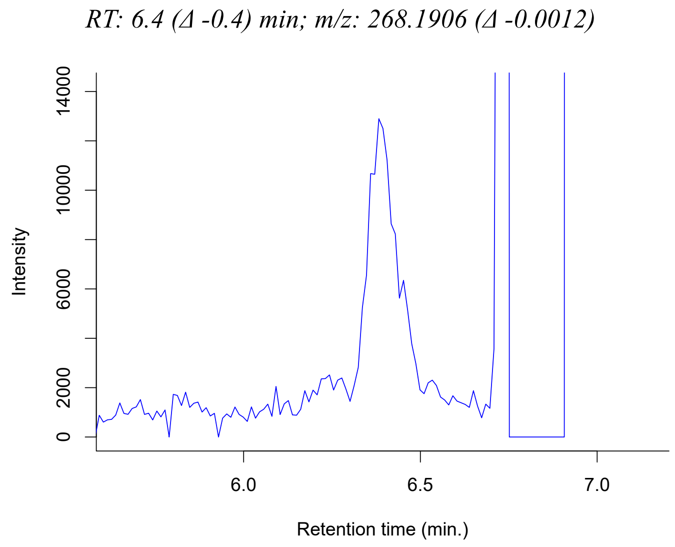

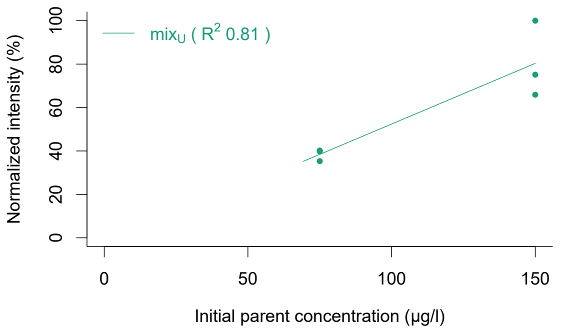

Condition	mix 25	mix 75	mix 150	RSQ	p	slope	single	dark
U		38%	80%	0.81	0.015	0.56%	45%	
UH								
UHN		22%	[3]				36%	

2.2.9.1 Candidate ‘SuS-MET-M268-1’

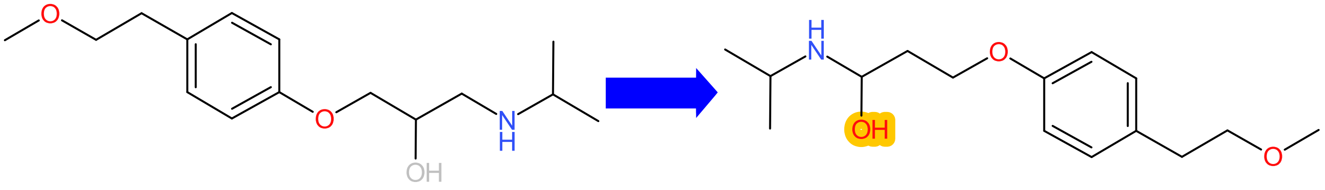

Formula	$C_{15}H_{25}NO_3$ (no difference)
SMILES	CC(C)NC(CCOC1=CC=C(C=C1)CCOC)O
m/z error	-0.2 mDa
XLog P	2.1 (Δ +0.5)
Data source(s)	BTE
In silico similarity	1.00 (formula), 1.00 (compound)
ID confidence level	3d
Fit	$fit_{formula}$: 1.00<br>$fit_{compound}$: 0.95

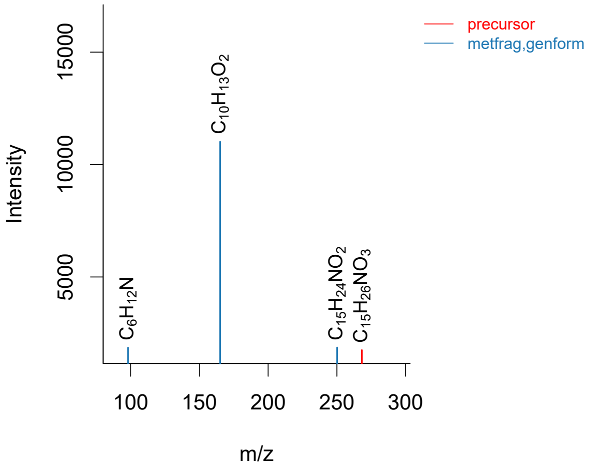

2.2.10 Feature 'M282_R397_5476'

RT: 6.6 (Δ -0.2) min; m/z: 282.1699 (Δ +13.9782)

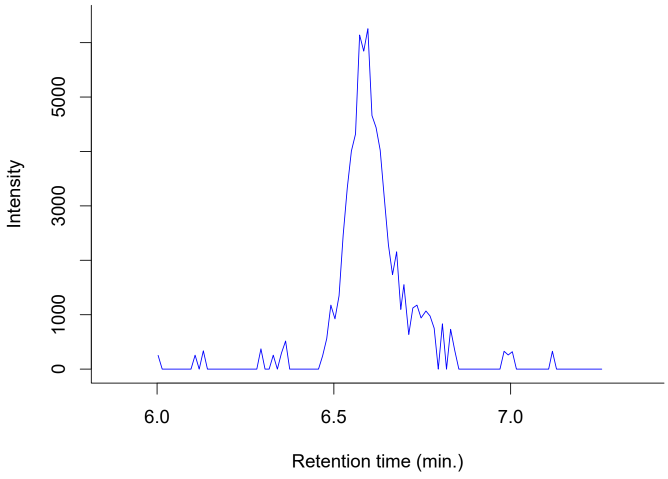

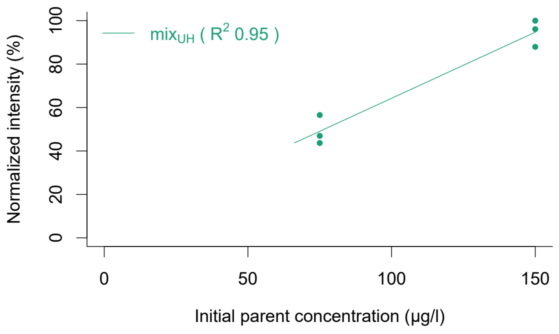

Condition	mix 25	mix 75	mix 150	RSQ	p	slope	single	dark
U			74%					
UH		49%	95%	0.95	0.001	0.61%	59%	
UHN			57%					

2.2.10.1 Candidate 'SuS-MET-M282-1'

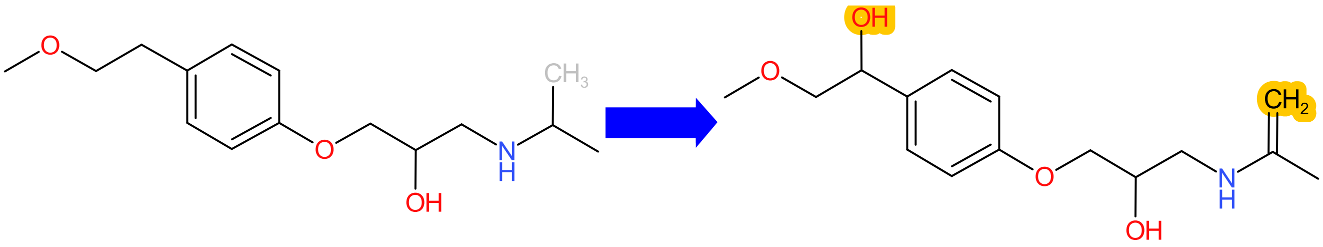

Formula	$C_{15}H_{23}NO_4$ (Δ $-H_2$ +O)
SMILES	C=C(C)NCC(COC1=CC=C(C=C1)C(COC)O)O
m/z error	-0.1 mDa
XLog P	0.9 (Δ -0.7)
Data source(s)	BTH
In silico similarity	0.00 (formula)
ID confidence level	5
Fit	$fit_{formula}$: 0.98<br>$fit_{compound}$: 0.95

No MS/MS annotations

2.2.10.2 Candidate 'SuS-MET-M282-2'

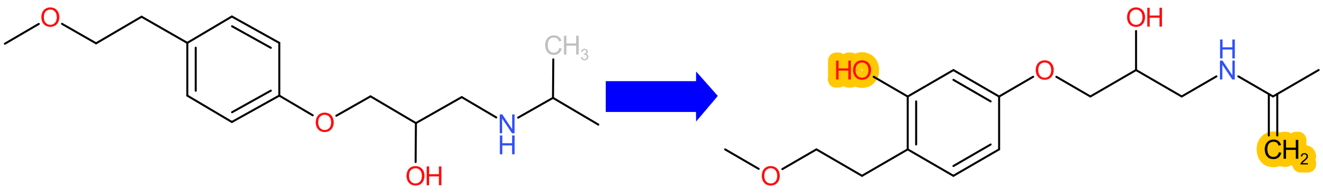

Formula	$C_{15}H_{23}NO_4$ (Δ -H_2 +O)
SMILES	C=C(C)NCC(COC1=CC(=C(C=C1)CCOC)O)O
m/z error	-0.1 mDa
XLog P	1.3 (Δ -0.3)
Data source(s)	BTH
In silico similarity	0.00 (formula)
ID confidence level	5
Fit	$fit_{formula}$: 0.98<br>$fit_{compound}$: 0.95

No MS/MS annotations

2.2.10.3 Candidate 'SuS-MET-M282-3'

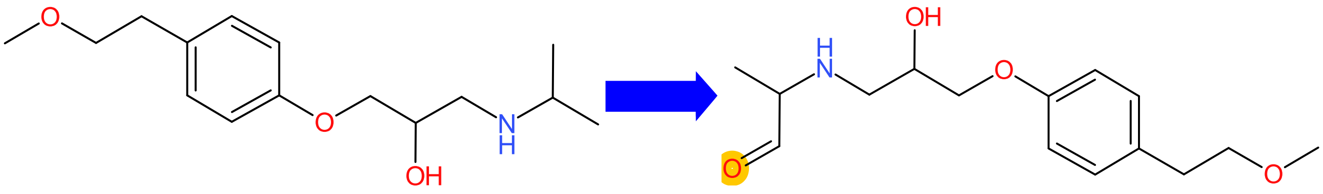

Formula	$C_{15}H_{23}NO_4$ (Δ -H_2 +O)
SMILES	C(C(C)NCC(COC1=CC=C(C=C1)CCOC)O)=O
m/z error	-0.1 mDa
XLog P	1.1 (Δ -0.6)
Data source(s)	BTH
In silico similarity	0.00 (formula)
ID confidence level	5
Fit	$fit_{formula}$: 0.98<br>$fit_{compound}$: 1.00

No MS/MS annotations

2.2.10.4 Candidate 'SuS-MET-M282-4'

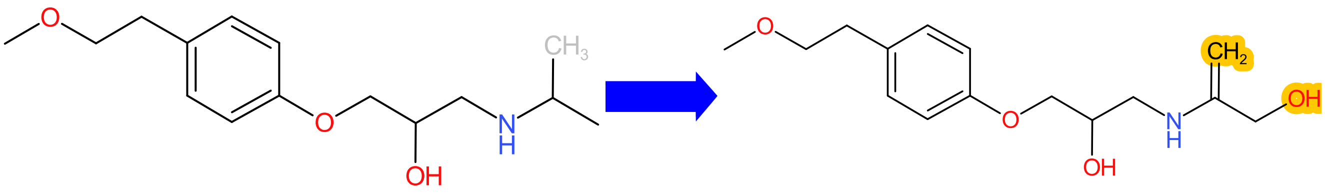

Formula	$C_{15}H_{23}NO_4$ (Δ -H_2 +O)
SMILES	C(C(=C)NCC(COC1=CC=C(C=C1)CCOC)O)O
m/z error	-0.1 mDa
XLog P	0.7 (Δ -1.0)
Data source(s)	BTH
In silico similarity	0.00 (formula)
ID confidence level	5
Fit	$fit_{formula}$: 0.98<br>$fit_{compound}$: 0.95

No MS/MS annotations

2.2.10.5 Candidate 'SuS-MET-M282-5'

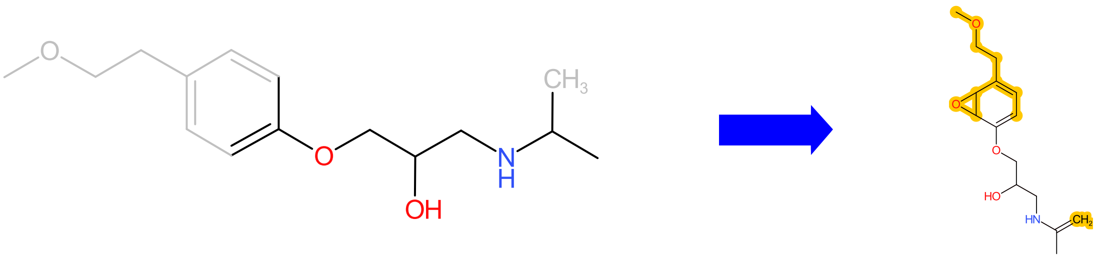

Formula	$C_{15}H_{23}NO_4$ (Δ -H_2 +O)
SMILES	C=C(C)NCC(COC=1C2C(C(=CC1)CCOC)O2)O
m/z error	-0.1 mDa
XLog P	-0.0 (Δ -1.6)
Data source(s)	BTH
In silico similarity	0.00 (formula)
ID confidence level	5
Fit	$fit_{formula}$: 0.98<br>$fit_{compound}$: 0.47

No MS/MS annotations

2.2.10.6 Candidate 'SuS-MET-M282-6'

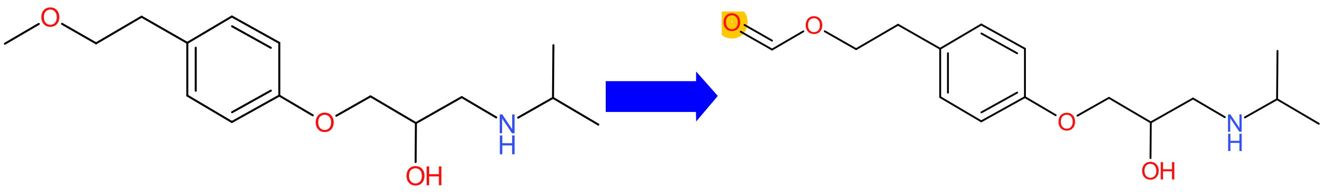

Formula	$C_{15}H_{23}NO_4$ (Δ -H_2 +O)
SMILES	CC(C)NCC(O)COc1ccc(CCOC=O)cc1
m/z error	-0.1 mDa
XLog P	1.6 (Δ -0.0)
Data source(s)	LIT (a, b)
In silico similarity	0.00 (formula)
ID confidence level	5
Fit	$fit_{formula}$: 0.98<br>$fit_{compound}$: 1.00

No MS/MS annotations

2.2.10.7 Candidate 'SuS-MET-M282-7'

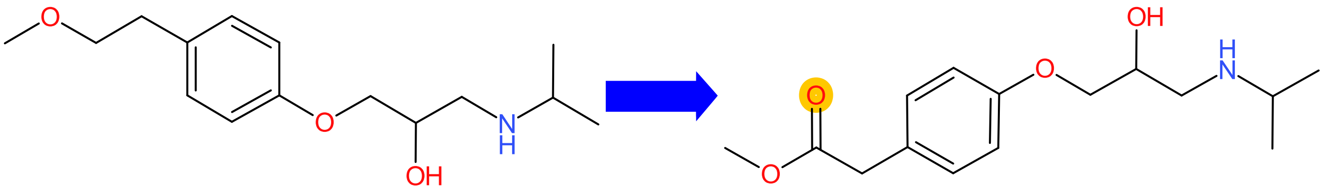

Formula	$C_{15}H_{23}NO_4$ (Δ -H_2 +O)
SMILES	COC(=O)Cc1ccc(OCC(O)CNC(C)C)cc1
m/z error	-0.1 mDa
XLog P	1.5 (Δ -0.1)
Data source(s)	LIT (a, b, c)
In silico similarity	0.00 (formula)
ID confidence level	5
Fit	$fit_{formula}$: 0.98<br>$fit_{compound}$: 1.00

No MS/MS annotations

2.2.10.8 Candidate 'SuS-MET-M282-8'

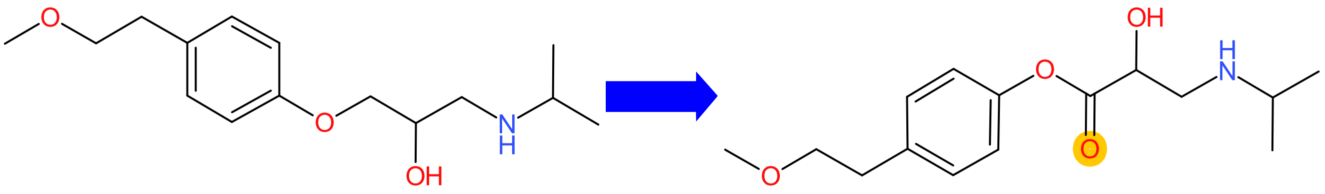

Formula	$C_{15}H_{23}NO_4$ (Δ -H_2 +O)
SMILES	COCCc1ccc(OC(=O)C(O)CNC(C)C)cc1
m/z error	-0.1 mDa
XLog P	1.2 (Δ -0.4)
Data source(s)	LIT (a)
In silico similarity	0.00 (formula)
ID confidence level	5
Fit	$fit_{formula}$: 0.98<br>$fit_{compound}$: 1.00

No MS/MS annotations

2.2.10.9 Candidate 'SuS-MET-M282-9'

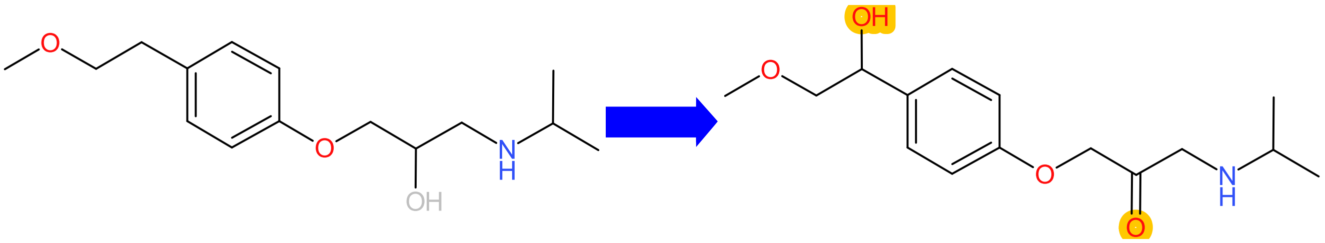

Formula	$C_{15}H_{23}NO_4$ (Δ -H_2 +O)
SMILES	CC(C)NCC(COC1=CC=C(C=C1)C(COC)O)=O
m/z error	-0.1 mDa
XLog P	0.9 (Δ -0.8)
Data source(s)	BTH
In silico similarity	0.00 (formula)
ID confidence level	5
Fit	$fit_{formula}$: 0.98<br>$fit_{compound}$: 0.95

No MS/MS annotations

2.2.10.10 Candidate 'SuS-MET-M282-10'

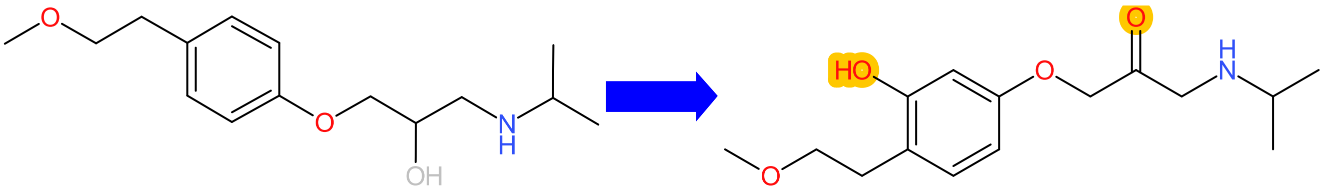

Formula	$C_{15}H_{23}NO_4$ (Δ -H_2 +O)
SMILES	CC(C)NCC(COC1=CC(=C(C=C1)CCOC)O)=O
m/z error	-0.1 mDa
XLog P	1.2 (Δ -0.4)
Data source(s)	BTH
In silico similarity	0.00 (formula)
ID confidence level	5
Fit	$fit_{formula}$: 0.98<br>$fit_{compound}$: 0.95

No MS/MS annotations

2.2.10.11 Candidate 'SuS-MET-M282-11'

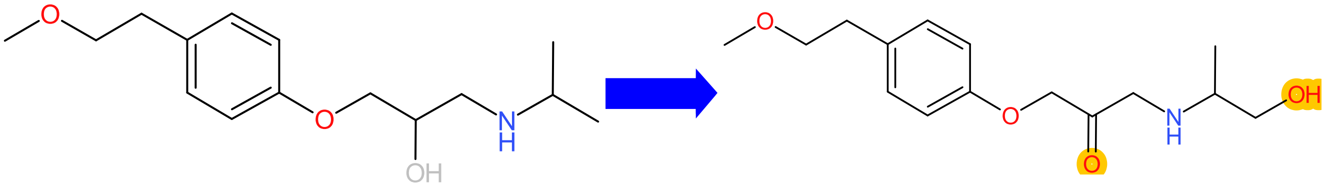

Formula	$C_{15}H_{23}NO_4$ (Δ -H_2 +O)
SMILES	C(C(C)NCC(COC1=CC=C(C=C1)CCOC)=O)O
m/z error	-0.1 mDa
XLog P	0.5 (Δ -1.1)
Data source(s)	BTH
In silico similarity	0.00 (formula)
ID confidence level	5
Fit	$fit_{formula}$: 0.98<br>$fit_{compound}$: 0.95

No MS/MS annotations

2.2.10.12 Candidate 'SuS-MET-M282-12'

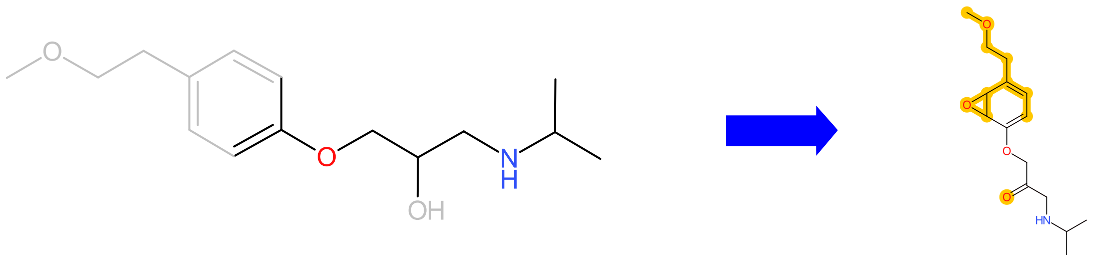

Formula	$C_{15}H_{23}NO_4$ (Δ -H_2 +O)
SMILES	CC(C)NCC(COC=1C2C(C(=CC1)CCOC)O2)=O
m/z error	-0.1 mDa
XLog P	-0.1 (Δ -1.7)
Data source(s)	BTH
In silico similarity	0.00 (formula)
ID confidence level	5
Fit	$fit_{formula}$: 0.98<br>$fit_{compound}$: 0.47

No MS/MS annotations

2.2.10.13 Candidate 'SuS-MET-M282-13'

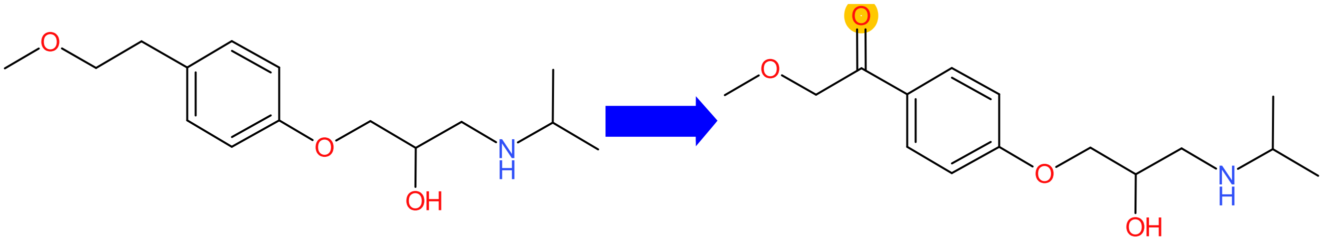

Formula	$C_{15}H_{23}NO_4$ (Δ -H_2 +O)
SMILES	CC(C)NCC(COC1=CC=C(C=C1)C(COC)=O)O
m/z error	-0.1 mDa
XLog P	1.1 (Δ -0.5)
Data source(s)	BTH, LIT (a, b, c)
In silico similarity	0.00 (formula)
ID confidence level	5
Fit	$fit_{formula}$: 0.98<br>$fit_{compound}$: 1.00

No MS/MS annotations

2.2.11 Feature ‘M284_R387_3283’

RT: 6.4 (Δ -0.4) min; m/z: 284.1851 (Δ +15.9934)

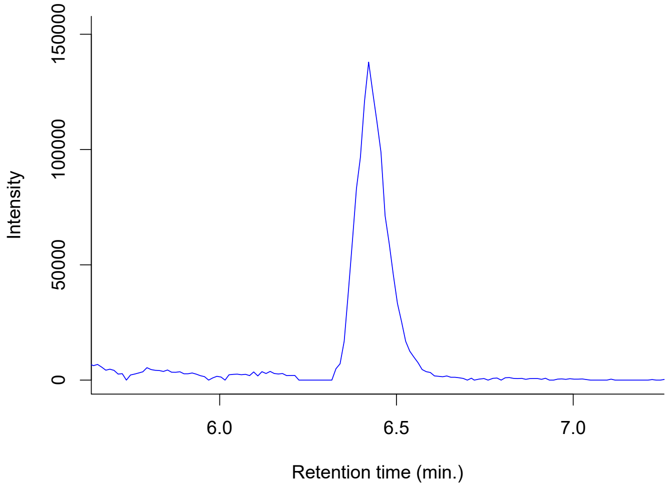

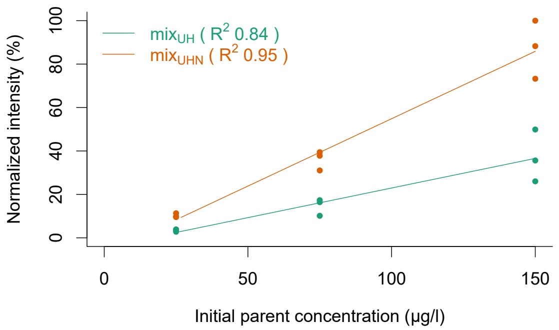

Condition	mix 25	mix 75	mix 150	RSQ	p	slope	single	dark
U			6%				2%	
UH	3%	15%	37%	0.84	0.000	0.27%	12%	
UHN	10%	36%	87%	0.95	0.000	0.62%	49%	

2.2.11.1 Candidate ‘SuS-MET-M284-1’

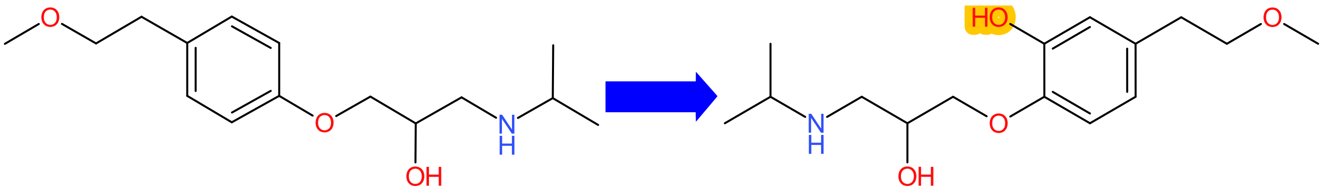

Formula	$C_{15}H_{25}NO_4$ (Δ +O)
SMILES	COCCc1ccc(OCC(O)CNC(C)C)c(O)c1
m/z error	-0.5 mDa
XLog P	1.0 (Δ -0.7)
Data source(s)	LIT (a, b)
In silico similarity	0.94 (formula), 0.92 (compound)
ID confidence level	3d
Fit	$fit_{formula}$: 1.00<br>$fit_{compound}$: 1.00
Other matches	M284_R326_2405

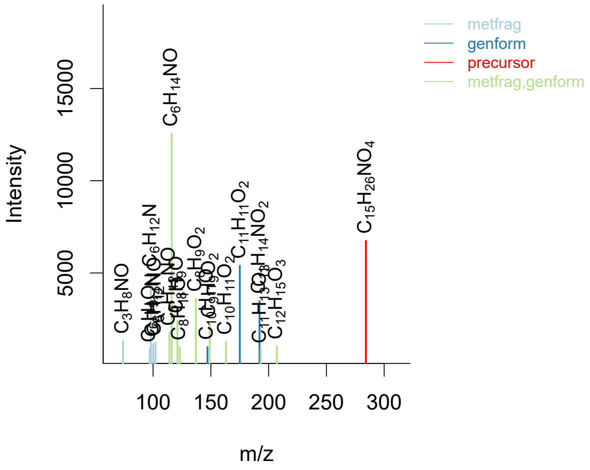

2.2.11.2 Candidate 'SuS-MET-M284-2'

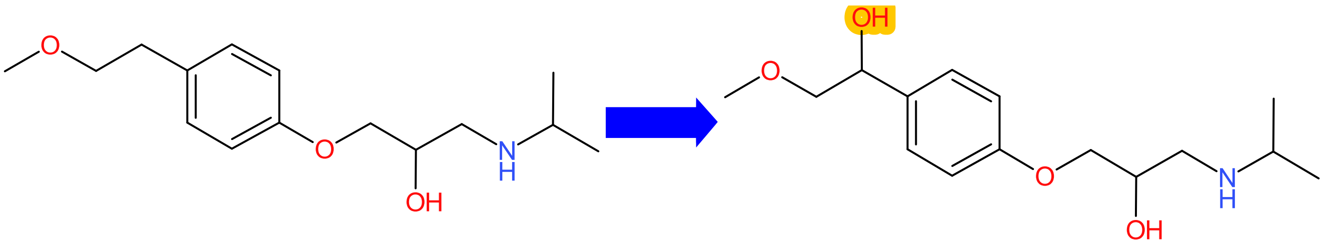

Formula	$C_{15}H_{25}NO_4$ (Δ +O)
SMILES	CC(C)NCC(COC1=CC=C(C=C1)C(COC)O)O
m/z error	-0.5 mDa
XLog P	0.9 (Δ -0.8)
Data source(s)	BTH, PC (114962), LIT (a, b, c)
In silico similarity	0.94 (formula), 0.94 (compound)
ID confidence level	3d
Fit	$fit_{formula}$: 1.00<br>$fit_{compound}$: 1.00
Other matches	M284_R326_2405

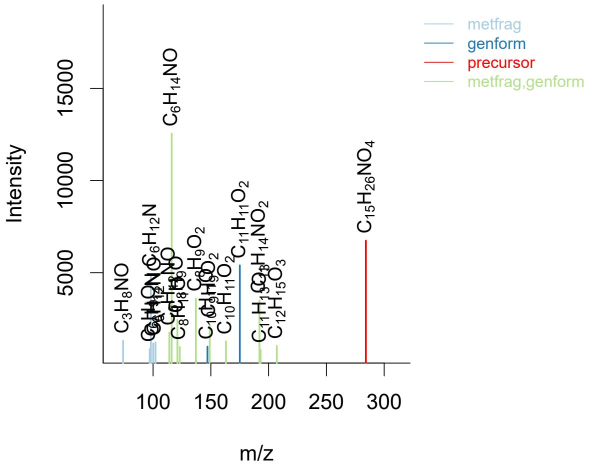

2.2.11.3 Candidate 'SuS-MET-M284-3'

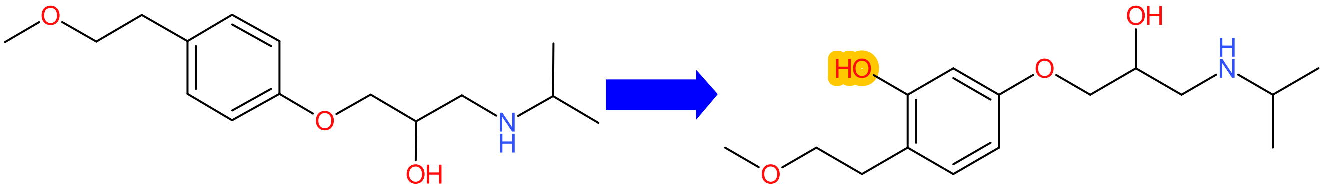

Formula	$C_{15}H_{25}NO_4$ (Δ +O)
SMILES	CC(C)NCC(COC1=CC(=C(C=C1)CCOC)O)O
m/z error	-0.5 mDa
XLog P	1.2 (Δ -0.4)
Data source(s)	BTH, LIT (a, b)
In silico similarity	0.94 (formula), 0.92 (compound)
ID confidence level	3d
Fit	$fit_{formula}$: 1.00<br>$fit_{compound}$: 1.00
Other matches	M284_R326_2405

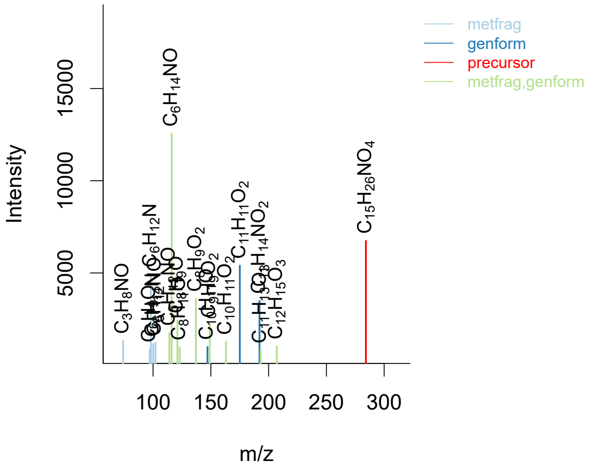

2.2.11.4 Candidate 'SuS-MET-M284-4'

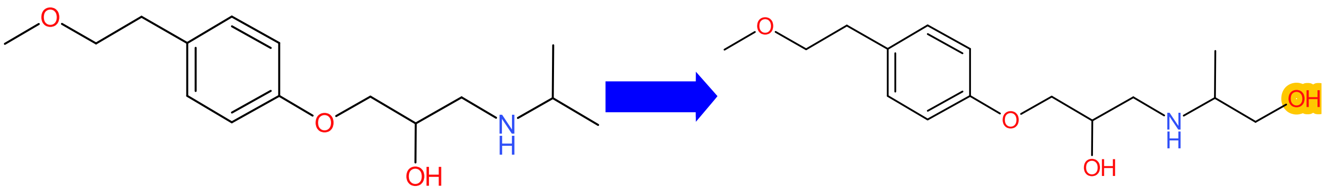

Formula	$C_{15}H_{25}NO_4$ (Δ +O)
SMILES	C(C(C)NCC(COC1=CC=C(C=C1)CCOC)O)O
m/z error	-0.5 mDa
XLog P	0.5 (Δ -1.1)
Data source(s)	BTH
In silico similarity	0.94 (formula), 0.88 (compound)
ID confidence level	3d
Fit	$fit_{formula}$: 1.00<br>$fit_{compound}$: 1.00
Other matches	M284_R326_2405

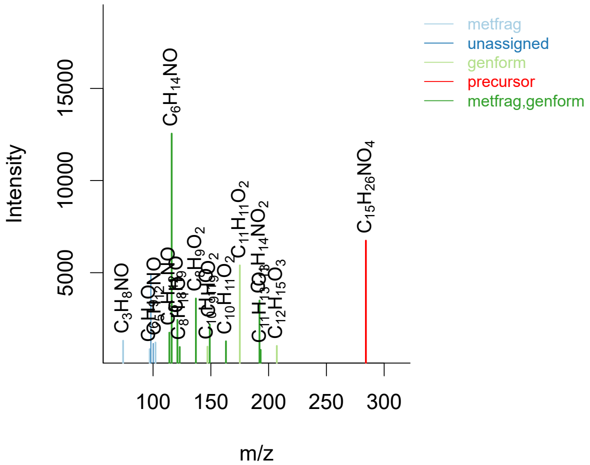

2.2.11.5 Candidate 'SuS-MET-M284-5'

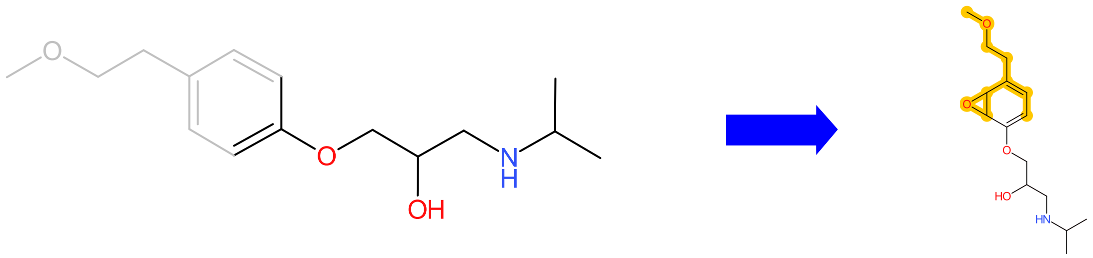

Formula	$C_{15}H_{25}NO_4$ (Δ +O)
SMILES	CC(C)NCC(COC=1C2C(C(=CC1)CCOC)O2)O
m/z error	-0.5 mDa
XLog P	-0.1 (Δ -1.7)
Data source(s)	BTH
In silico similarity	0.94 (formula), 0.92 (compound)
ID confidence level	3d
Fit	$fit_{formula}$: 1.00<br>$fit_{compound}$: 0.53
Other matches	M284_R326_2405

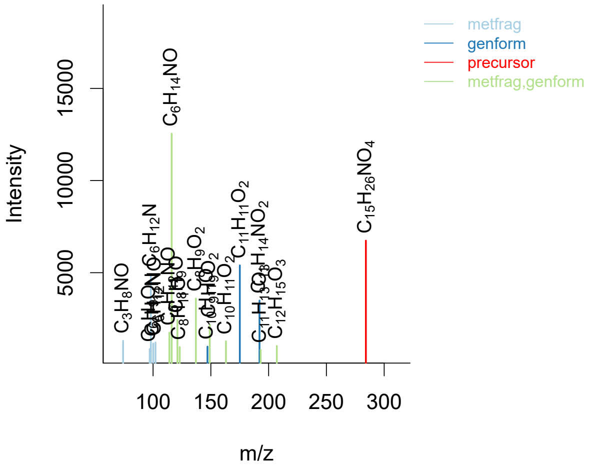

2.2.12 Feature ‘M284_R326_2405’

RT: 5.4 (Δ -1.4) min; m/z: 284.1856 (Δ +15.9938)

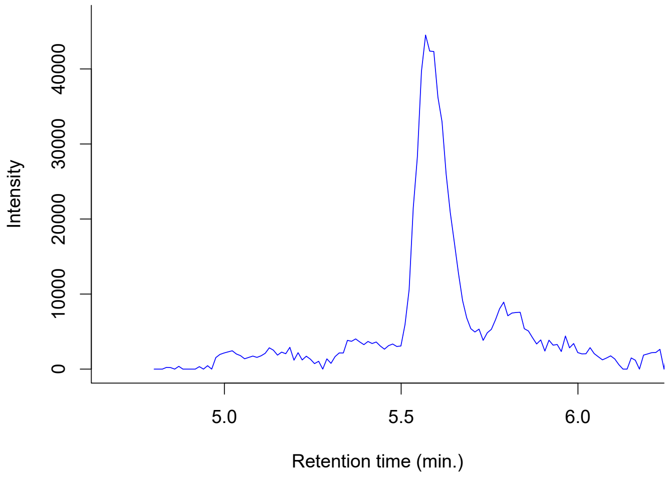

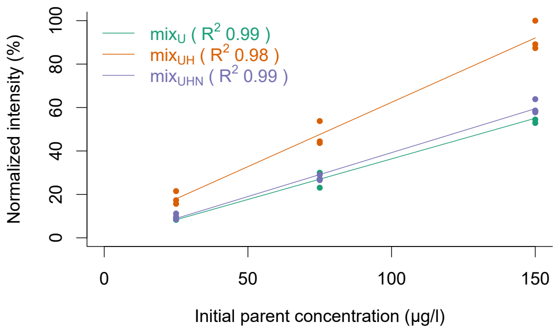

Condition	mix 25	mix 75	mix 150	RSQ	p	slope	single	dark
U	9%	27%	55%	0.99	0.000	0.37%	21%	7%
UH	18%	47%	92%	0.98	0.000	0.59%	1	3
UHN	10%	28%	60%	0.99	0.000	0.4%	34%	6%

2.2.12.1 Candidate ‘SuS-MET-M284-1’

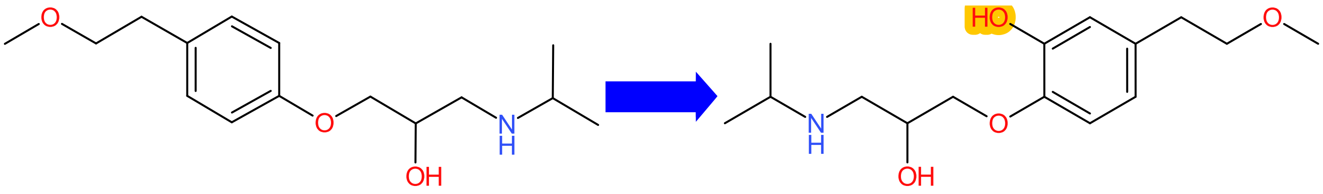

Formula	$C_{15}H_{25}NO_4$ (Δ +O)
SMILES	COCCc1ccc(OCC(O)CNC(C)C)c(O)c1
m/z error	-0.1 mDa
XLog P	1.0 (Δ -0.7)
Data source(s)	LIT (a, b)
In silico similarity	0.90 (formula), 0.98 (compound)
ID confidence level	3d
Fit	$fit_{formula}$: 1.00<br>$fit_{compound}$: 1.00
Other matches	M284_R387_3283

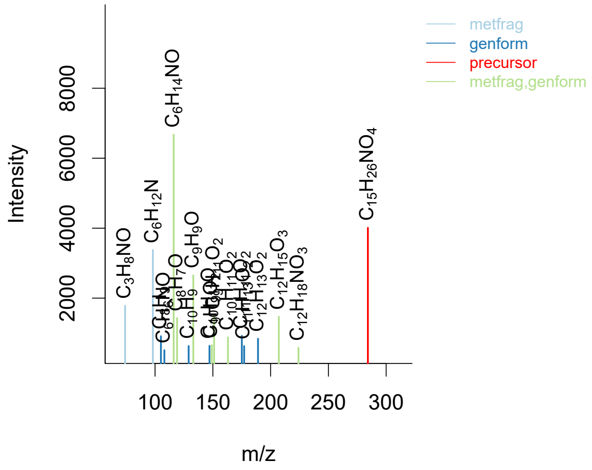

2.2.12.2 Candidate 'SuS-MET-M284-2'

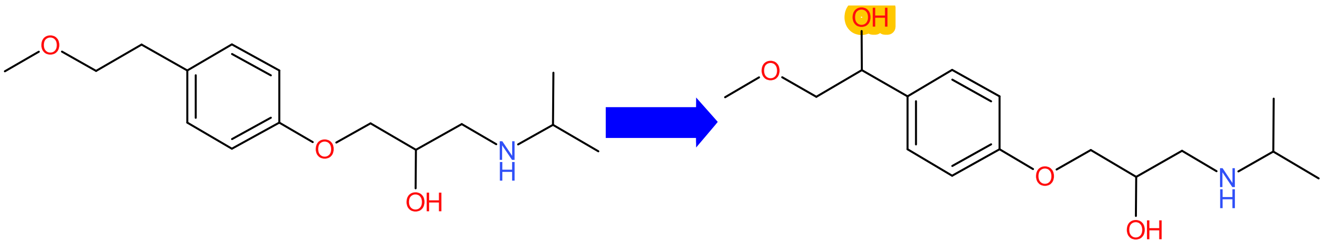

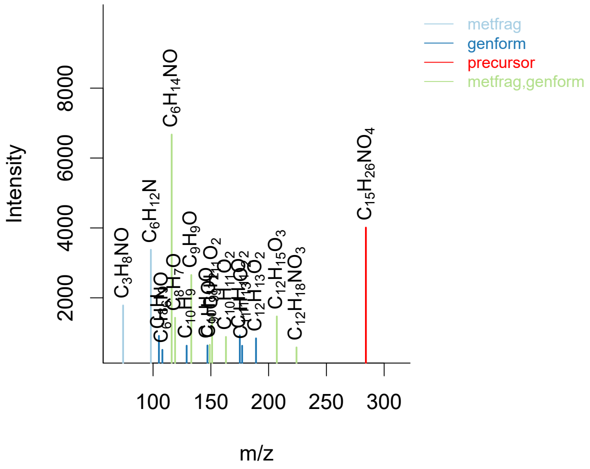

Formula	$C_{15}H_{25}NO_4$ (Δ +O)
SMILES	CC(C)NCC(COC1=CC=C(C=C1)C(COC)O)O
m/z error	-0.1 mDa
XLog P	0.9 (Δ -0.8)
Data source(s)	BTH, PC (114962), LIT (a, b, c)
In silico similarity	0.90 (formula), 0.98 (compound)
ID confidence level	3d
Fit	$fit_{formula}$: 1.00<br>$fit_{compound}$: 1.00
Other matches	M284_R387_3283

2.2.12.3 Candidate 'SuS-MET-M284-3'

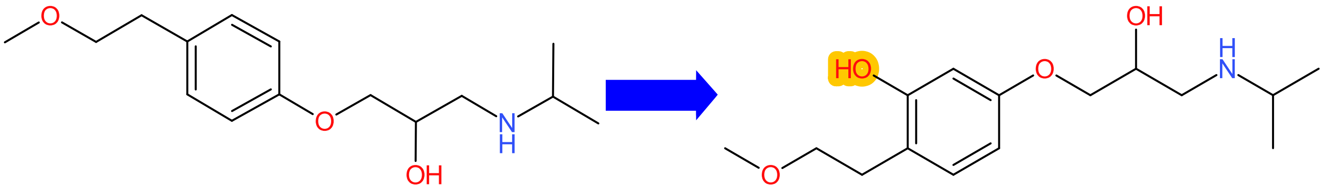

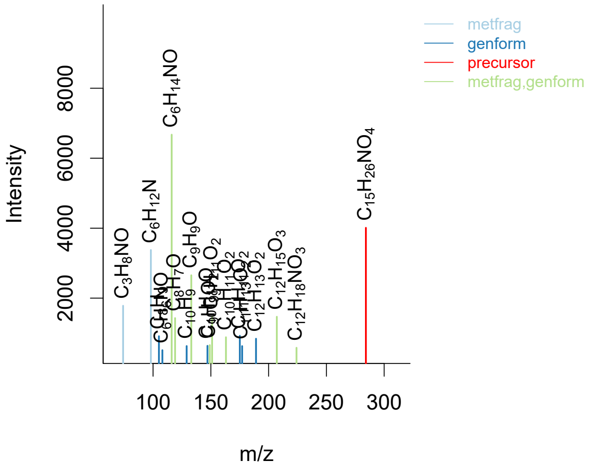

Formula	$C_{15}H_{25}NO_4$ (Δ +O)
SMILES	CC(C)NCC(COC1=CC(=C(C=C1)CCOC)O)O
m/z error	-0.1 mDa
XLog P	1.2 (Δ -0.4)
Data source(s)	BTH, LIT (a, b)
In silico similarity	0.90 (formula), 0.98 (compound)
ID confidence level	3d
Fit	$fit_{formula}$: 1.00<br>$fit_{compound}$: 1.00
Other matches	M284_R387_3283

2.2.12.4 Candidate ‘SuS-MET-M284-4’

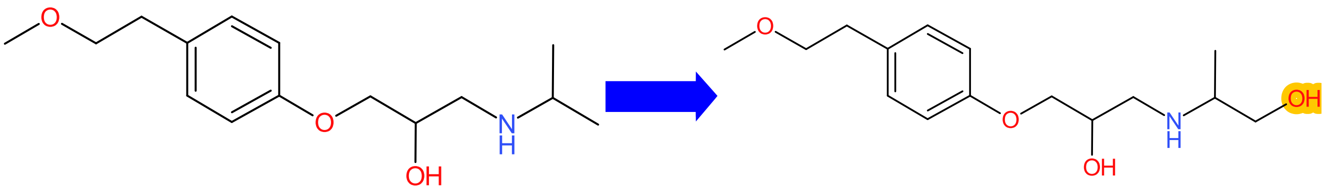

Formula	$C_{15}H_{25}NO_4$ (Δ +O)
SMILES	C(C(C)NCC(COC1=CC=C(C=C1)CCOC)O)O
m/z error	-0.1 mDa
XLog P	0.5 (Δ -1.1)
Data source(s)	BTH
In silico similarity	0.90 (formula), 0.89 (compound)
ID confidence level	3d
Fit	$fit_{formula}$: 1.00<br>$fit_{compound}$: 1.00
Other matches	M284_R387_3283

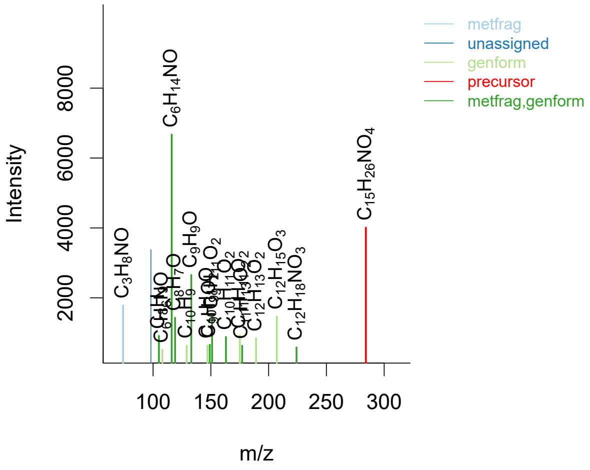

2.2.12.5 Candidate ‘SuS-MET-M284-5’

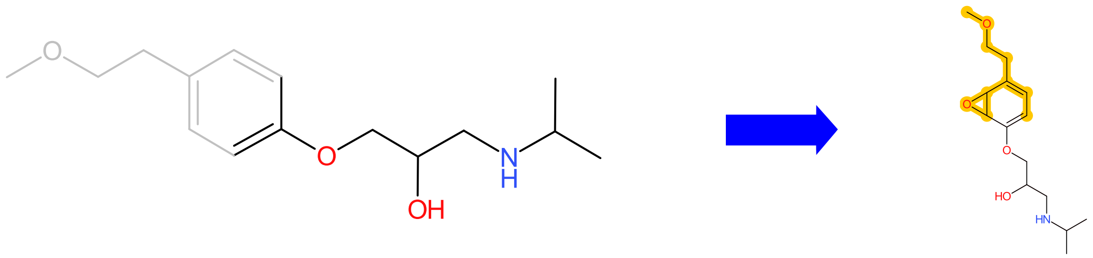

Formula	$C_{15}H_{25}NO_4$ (Δ +O)
SMILES	CC(C)NCC(COC=1C2C(C(=CC1)CCOC)O2)O
m/z error	-0.1 mDa
XLog P	-0.1 (Δ -1.7)
Data source(s)	BTH
In silico similarity	0.90 (formula), 0.98 (compound)
ID confidence level	3d
Fit	$fit_{formula}$: 1.00<br>$fit_{compound}$: 0.53
Other matches	M284_R387_3283

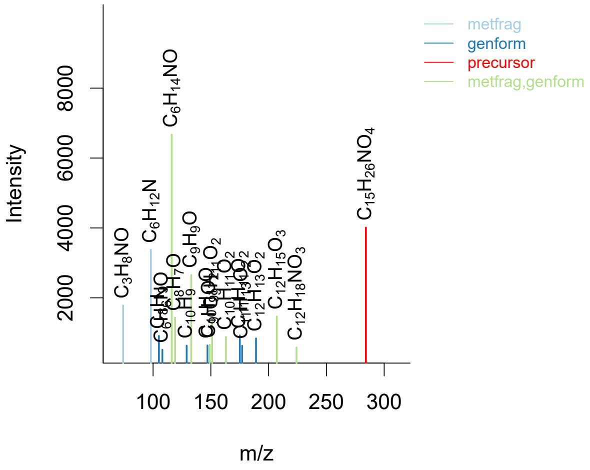

2.3 Parent ‘sulfamethoxazole’

2.3.1 Feature ‘M94_R231_2478’

RT: 3.9 (Δ -3.6) min; m/z: 94.0645 (Δ -159.9952)

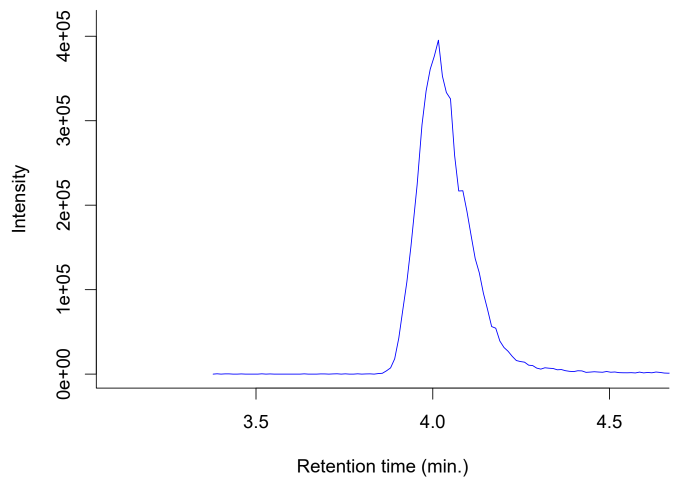

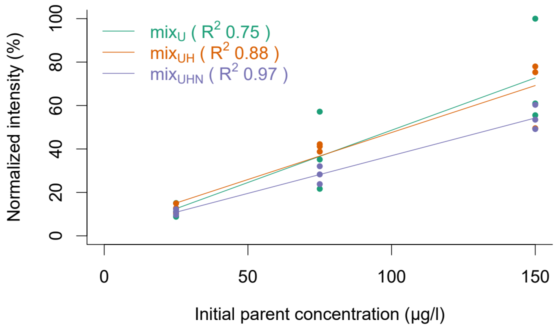

Condition	mix 25	mix 75	mix 150	RSQ	p	slope	single	dark
U	12%	38%	72%	0.75	0.003	0.48%	4%	
UH	13%	41%	68%	0.88	0.000	0.43%	3%	
UHN	11%	28%	54%	0.97	0.000	0.35%	6%	

2.3.1.1 Candidate ‘SuS-SMX-M94-1’

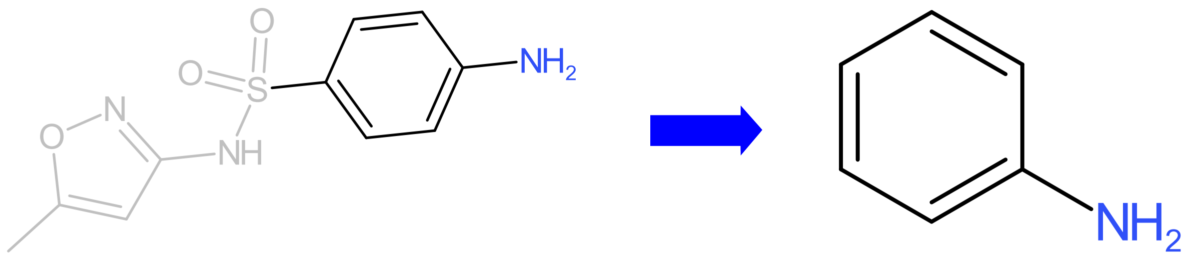

Formula	C_6H_7N (Δ -$C_4H_4N_2O_3S$)
SMILES	Nc1ccccc1
m/z error	-0.7 mDa
XLog P	1.2 (Δ +0.0)
Data source(s)	CTS, LIT (a)
In silico similarity	NA (formula), 1.00 (compound)
ID confidence level	1
Fit	$fit_{formula}$: 1.00<br>$fit_{compound}$: 1.00

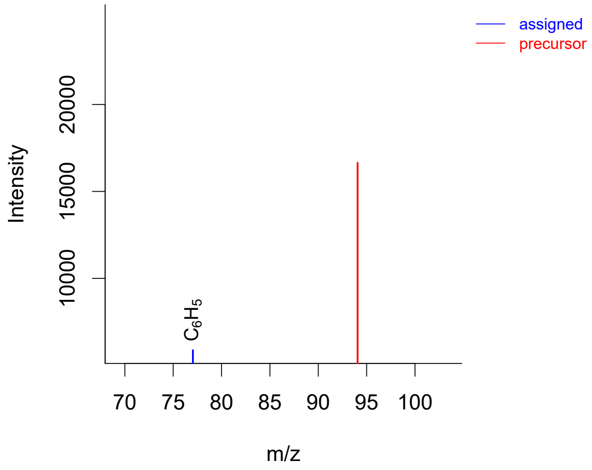

2.3.2 Feature 'M99_R289_3167'

RT: 4.8 (Δ -2.7) min; m/z: 99.0546 (Δ -155.0051)

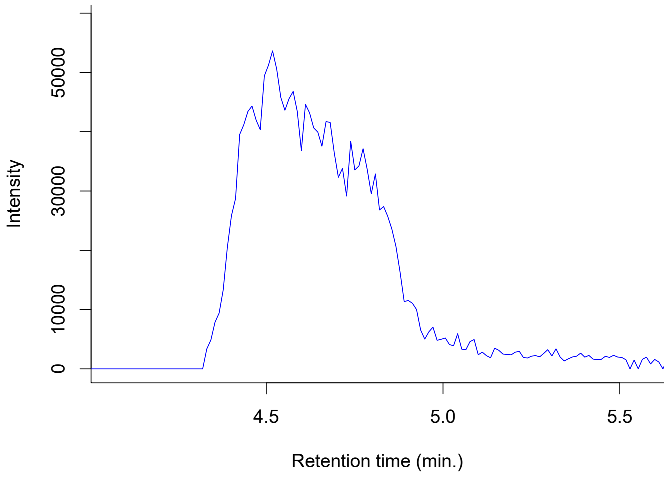

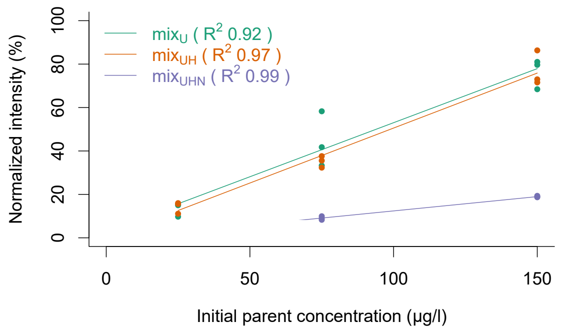

Condition	mix 25	mix 75	mix 150	RSQ	p	slope	single	dark
U	13%	44%	76%	0.92	0.000	0.5%	98%	
UH	14%	35%	77%	0.97	0.000	0.51%	59%	
UHN		9%	19%	0.99	0.000	0.13%		

2.3.2.1 Candidate 'SuS-SMX-M99-1'

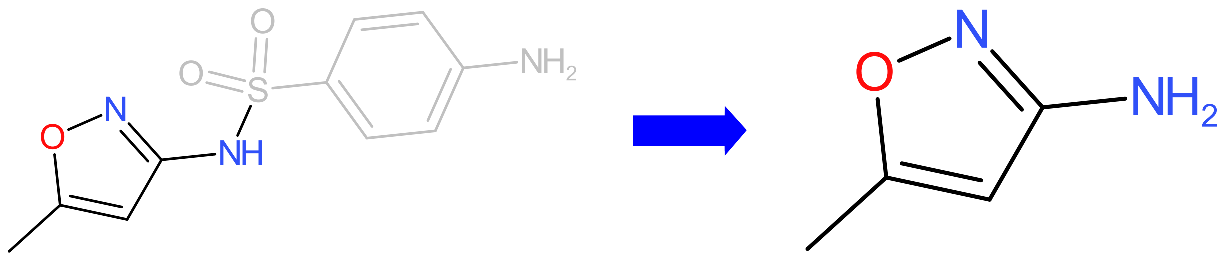

Formula	$C_4H_6N_2O$ (Δ $-C_6H_5NO_2S$)
SMILES	Cc1cc(N)no1
m/z error	-0.7 mDa
XLog P	0.5 (Δ -0.7)
Data source(s)	CTS, BTE, LIT (a, b, c, d, e, f, g, h)
In silico similarity	NA (formula), 0.00 (compound)
ID confidence level	1
Fit	$fit_{formula}$: 1.00<br>$fit_{compound}$: 1.00
Other matches	M99_R136_3553

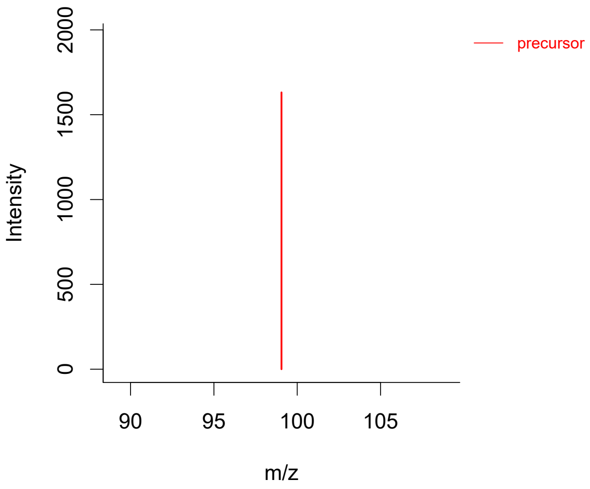

2.3.3 Feature ‘M99_R136_3553’

RT: 2.3 (Δ -5.2) min; m/z: 99.0547 (Δ -155.0050)

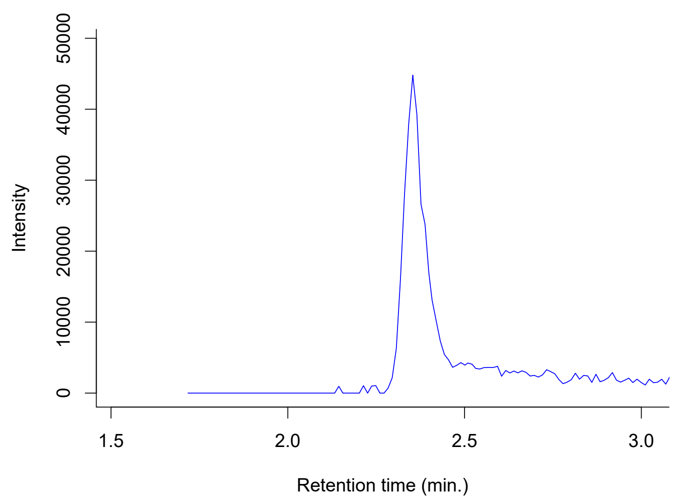

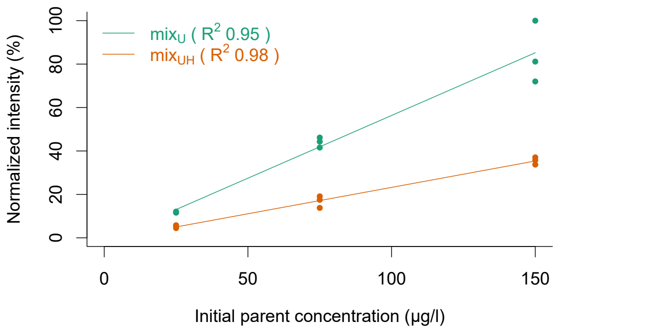

Condition	mix 25	mix 75	mix 150	RSQ	p	slope	single	dark
U	12%	44%	84%	0.95	0.000	0.58%	28%	
UH	5%	17%	36%	0.98	0.000	0.24%	24%	
UHN			12%					

2.3.3.1 Candidate ‘SuS-SMX-M99-1’ (*DISPROVED*)

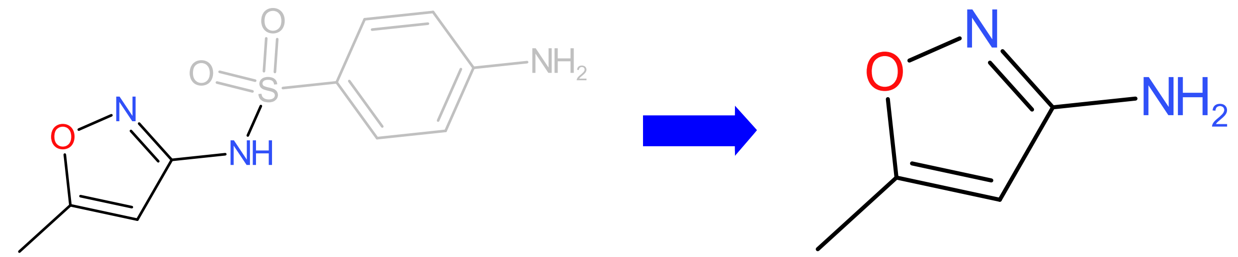

Formula	$C_4H_6N_2O$ (Δ -$C_6H_5NO_2S$)
SMILES	Cc1cc(N)no1
m/z error	-0.6 mDa
XLog P	0.5 (Δ -0.7)
Data source(s)	CTS, BTE, LIT (a, b, c, d, e, f, g, h)
In silico similarity	NA (formula), 0.00 (compound)
ID confidence level	**disproved by standard**
Fit	$fit_{formula}$: 1.00<br>$fit_{compound}$: 1.00
Other matches	M99_R289_3167

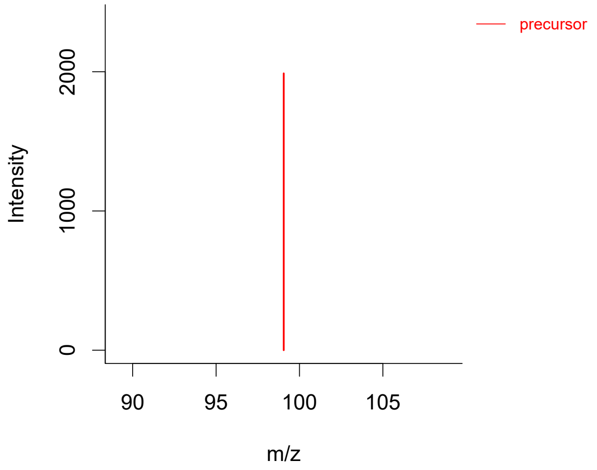

2.3.4 Feature ‘M110_R126_3769’

RT: 2.1 (Δ -5.4) min; m/z: 110.0600 (Δ -143.9997)

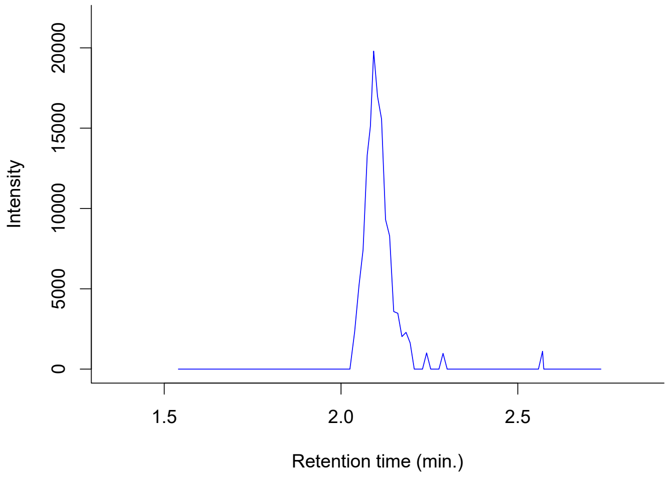

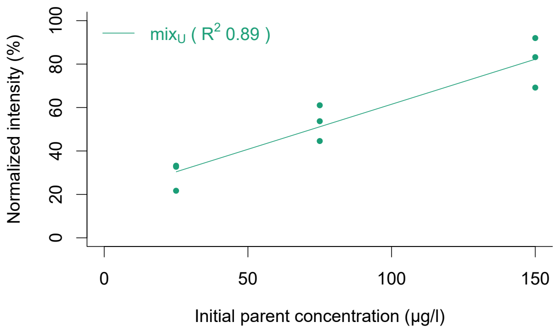

Condition	mix 25	mix 75	mix 150	RSQ	p	slope	single	dark
U	29%	53%	81%	0.89	0.000	0.41%		
UH		28%						
UHN	36%	97%	[3]	0.99	0.000	1.21%		

2.3.4.1 Candidate ‘SuS-SMX-M110-1’ (*DISPROVED*)

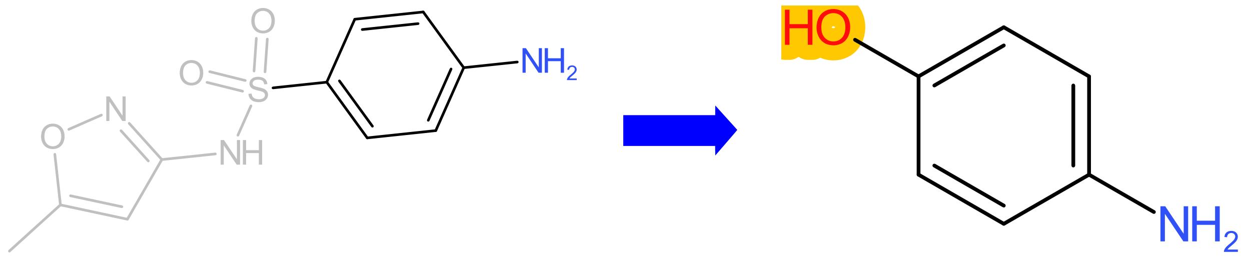

Formula	C_6H_7NO (Δ $-C_4H_4N_2O_2S$)
SMILES	C1(=CC=C(C=C1)N)O
m/z error	-0.1 mDa
XLog P	0.8 (Δ -0.4)
Data source(s)	BTE
In silico similarity	0.00 (formula)
ID confidence level	**disproved by standard**
Fit	$fit_{formula}$: 1.00<br>$fit_{compound}$: 0.88
Other matches	M110_R177_3768

No MS/MS annotations

2.3.5 Feature ‘M110_R177_3768’

RT: 3.0 (Δ -4.5) min; m/z: 110.0600 (Δ -143.9997)

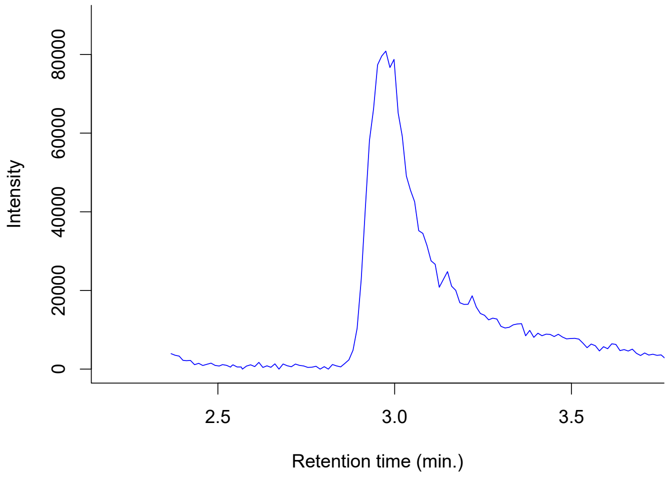

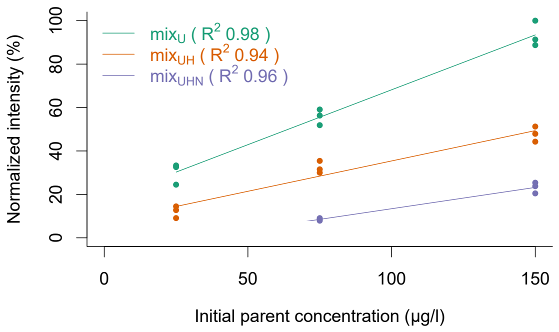

Condition	mix 25	mix 75	mix 150	RSQ	p	slope	single	dark
U	30%	56%	93%	0.98	0.000	0.51%		
UH	12%	32%	48%	0.94	0.000	0.28%		
UHN		8%	23%	0.96	0.001	0.2%		

2.3.5.1 Candidate ‘SuS-SMX-M110-1’ (*DISPROVED*)

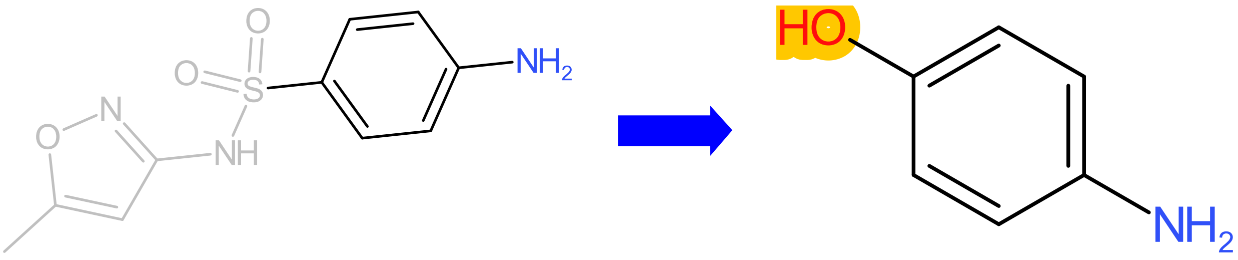

Formula	C_6H_7NO (Δ $-C_4H_4N_2O_2S$)
SMILES	C1(=CC=C(C=C1)N)O
m/z error	-0.0 mDa
XLog P	0.8 (Δ -0.4)
Data source(s)	BTE
In silico similarity	0.00 (formula), 1.00 (compound)
ID confidence level	**disproved by standard**
Fit	$fit_{formula}$: 1.00<br>$fit_{compound}$: 0.88
Other matches	M110_R126_3769

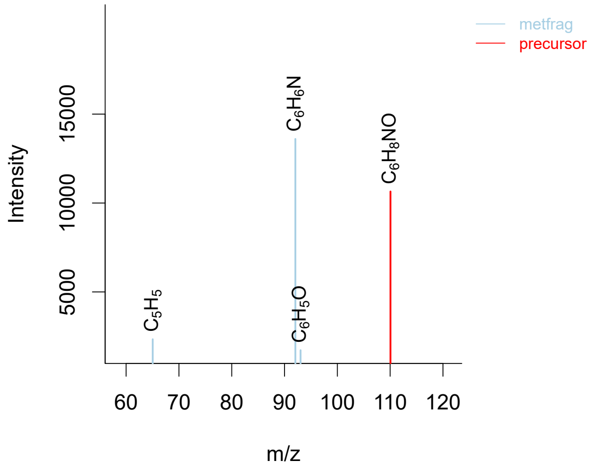

2.3.6 Feature ‘M142_R157_5040’

RT: 2.6 (Δ -4.9) min; m/z: 142.0498 (Δ -112.0099)

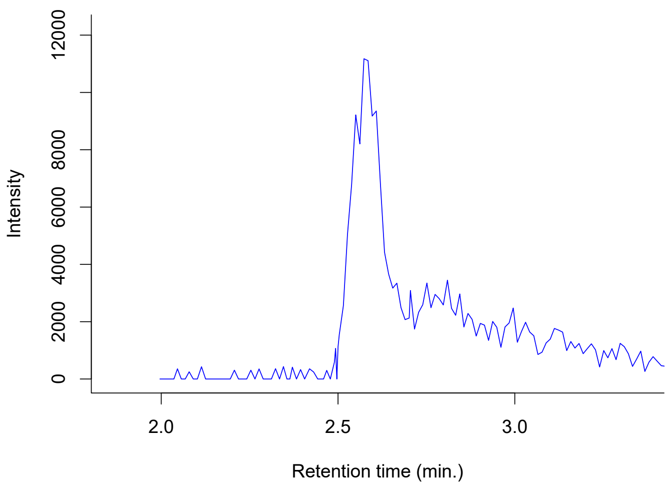

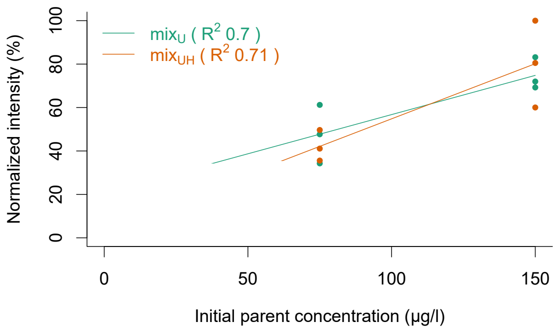

Condition	mix 25	mix 75	mix 150	RSQ	p	slope	single	dark
U		48%	75%	0.70	0.038	0.36%		
UH		42%	80%	0.71	0.036	0.51%		
UHN								

2.3.6.1 Candidate ‘SuS-SMX-M142-1’

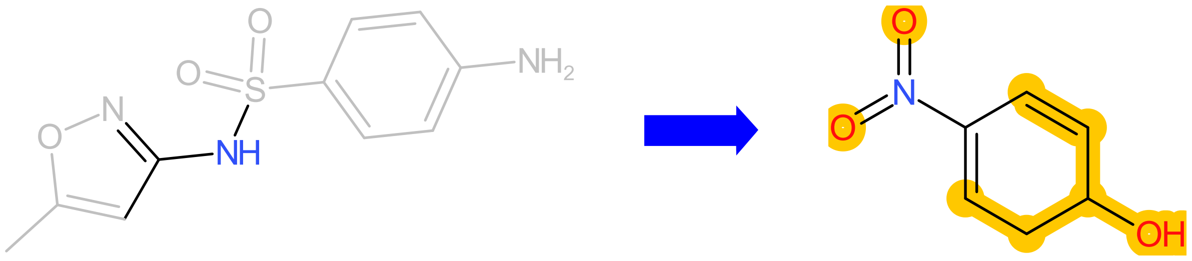

Formula	$C_6H_7NO_3$ (Δ $-C_4H_4N_2S$)
SMILES	O=N(=O)C1=CCC(O)C=C1
m/z error	-0.1 mDa
XLog P	0.6 (Δ -0.5)
Data source(s)	LIT (a)
In silico similarity	0.94 (formula), 0.94 (compound)
ID confidence level	3d
Fit	$fit_{formula}$: 1.00<br>$fit_{compound}$: 0.20

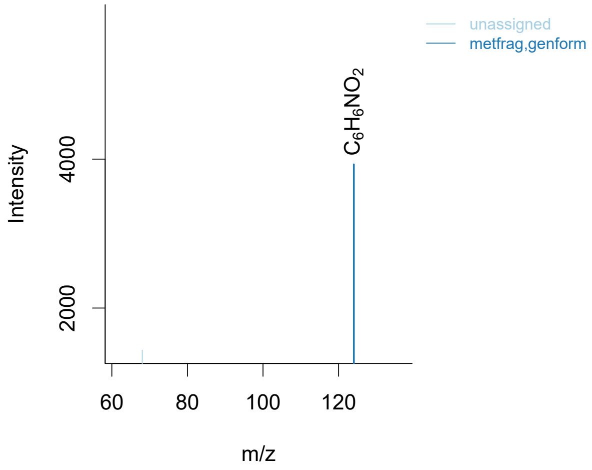

2.3.6.2 Candidate 'SuS-SMX-M142-2'

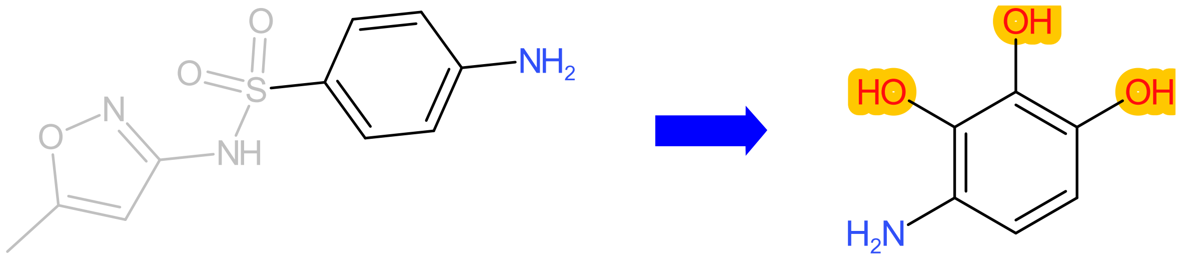

Formula	$C_6H_7NO_3$ (Δ -$C_4H_4N_2S$)
SMILES	C1(=C(C(=C(C=C1)N)O)O)O
m/z error	-0.1 mDa
XLog P	1.0 (Δ -0.2)
Data source(s)	BTE
In silico similarity	0.94 (formula), 1.00 (compound)
ID confidence level	3d
Fit	$fit_{formula}$: 1.00<br>$fit_{compound}$: 0.70

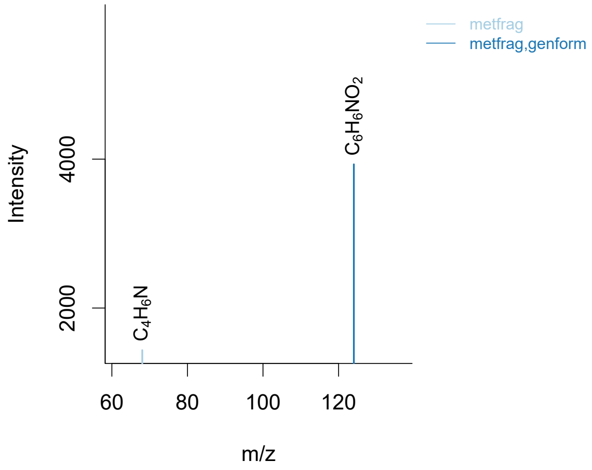

2.3.6.3 Candidate 'SuS-SMX-M142-3'

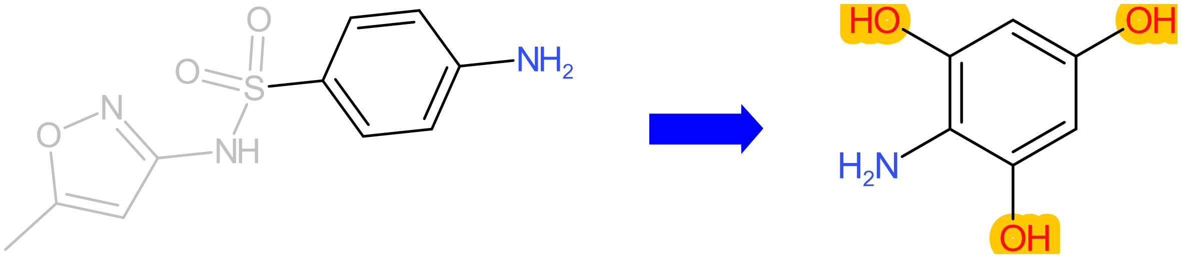

Formula	$C_6H_7NO_3$ (Δ -$C_4H_4N_2S$)
SMILES	C1(=CC(=C(C(=C1)O)N)O)O
m/z error	-0.1 mDa
XLog P	0.8 (Δ -0.3)
Data source(s)	BTE
In silico similarity	0.94 (formula), 0.94 (compound)
ID confidence level	3d
Fit	$fit_{formula}$: 1.00<br>$fit_{compound}$: 0.70

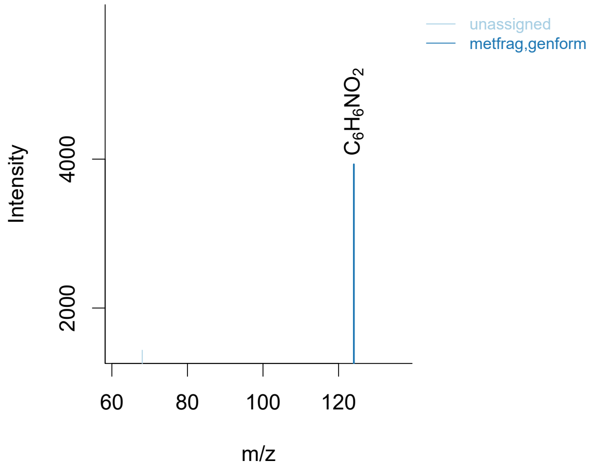

2.3.7 Feature ‘M174_R162_5656’

RT: 2.7 (Δ -4.8) min; m/z: 174.0218 (Δ -80.0379)

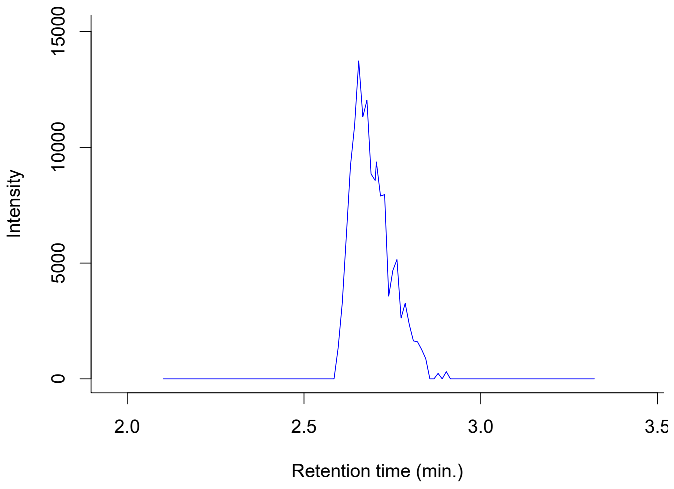

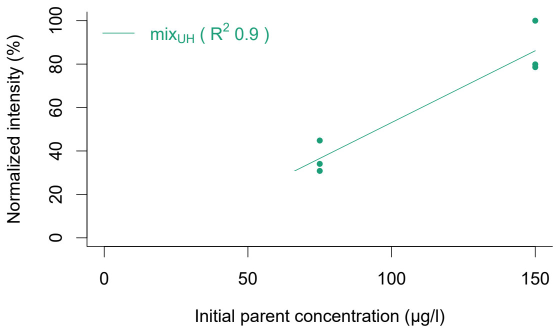

Condition	mix 25	mix 75	mix 150	RSQ	p	slope	single	dark
U		44%[4]	71%	0.45	0.143	0.36%		
UH		37%	86%	0.90	0.004	0.66%		
UHN								

2.3.7.1 Candidate ‘SuS-SMX-M174-4’

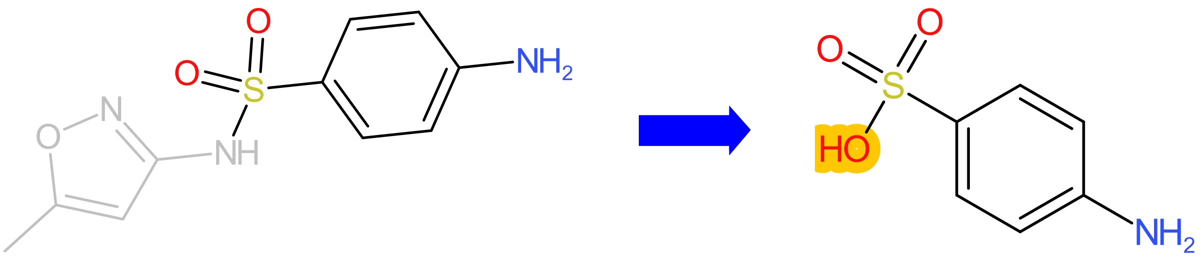

Formula	$C_6H_7NO_3S$ (Δ -$C_4H_4N_2$)
SMILES	Nc1ccc(cc1)S(O)(=O)=O
m/z error	-0.1 mDa
XLog P	-0.2 (Δ -1.4)
Data source(s)	CTS, BTE, LIT (a, b, c)
In silico similarity	0.00 (formula), 1.00 (compound)
ID confidence level	3a
Fit	$fit_{formula}$: 1.00<br>$fit_{compound}$: 0.91

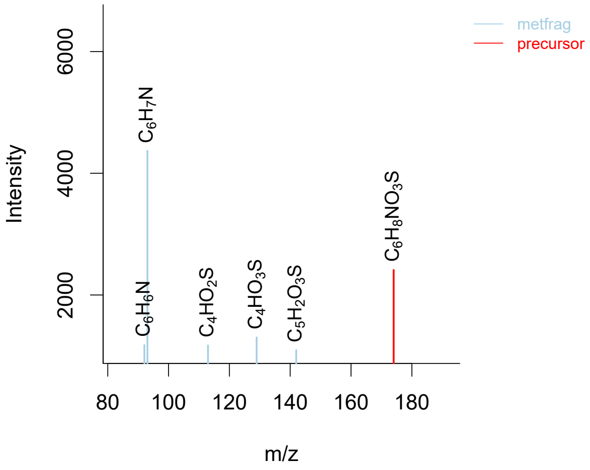

2.3.7.2 Candidate ‘SuS-SMX-M174-2’

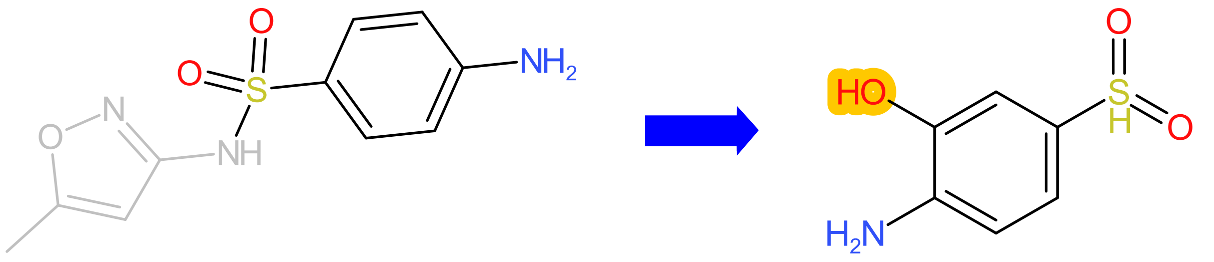

Formula	$C_6H_7NO_3S$ (Δ -$C_4H_4N_2$)
SMILES	Nc1ccc(S(=O)=O)cc1O
m/z error	-0.1 mDa
XLog P	0.2 (Δ -1.0)
Data source(s)	LIT (a)
In silico similarity	0.00 (formula), 1.00 (compound)
ID confidence level	3d
Fit	$fit_{formula}$: 1.00<br>$fit_{compound}$: 0.91

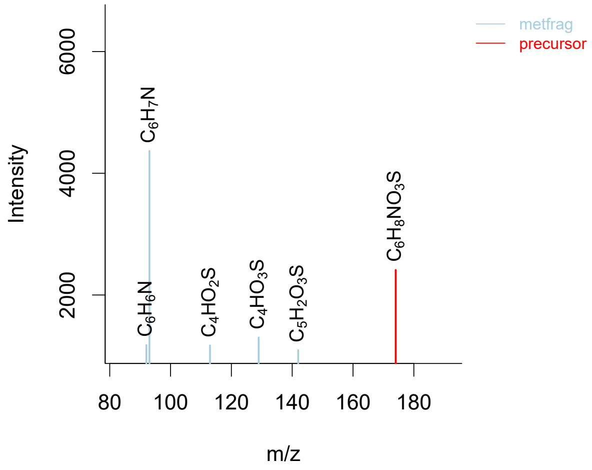

2.3.7.3 Candidate ‘SuS-SMX-M174-3’

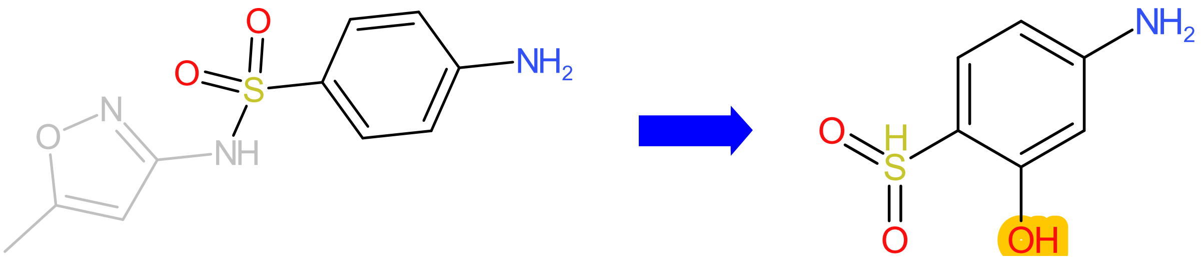

Formula	$C_6H_7NO_3S$ (Δ -$C_4H_4N_2$)
SMILES	Nc1ccc(S(=O)=O)c(O)c1
m/z error	-0.1 mDa
XLog P	0.6 (Δ -0.5)
Data source(s)	LIT (a)
In silico similarity	0.00 (formula), 1.00 (compound)
ID confidence level	3d
Fit	$fit_{formula}$: 1.00<br>$fit_{compound}$: 0.91

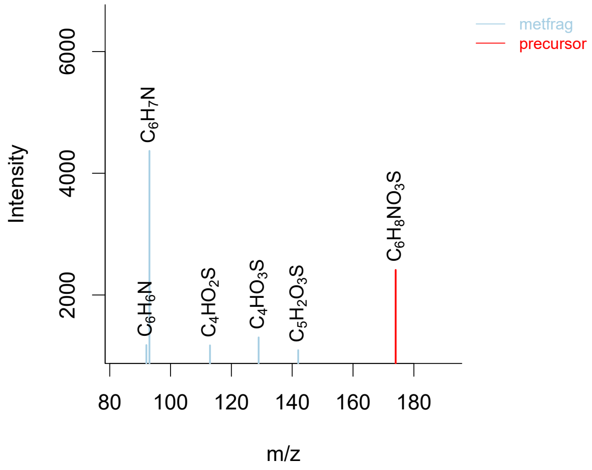

2.3.7.4 Candidate 'SuS-SMX-M174-1' (*DISPROVED*)

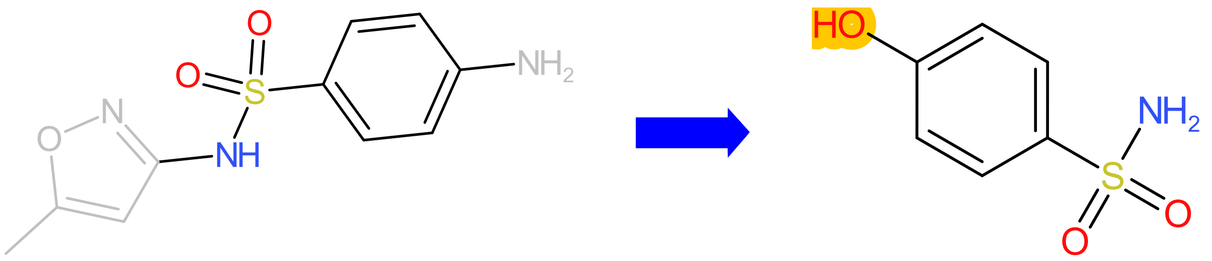

Formula	$C_6H_7NO_3S$ (Δ -$C_4H_4N_2$)
SMILES	NS(=O)(=O)c1ccc(O)cc1
m/z error	-0.1 mDa
XLog P	0.1 (Δ -1.1)
Data source(s)	LIT (a)
In silico similarity	0.00 (formula), 0.42 (compound)
ID confidence level	**disproved by standard**
Fit	$fit_{formula}$: 1.00<br>$fit_{compound}$: 0.91

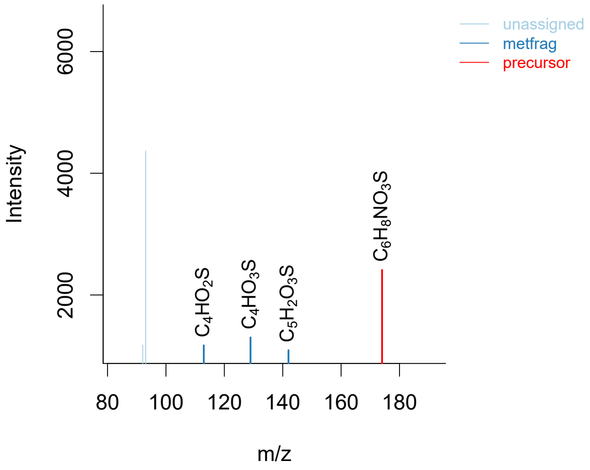

2.3.8 Feature 'M254_R349_3557'

RT: 5.8 (Δ -1.7) min; m/z: 254.0594 (Δ -0.0003)

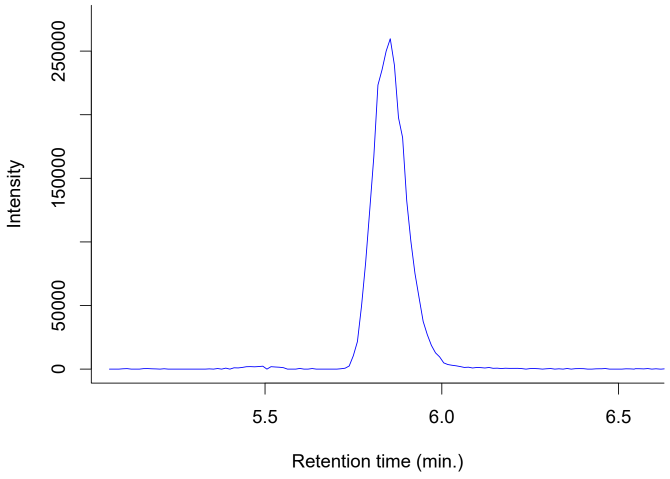

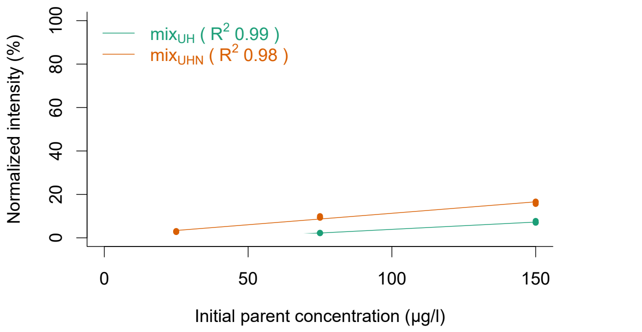

Condition	mix 25	mix 75	mix 150	RSQ	p	slope	single	dark
U			6%				93%	
UH		2%	7%	0.99	0.000	0.07%	48%	
UHN	3%	10%	16%	0.98	0.000	0.11%	48%	

2.3.8.1 Candidate 'SuS-SMX-M254-1'

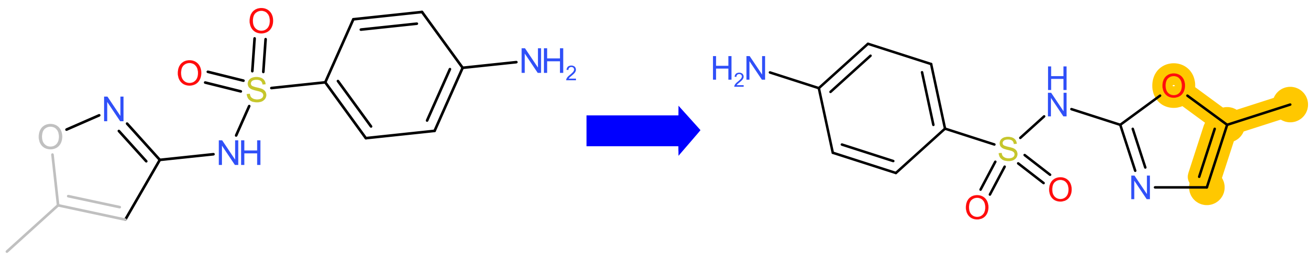

Formula	$C_{10}H_{11}N_3O_3S$ (no difference)
SMILES	Cc2cnc(NS(=O)(=O)c1ccc(N)cc1)o2
m/z error	+0.0 mDa
XLog P	0.5 (Δ -0.7)
Data source(s)	LIT (a)
In silico similarity	0.84 (formula), 0.79 (compound)
ID confidence level	3d
Fit	$fit_{formula}$: 1.00<br>$fit_{compound}$: 0.76

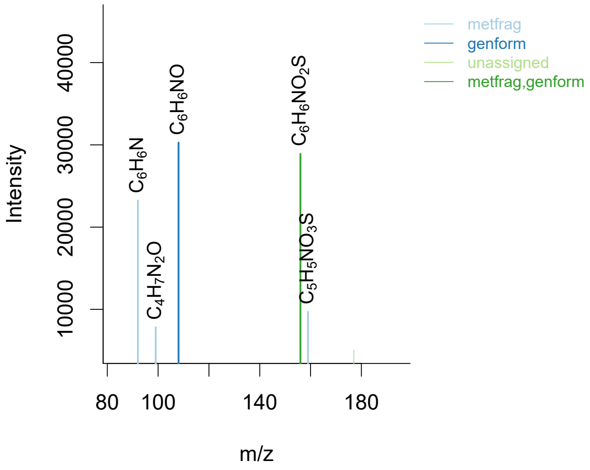

2.3.9 Feature ‘M272_R322_3885’

RT: 5.4 (Δ -2.1) min; m/z: 272.0700 (Δ +18.0103)

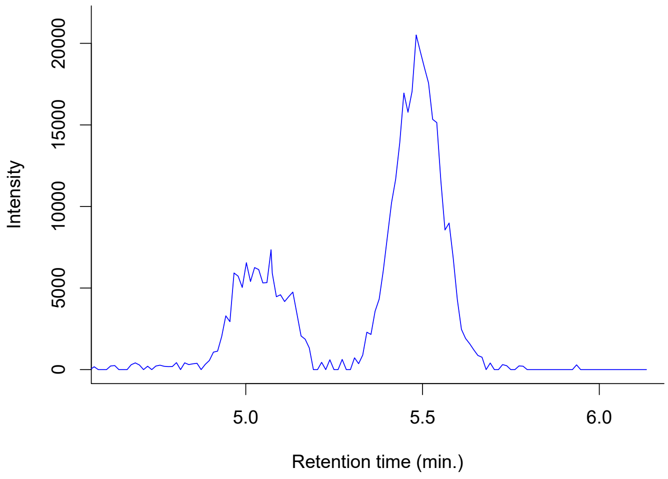

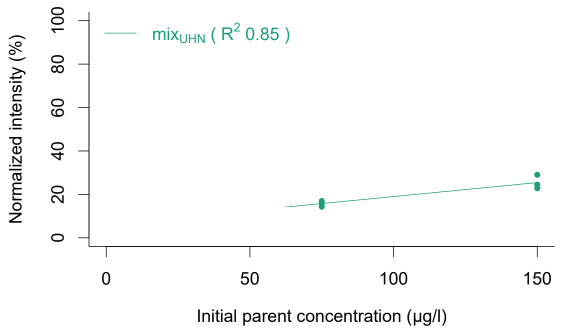

Condition	mix 25	mix 75	mix 150	RSQ	p	slope	single	dark
U		26%	17%	0.84	0.010	-0.12%	83%	
UH		22%	28%	0.07	0.623	0.07%	55%	
UHN		16%	25%	0.85	0.009	0.13%	47%	

Remarks: [7]

2.3.9.1 Candidate ‘SuS-SMX-M272-1’

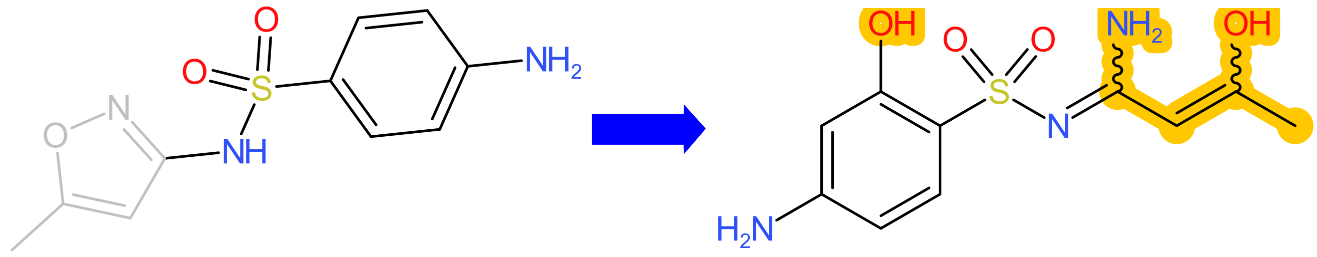

Formula	$C_{10}H_{13}N_3O_4S$ (Δ +H_2O)
SMILES	CC(=CC(=NS(=O)(=O)C1=C(C=C(C=C1)N)O)N)O
m/z error	+0.1 mDa
XLog P	0.8 (Δ -0.4)
Data source(s)	PC (168720538)
In silico similarity	0.88 (formula), 0.97 (compound)
ID confidence level	3d
Fit	$fit_{formula}$: 1.00<br>$fit_{compound}$: 0.65

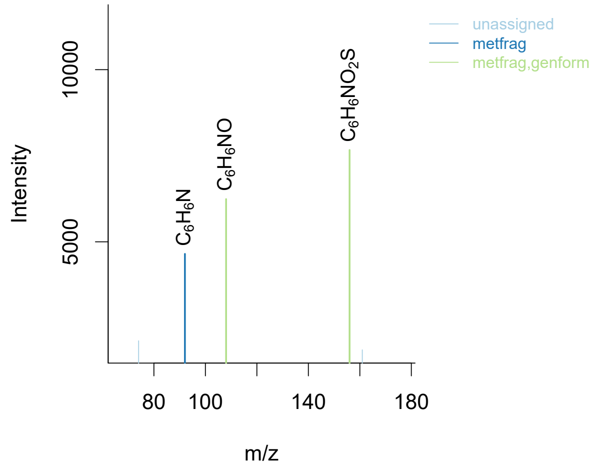

2.3.9.2 Candidate 'SuS-SMX-M272-2'

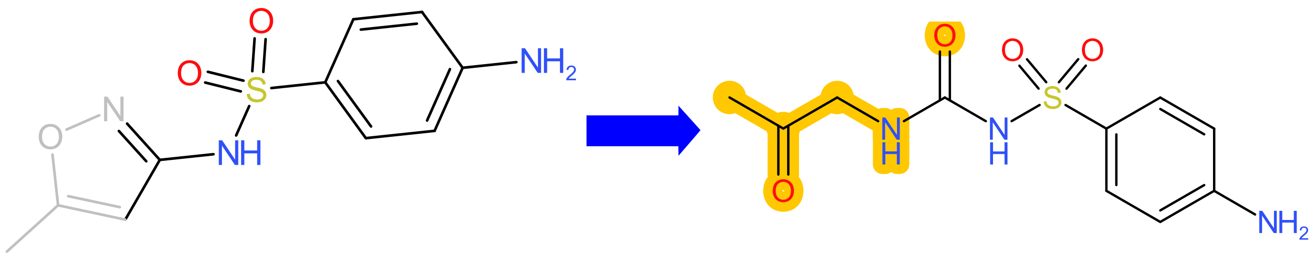

Formula	$C_{10}H_{13}N_3O_4S$ (Δ +H_2O)
SMILES	CC(=O)CNC(=O)NS(=O)(=O)c1ccc(N)cc1
m/z error	+0.1 mDa
XLog P	-0.8 (Δ -2.0)
Data source(s)	LIT (a)
In silico similarity	0.88 (formula), 0.82 (compound)
ID confidence level	3d
Fit	$fit_{formula}$: 1.00<br>$fit_{compound}$: 0.71

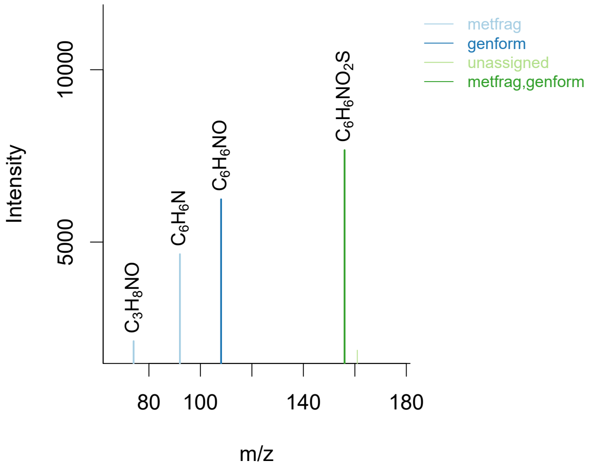

2.4 Parent 'phenazone'

2.4.1 Feature 'M94_R231_2478'

RT: 3.9 (Δ -3.7) min; m/z: 94.0645 (Δ -95.0385)

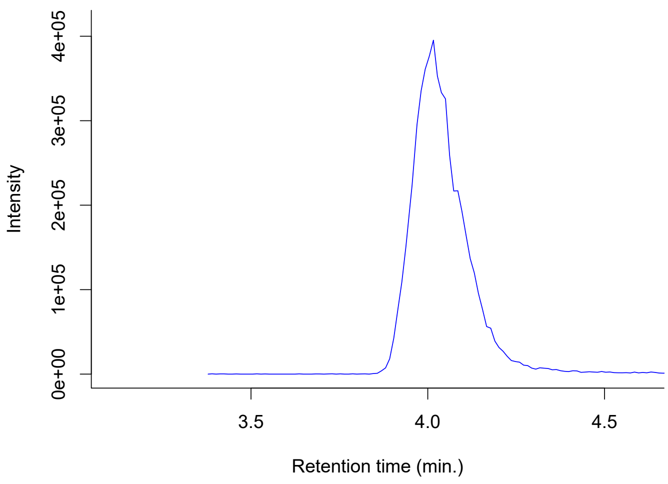

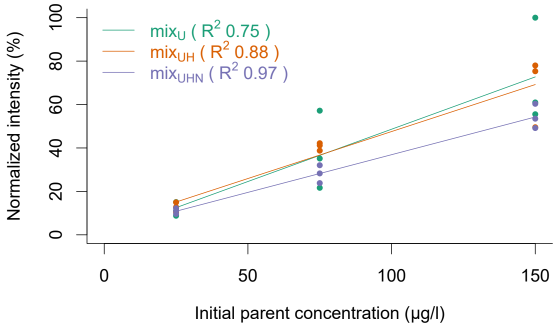

Condition	mix 25	mix 75	mix 150	RSQ	p	slope	single	dark
U	12%	38%	72%	0.75	0.003	0.48%	6%	
UH	13%	41%	68%	0.88	0.000	0.43%	12%	
UHN	11%	28%	54%	0.97	0.000	0.35%	12%	

2.4.1.1 Candidate 'SuS-PHE-M94-1'

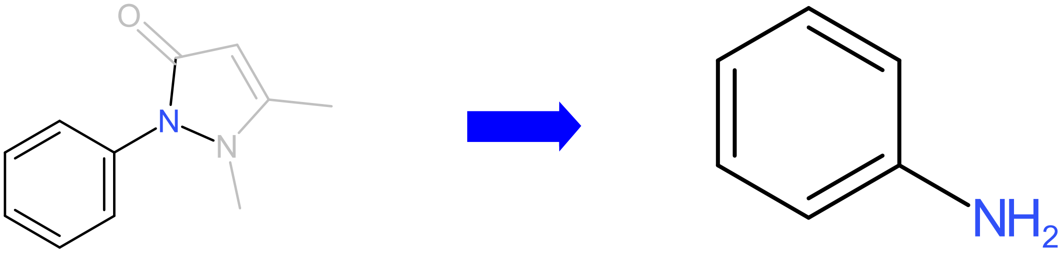

Formula	C_6H_7N (Δ $-C_5H_5NO$)
SMILES	Nc1ccccc1
m/z error	-0.7 mDa
XLog P	1.2 (Δ -0.1)
Data source(s)	LIT (a)
In silico similarity	NA (formula), 1.00 (compound)
ID confidence level	1
Fit	$fit_{formula}$: 1.00<br>$fit_{compound}$: 1.00

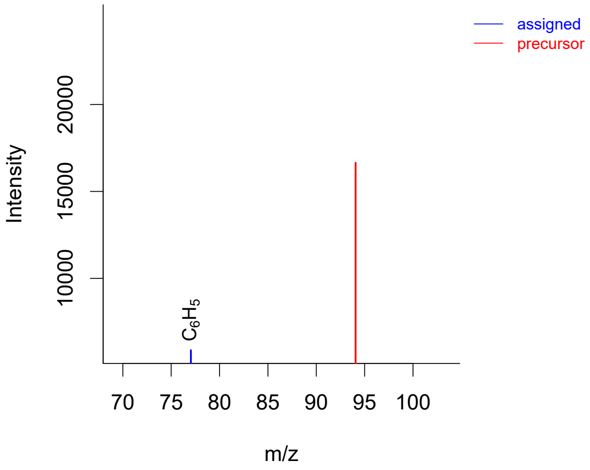

2.4.2 Feature 'M207_R424_3387'

RT: 7.1 (Δ -0.5) min; m/z: 207.1126 (Δ +18.0096)

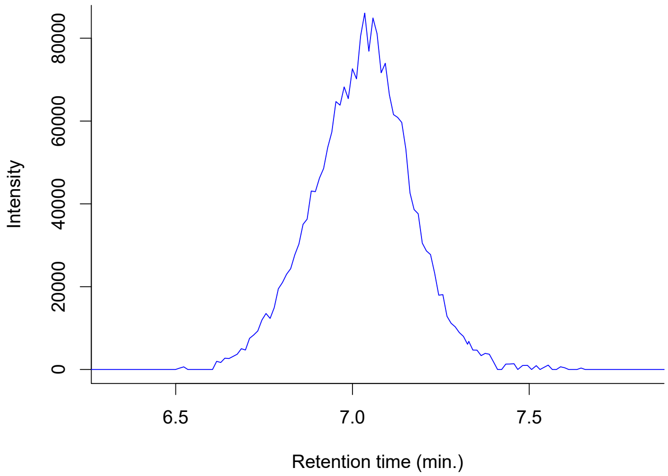

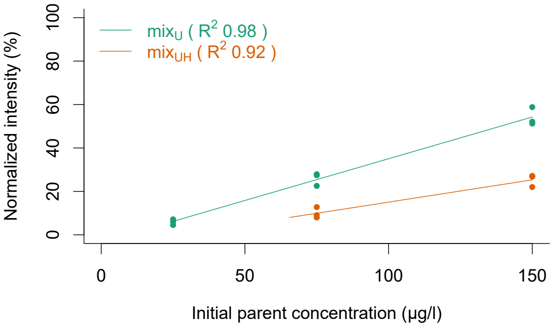

Condition	mix 25	mix 75	mix 150	RSQ	p	slope	single	dark
U	6%	26%	54%	0.98	0.000	0.38%	95%	
UH		10%	25%	0.92	0.002	0.21%	68%	
UHN			15%				64%	

2.4.2.1 Candidate 'SuS-PHE-M207-1'

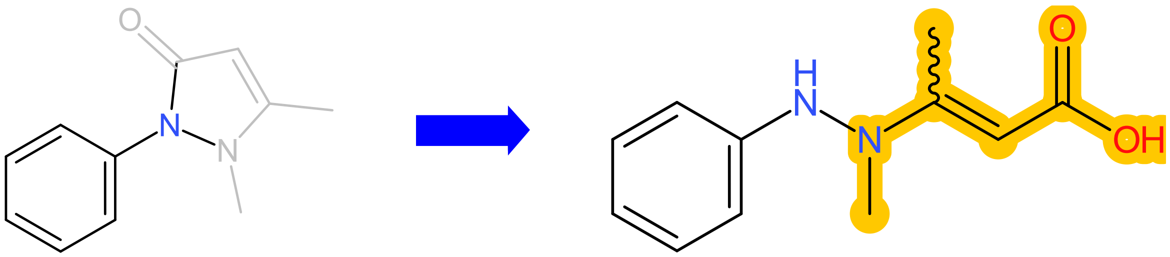

Formula	$C_{11}H_{14}N_2O_2$ (Δ +H_2O)
SMILES	CN(Nc1ccccc1)C(C)=CC(O)=O
m/z error	-0.3 mDa
XLog P	1.6 (Δ +0.3)
Data source(s)	CTS
In silico similarity	0.86 (formula), 0.80 (compound)
ID confidence level	3d
Fit	$fit_{formula}$: 1.00<br>$fit_{compound}$: 0.50

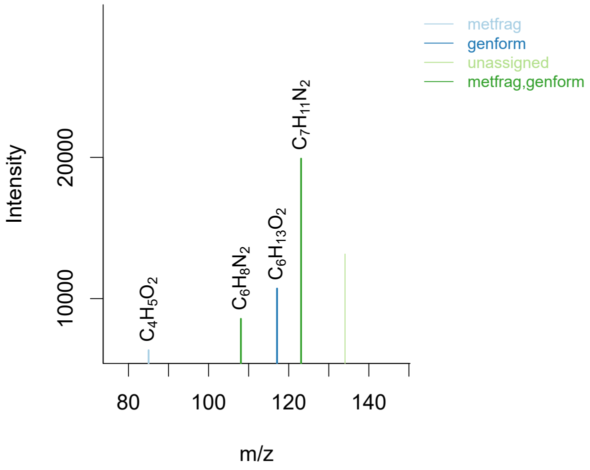

3 Candidates from formula suspect screening

3.1 Parent ‘metoprolol’

3.1.1 Feature ‘M302_R337_5079’

RT: 5.6 (Δ -1.2) min; m/z: 302.1964 (Δ +34.0046)

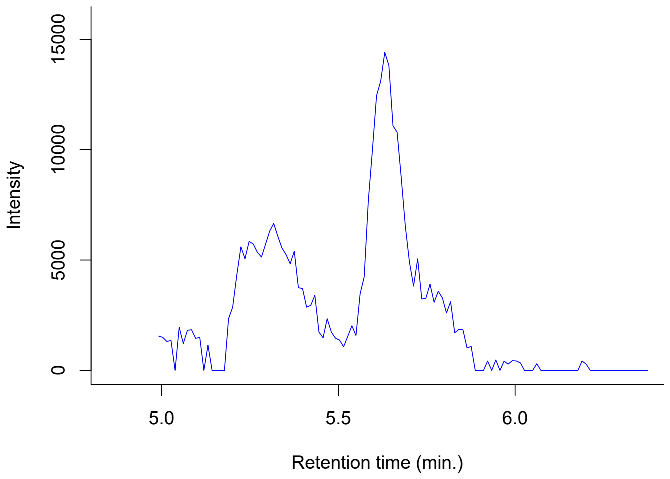

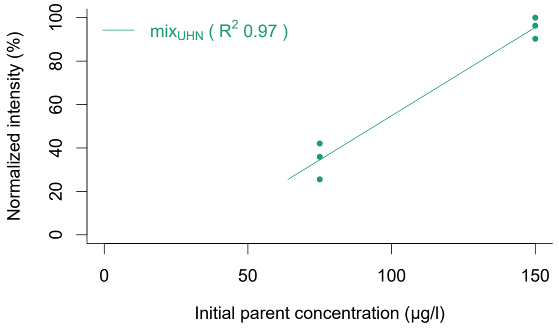

Condition	mix 25	mix 75	mix 150	RSQ	p	slope	single	dark
U								
UH		43%	69%[5]	0.44	0.153	0.34%	29%	
UHN		35%	96%	0.97	0.000	0.81%	49%	

3.1.1.1 Candidate ‘SuF-MET-M302-1’

$C_{15}H_{25}NO_3$ → $C_{15}H_{27}NO_5$

Formula	$C_{15}H_{27}NO_5$ (Δ +H_2O_2)
m/z error	+0.2 mDa
Data source(s)	-
In silico similarity	1.00 (formula)
ID confidence level	4b
Fit	$fit_{formula}$: 1.00
Other matches	M302_R316_5723

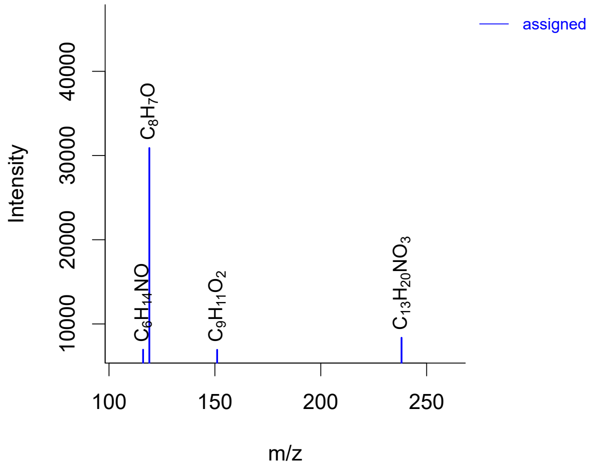

3.1.2 Feature ‘M302_R316_5723’

RT: 5.3 (Δ -1.6) min; m/z: 302.1964 (Δ +34.0047)

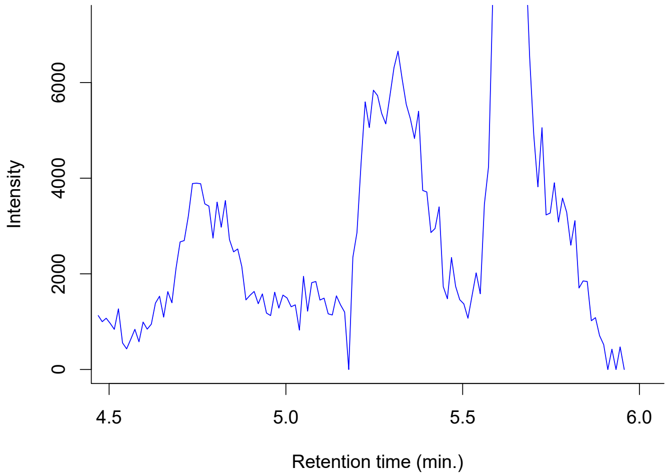

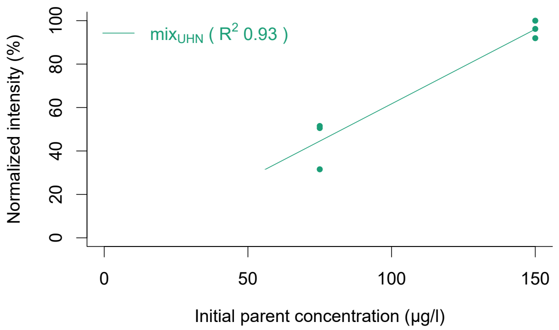

Condition	mix 25	mix 75	mix 150	RSQ	p	slope	single	dark
U								
UH			79%					
UHN		45%	96%	0.93	0.002	0.69%	66%	

3.1.2.1 Candidate ‘SuF-MET-M302-1’

$C_{15}H_{25}NO_3$ → $C_{15}H_{27}NO_5$

Formula	$C_{15}H_{27}NO_5$ (Δ +H_2O_2)
m/z error	+0.2 mDa
Data source(s)	-
In silico similarity	0.00 (formula)
ID confidence level	5
Fit	$fit_{formula}$: 1.00
Other matches	M302_R337_5079

No MS/MS annotations

4 Candidates for unknowns from compound annotations

4.1 Parent ‘sulfamethoxazole’

4.1.1 Feature ‘M140_R170_3386’

RT: 2.8 (Δ -4.7) min; m/z: 140.0164 (Δ -114.0433)

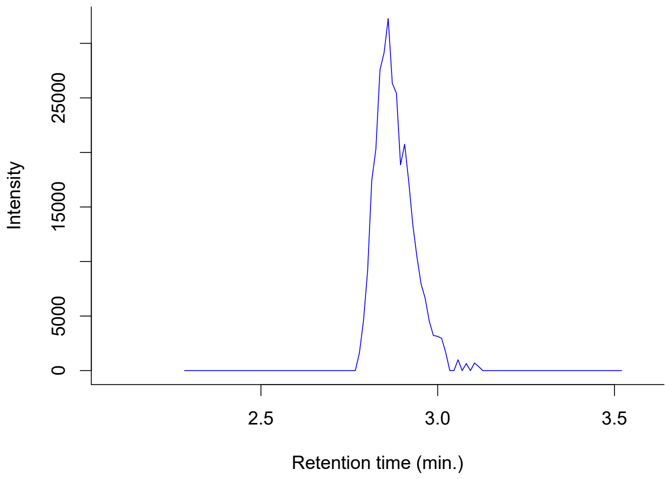

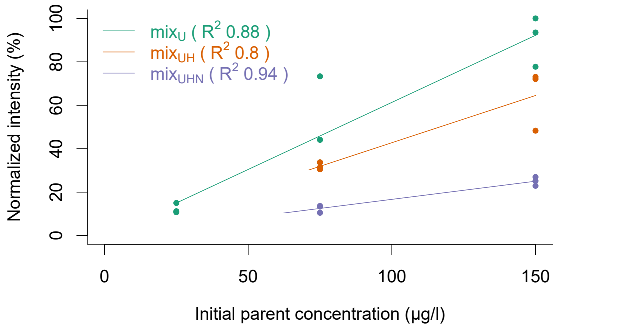

Condition	mix 25	mix 75	mix 150	RSQ	p	slope	single	dark
U	12%	50%	90%	0.88	0.000	0.62%	24%	
UH		32%	65%	0.80	0.016	0.43%	35%	
UHN		12%	25%	0.94	0.001	0.17%	37%	

4.1.1.1 Candidate ‘UnC-SMX-M140-1’

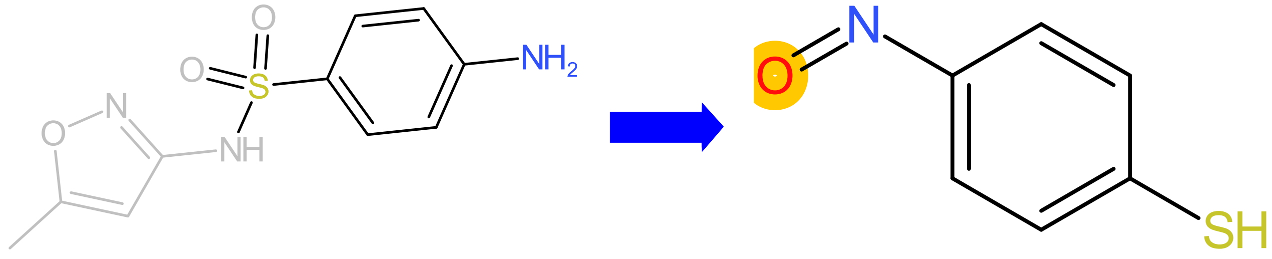

Formula	C_6H_5NOS (Δ $-C_4H_6N_2O_2$)
SMILES	C1=CC(=CC=C1N=O)S
m/z error	-0.0 mDa
XLog P	1.9 (Δ +0.8)
Data source(s)	19375293
In silico similarity	0.99 (formula), 1.00 (compound)
ID confidence level	3b
Fit	$fit_{formula}$: 1.00<br>$fit_{compound}$: 0.89
TP_sim_{max}	0.71
TP_score	$TP\_score_{compound}$: 1.89

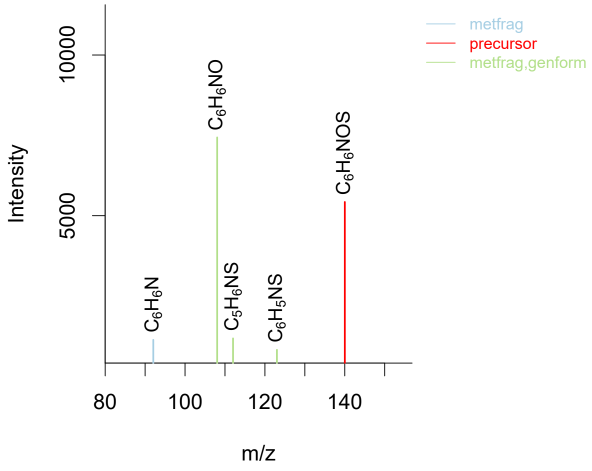

4.1.2 Feature ‘M190_R159_4149’

RT: 2.6 (Δ -4.8) min; m/z: 190.0166 (Δ -64.0431)

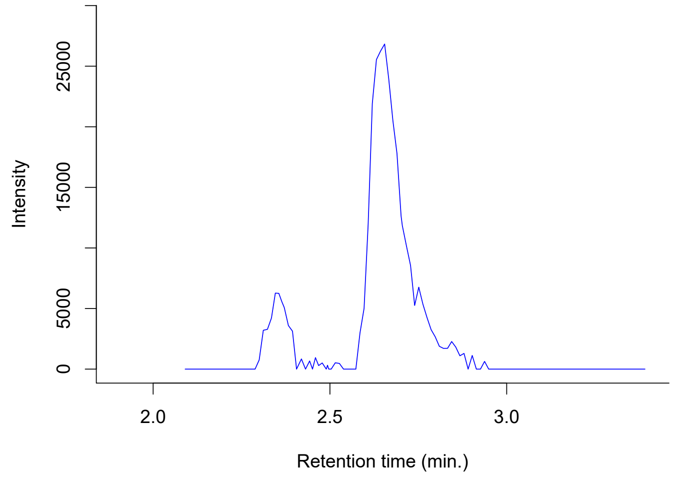

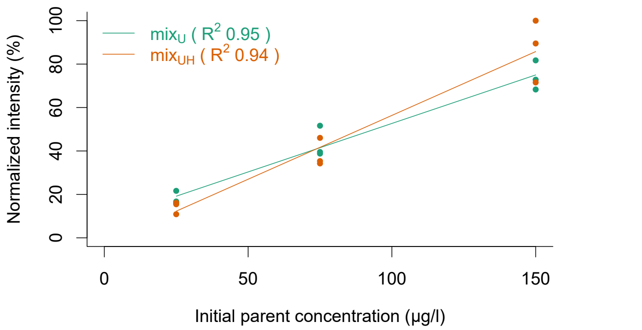

Condition	mix 25	mix 75	mix 150	RSQ	p	slope	single	dark
U	18%	43%	74%	0.95	0.000	0.45%	20%[8]	
UH	14%	39%	87%	0.94	0.000	0.59%	11%	
UHN								

4.1.2.1 Candidate ‘UnC-SMX-M190-1’

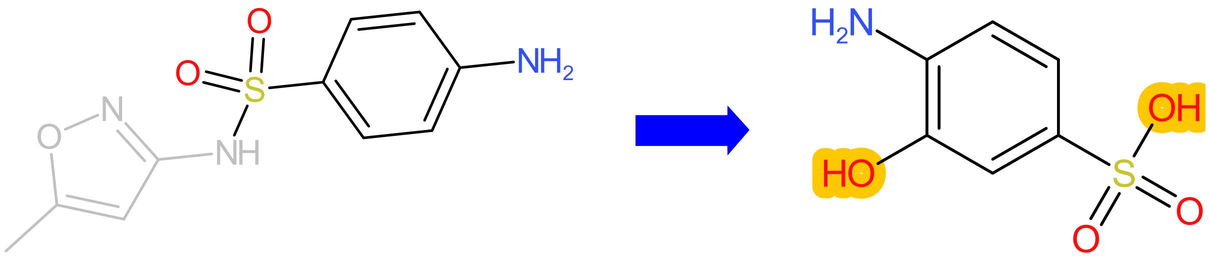

Formula	$C_6H_7NO_4S$ (Δ $-C_4H_4N_2$ +O)
SMILES	C1=CC(=C(C=C1S(=O)(=O)O)O)N
m/z error	-0.2 mDa
XLog P	-0.2 (Δ -1.4)
Data source(s)	18398311
In silico similarity	1.00 (formula), 0.92 (compound)
ID confidence level	3d
Fit	$fit_{formula}$: 0.95<br>$fit_{compound}$: 0.83
TP_sim$_{max}$	0.91
TP_score	$TP\_score_{compound}$: 1.82

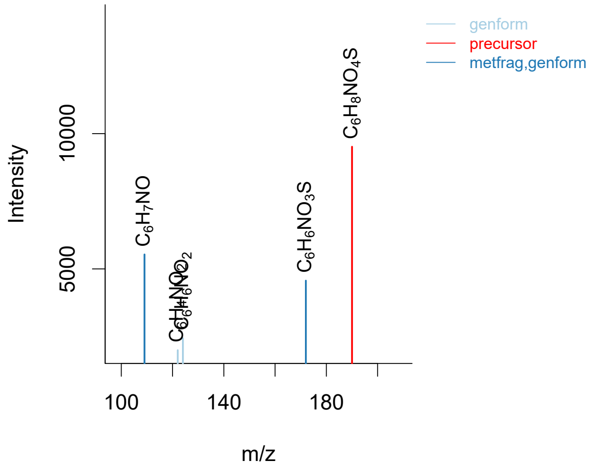

4.1.2.2 Candidate 'UnC-SMX-M190-2'

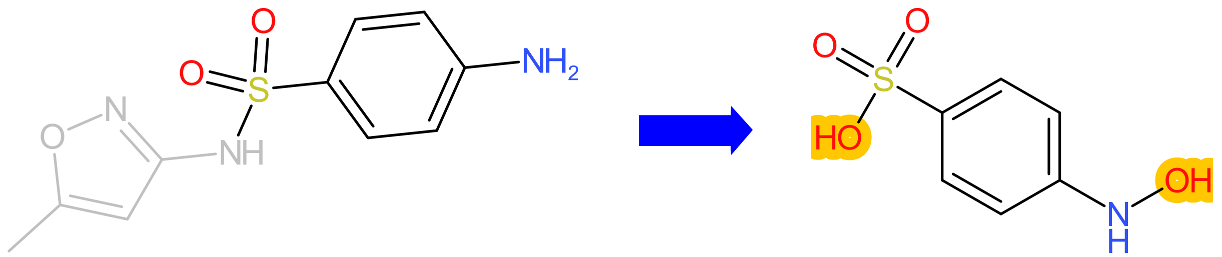

Formula	$C_6H_7NO_4S$ (Δ $-C_4H_4N_2$ +O)
SMILES	C1=CC(=CC=C1NO)S(=O)(=O)O
m/z error	-0.2 mDa
XLog P	-0.1 (Δ -1.3)
Data source(s)	89339163
In silico similarity	1.00 (formula), 0.92 (compound)
ID confidence level	3d
Fit	$fit_{formula}$: 0.95<br>$fit_{compound}$: 0.83
TP_sim_{max}	0.86
TP_score	$TP\_score_{compound}$: 1.78

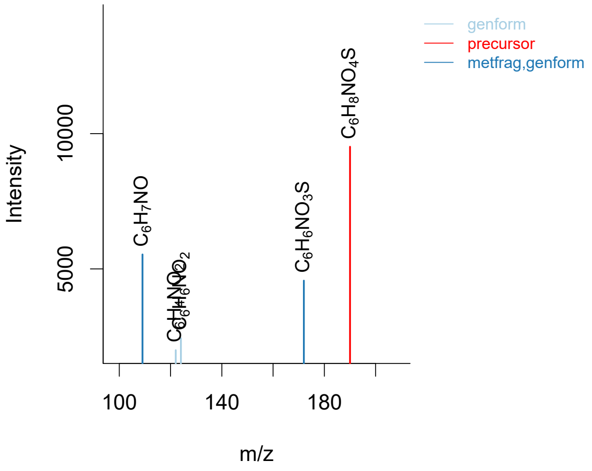

4.1.2.3 Candidate 'UnC-SMX-M190-3'

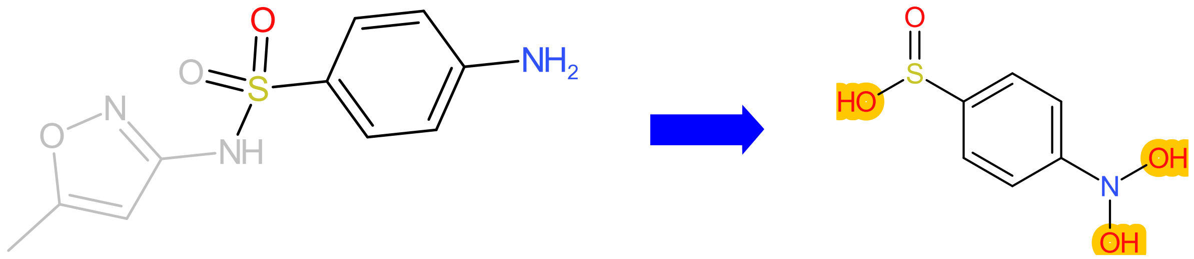

Formula	$C_6H_7NO_4S$ (Δ $-C_4H_4N_2$ +O)
SMILES	C1=CC(=CC=C1N(O)O)S(=O)O
m/z error	-0.2 mDa
XLog P	-2.0 (Δ -3.2)
Data source(s)	89191511
In silico similarity	1.00 (formula), 0.97 (compound)
ID confidence level	3d
Fit	$fit_{formula}$: 0.95<br>$fit_{compound}$: 0.75
TP_sim_{max}	0.82
TP_score	$TP\_score_{compound}$: 1.79

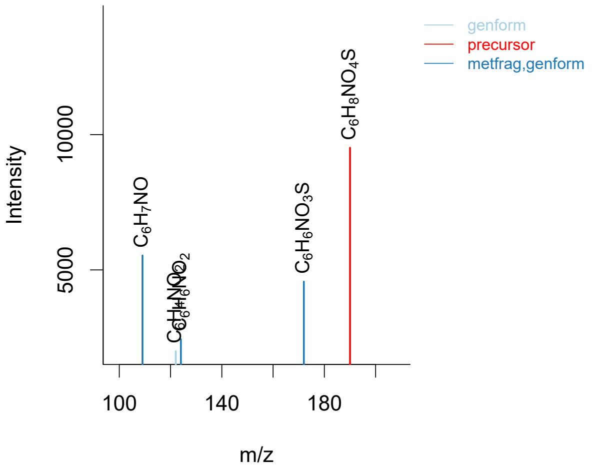

4.2 Parent ‘phenazone’

4.2.1 Feature ‘M176_R533_3000’

RT: 8.9 (Δ +1.3) min; m/z: 176.0703 (Δ -13.0326)

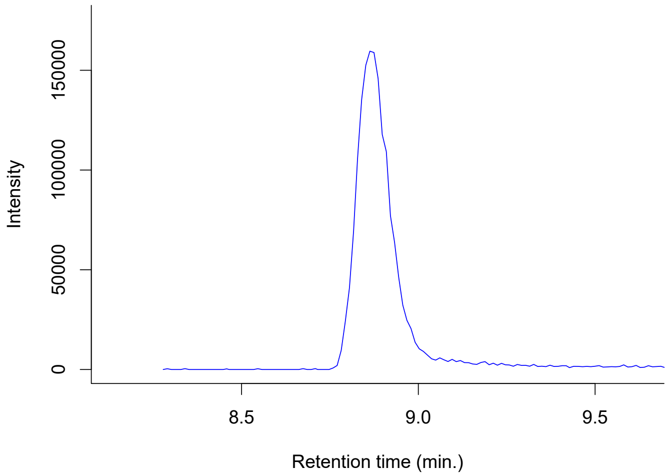

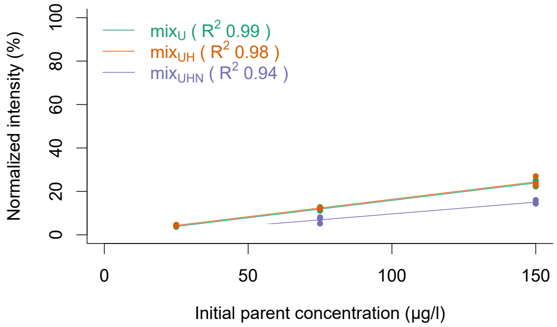

Condition	mix 25	mix 75	mix 150	RSQ	p	slope	single	dark
U	4%	12%	24%	0.99	0.000	0.16%	88%	
UH	4%	12%	24%	0.98	0.000	0.16%	88%	
UHN		7%	15%	0.94	0.001	0.11%	78%	

4.2.1.1 Candidate ‘UnC-PHE-M176-1’

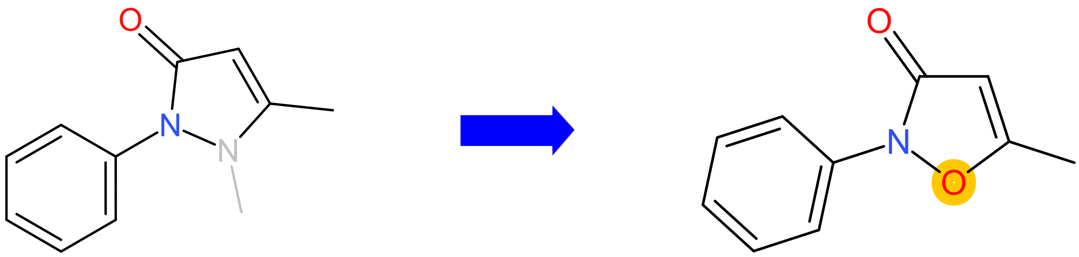

Formula	$C_{10}H_9NO_2$ (Δ -CH_3N +O)
SMILES	CC1=CC(=O)N(O1)C2=CC=CC=C2
m/z error	-0.3 mDa
XLog P	1.5 (Δ +0.1)
Data source(s)	5240302
In silico similarity	0.98 (formula), 0.90 (compound)
ID confidence level	3d
Fit	$fit_{formula}$: 0.95<br>$fit_{compound}$: 0.92
TP_sim_{max}	0.38
TP_score	$TP\_score_{compound}$: 1.82

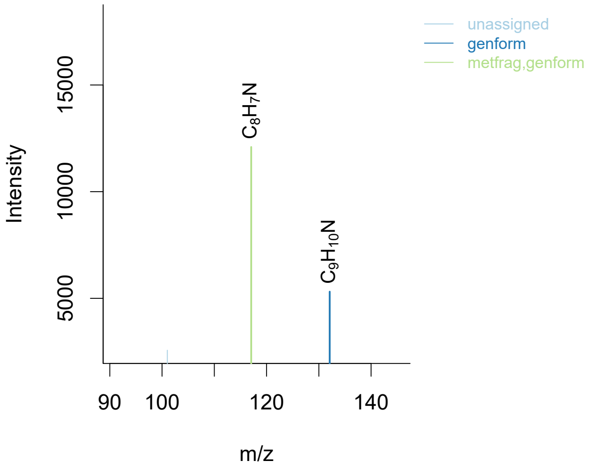

4.2.2 Feature ‘M219_R515_7445’

RT: 8.6 (Δ +1.0) min; m/z: 219.1137 (Δ +30.0107)

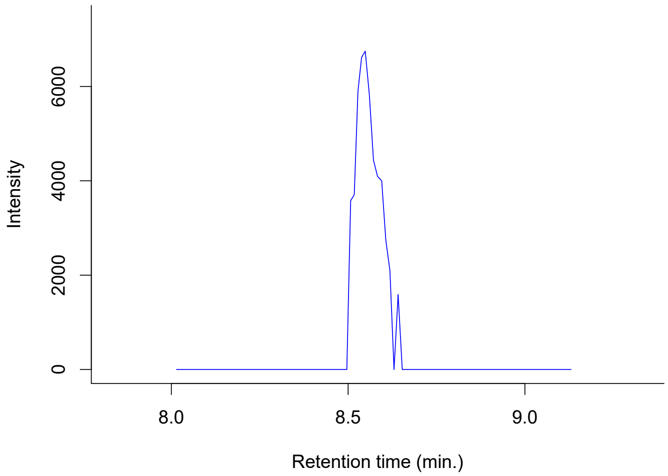

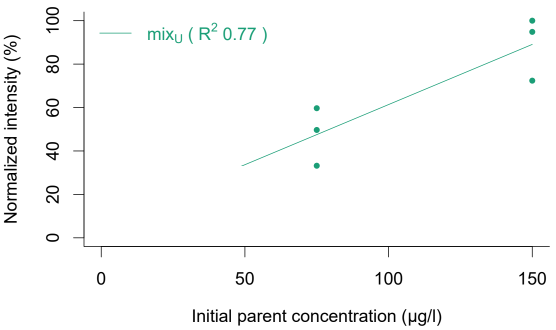

Condition	mix 25	mix 75	mix 150	RSQ	p	slope	single	dark
U		48%	89%	0.77	0.022	0.55%		
UH								
UHN								

4.2.2.1 Candidate ‘UnC-PHE-M219-1’

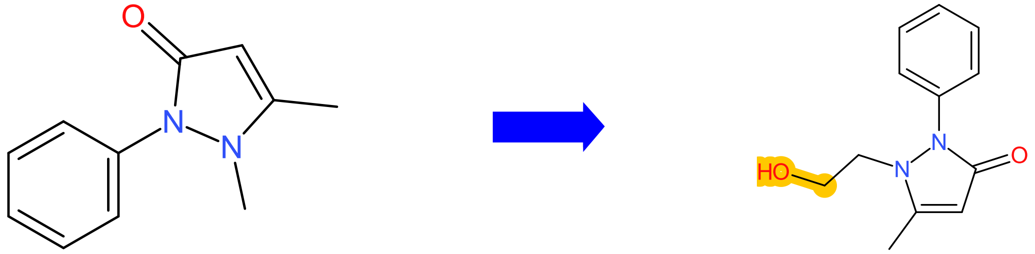

Formula	$C_{12}H_{14}N_2O_2$ (Δ +CH_2O)
SMILES	CC1=CC(=O)N(N1CCO)C2=CC=CC=C2
m/z error	+0.9 mDa
XLog P	0.6 (Δ -0.7)
Data source(s)	118332457
In silico similarity	NA
ID confidence level	5
Fit	$fit_{formula}$: 1.00<br>$fit_{compound}$: 1.00
TP_sim_{max}	0.88
TP_score	$TP\_score_{compound}$: 1.00

No MS/MS annotations

4.2.3 Feature 'M375_R598_4727'

RT: 10.0 (Δ +2.4) min; m/z: 375.1813 (Δ +186.0784)

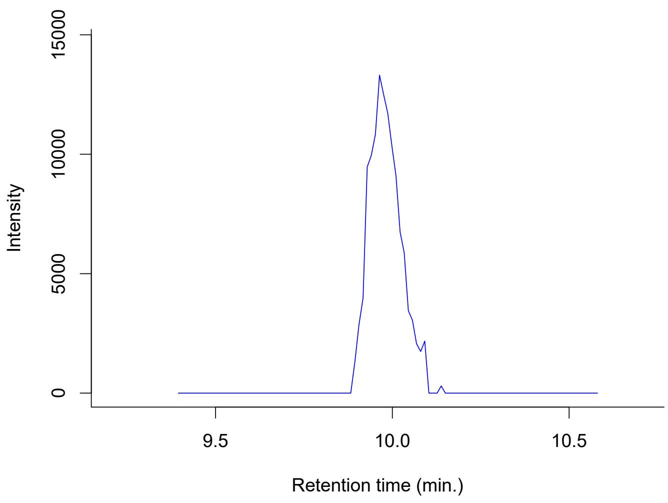

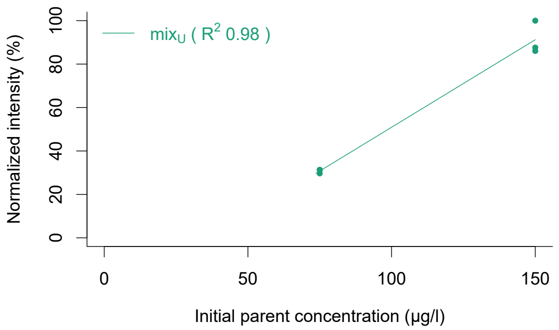

Condition	mix 25	mix 75	mix 150	RSQ	p	slope	single	dark
U		31%	91%	0.98	0.000	0.81%	55%	
UH			53%				33%	
UHN			36%				32%	

4.2.3.1 Candidate 'UnC-PHE-M375-1' (*DISPROVED*)

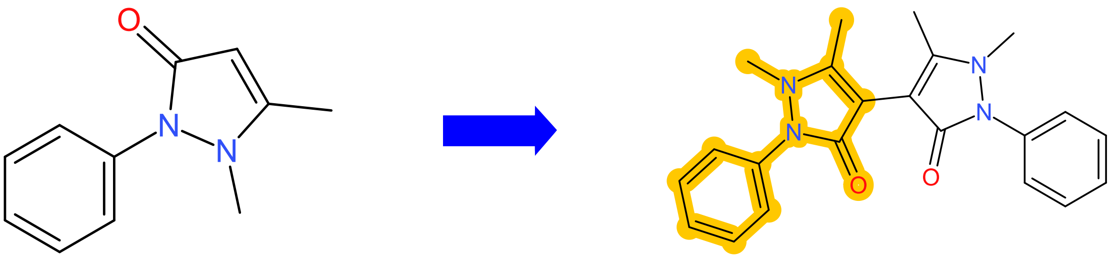

Formula	$C_{22}H_{22}N_4O_2$ (Δ +$C_{11}H_{10}N_2O$)
SMILES	CC1=C(C(=O)N(N1C)C2=CC=CC=C2)C3=C(N(N(C3=O)C4=CC=CC=C4)C)C
m/z error	-0.3 mDa
XLog P	2.4 (Δ +1.1)
Data source(s)	10604
In silico similarity	1.00 (formula), 1.00 (compound)
ID confidence level	**disproved by standard**
Fit	$fit_{formula}$: 1.00<br>$fit_{compound}$: 1.00
TP_sim_{max}	0.59
TP_score	$TP\_score_{compound}$: 2.00

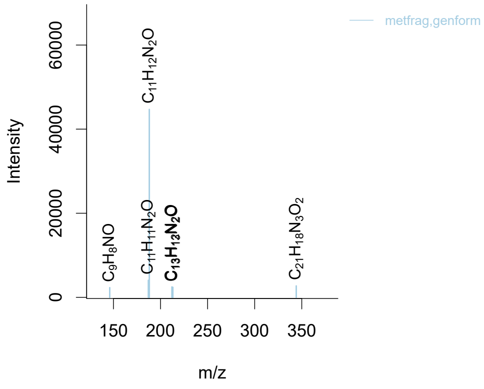

5 Candidates for unknowns from formula annotations

5.1 Parent 'flecainide'

5.1.1 Feature 'M166_R473_6824'

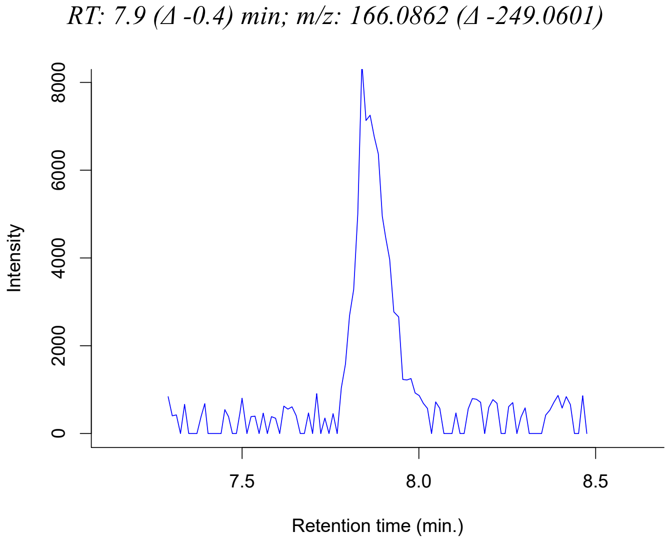

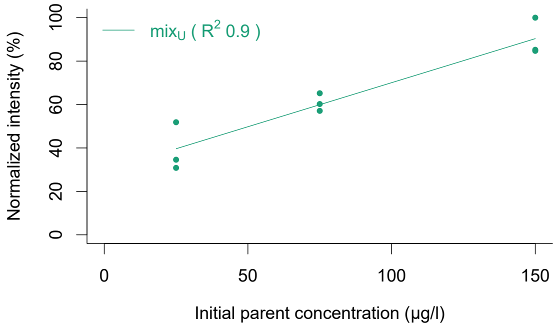

Condition	mix 25	mix 75	mix 150	RSQ	p	slope	single	dark
U	39%	61%	90%	0.90	0.000	0.41%		
UH								
UHN								

5.1.1.1 Candidate 'UnF-FLE-M166-1' (*DISPROVED*)

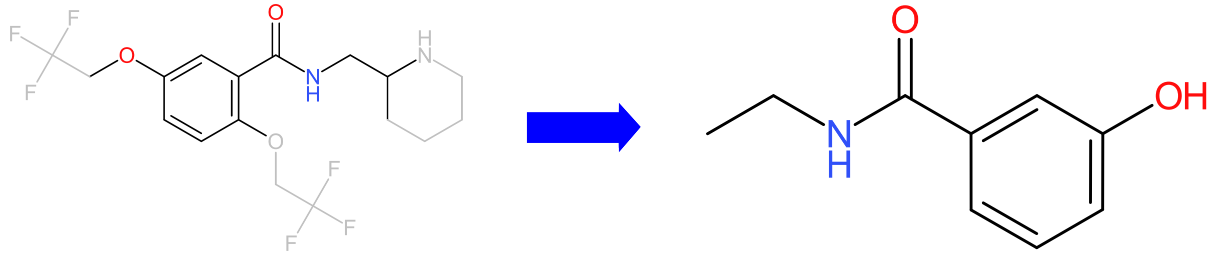

Formula	$C_9H_{11}NO_2$ (Δ -$C_8H_9NOF_6$)
SMILES	CCNC(=O)C1=CC(=CC=C1)O
m/z error	-0.1 mDa
XLog P	1.4 (Δ -3.2)
Data source(s)	-
In silico similarity	NA
ID confidence level	**disproved by standard**
Fit	$fit_{formula}$: 1.00<br>$fit_{compound}$: 1.00
TP_sim_{max}	0.71
TP_score	$TP\_score_{formula}$: 1.00<br>$TP\_score_{compound}$: 1.00

No MS/MS annotations

5.2 Parent 'phenazone'

5.2.1 Feature 'M122_R471_3775'

RT: 7.9 (Δ +0.3) min; m/z: 122.0598 (Δ -67.0431)

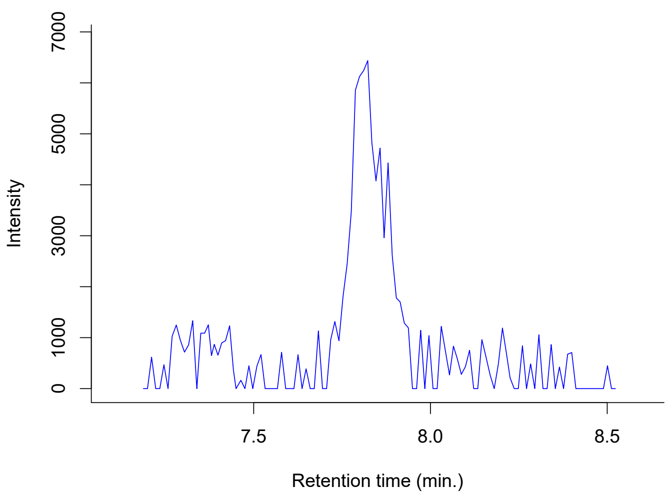

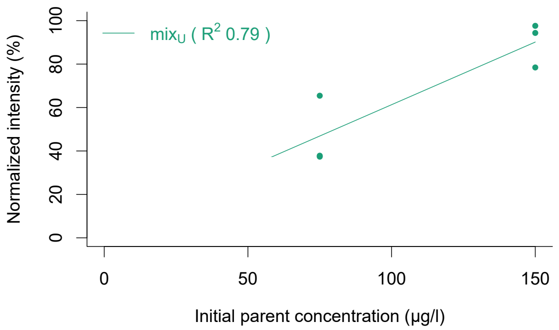

Condition	mix 25	mix 75	mix 150	RSQ	p	slope	single	dark
U		47%	90%	0.79	0.017	0.58%	61%	
UH[4]		75%	90%	0.44	0.151	0.2%	57%	
UHN			60%				68%	

5.2.1.1 Candidate 'UnF-PHE-M122-1'

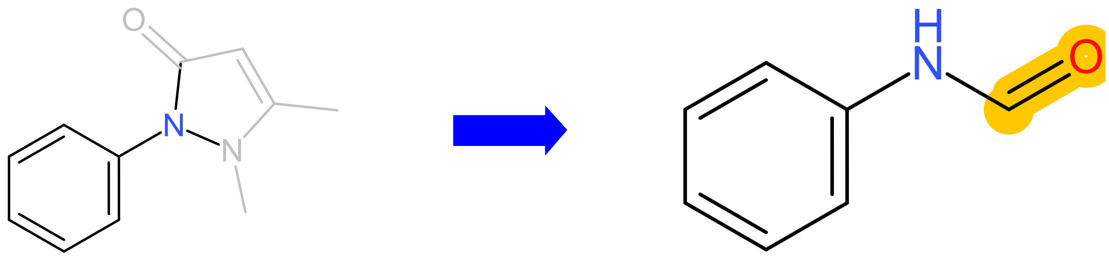

Formula	C_7H_7NO (Δ $-C_4H_5N$)
SMILES	C1=CC=C(C=C1)NC=O
m/z error	-0.3 mDa
XLog P	1.0 (Δ -0.3)
Data source(s)	7671
In silico similarity	0.00 (formula), 0.00 (compound)
ID confidence level	1
Fit	$fit_{formula}$: 1.00<br>$fit_{compound}$: 0.78
TP_sim$_{max}$	0.58
TP_score	$TP\_score_{formula}$: 1.00<br>$TP\_score_{compound}$: 0.78

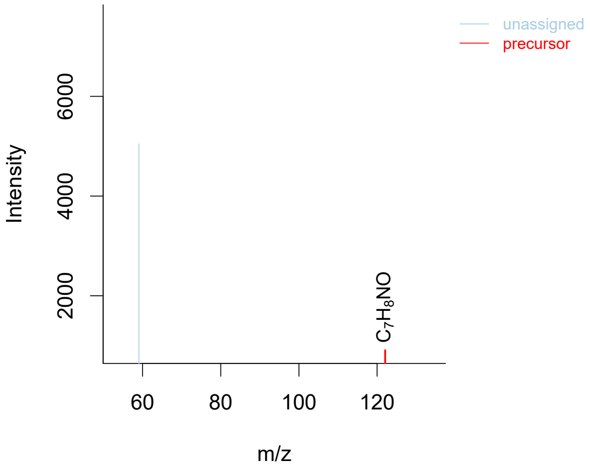

5.2.2 Feature 'M149_R515_2917'

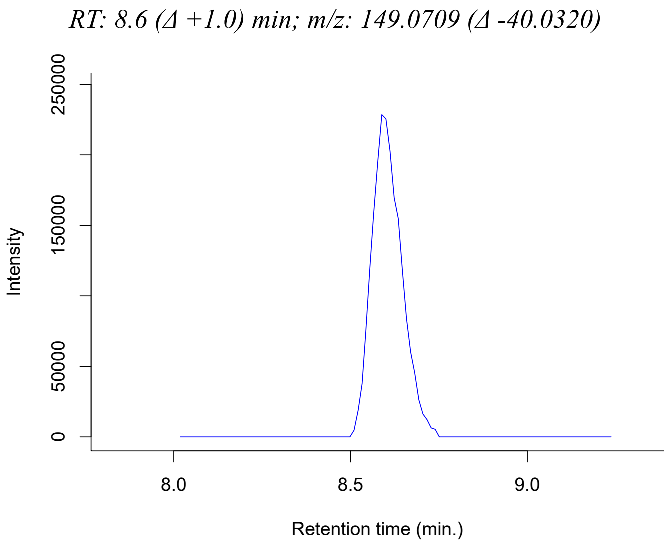

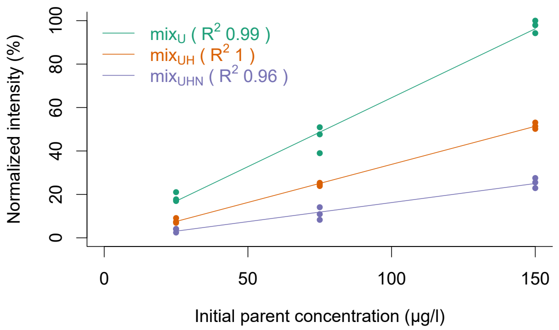

Condition	mix 25	mix 75	mix 150	RSQ	p	slope	single	dark
U	19%	46%	97%	0.99	0.000	0.64%	29%	
UH	8%	25%	52%	1.00	0.000	0.35%	36%	
UHN	4%	11%	25%	0.96	0.000	0.18%	32%	

5.2.2.1 Candidate 'UnF-PHE-M149-1'

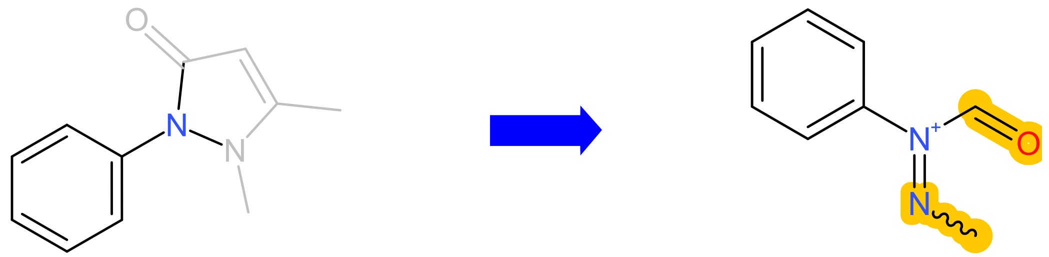

Formula	$C_8H_9N_2O$ (Δ -C_3H_3)
SMILES	CN=[N+](C=O)C1=CC=CC=C1
m/z error	-0.1 mDa
XLog P	1.4 (Δ +0.1)
Data source(s)	-
In silico similarity	NA (formula), 0.97 (compound)
ID confidence level	3b
Fit	$fit_{formula}$: 1.00<br>$fit_{compound}$: 0.64
TP_sim_{max}	0.39
TP_score	$TP\_score_{formula}$: 1.00<br>$TP\_score_{compound}$: 1.61

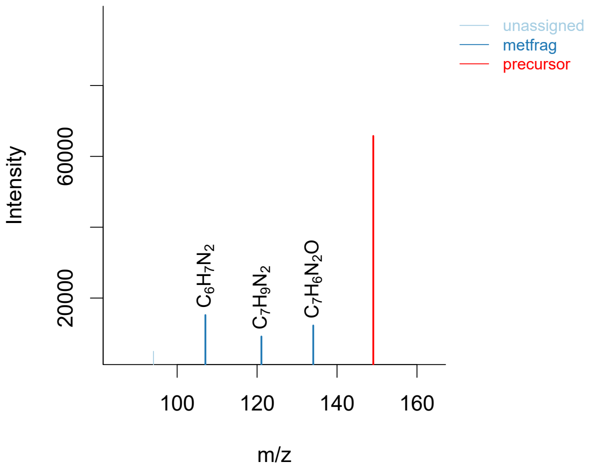

5.2.3 Feature ‘M150_R528_2607’

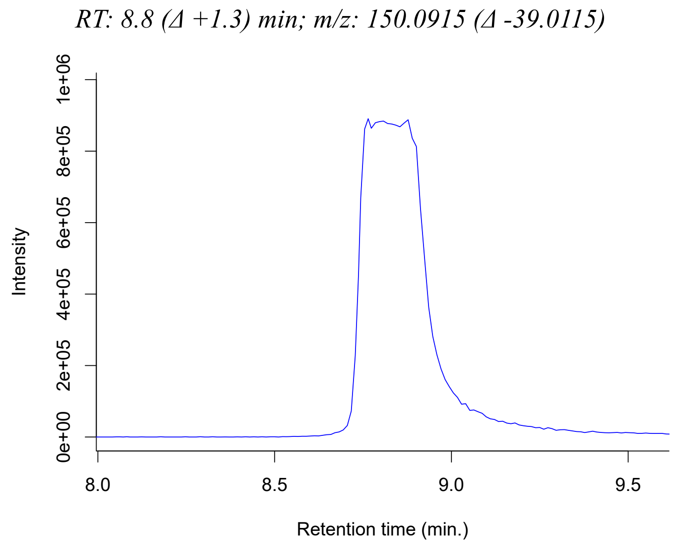

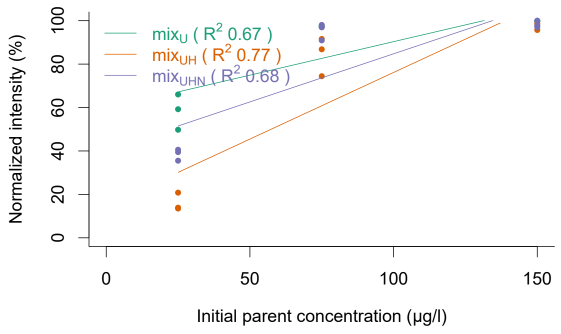

Condition	mix 25	mix 75	mix 150	RSQ	p	slope	single	dark
U	58%	97%	100%	0.67	0.007	0.31%	97%	
UH	16%	84%	97%	0.77	0.002	0.61%	95%	
UHN	39%	95%	98%	0.68	0.006	0.44%	98%	

5.2.3.1 Candidate ‘UnF-PHE-M150-1’ (*DISPROVED*)

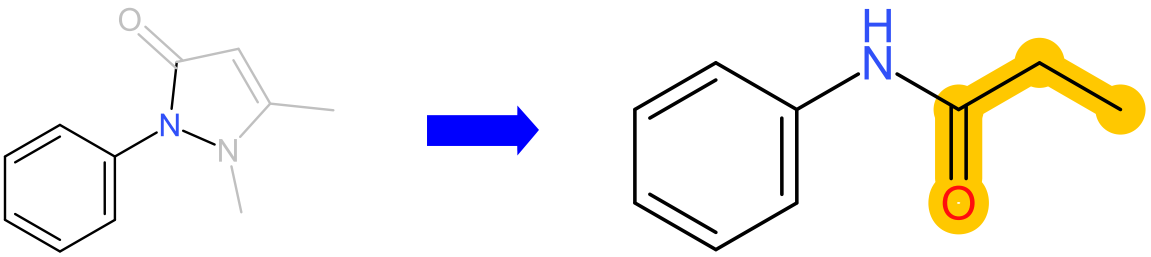

Formula	$C_9H_{11}NO$ (Δ -C_2HN)
SMILES	CCC(=O)NC1=CC=CC=C1
m/z error	+0.1 mDa
XLog P	1.5 (Δ +0.2)
Data source(s)	12107
In silico similarity	0.97 (formula), 0.98 (compound)
ID confidence level	**disproved by standard**
Fit	$fit_{formula}$: 1.00<br>$fit_{compound}$: 0.64
TP_sim_{max}	0.52
TP_score	$TP\_score_{formula}$: 1.97<br>$TP\_score_{compound}$: 1.62

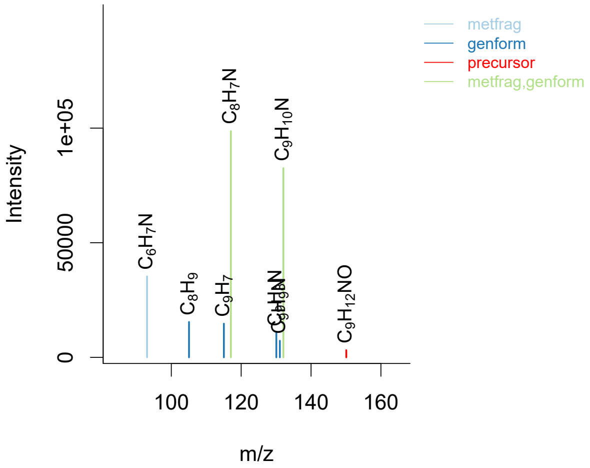

5.2.4 Feature ‘M165_R462_6907’

RT: 7.7 (Δ +0.1) min; m/z: 165.0654 (Δ -24.0375)

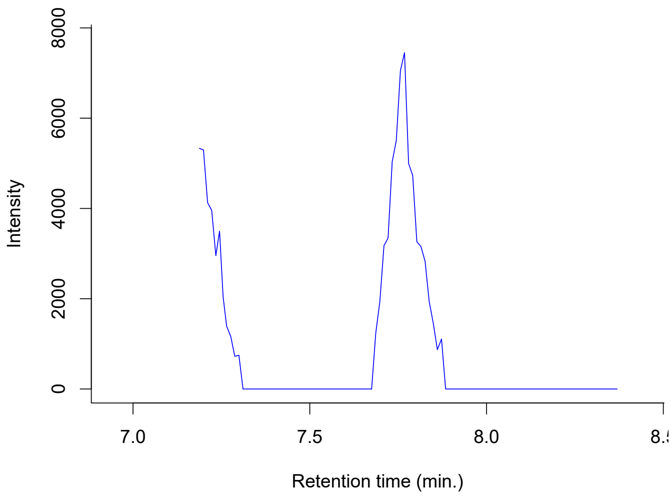

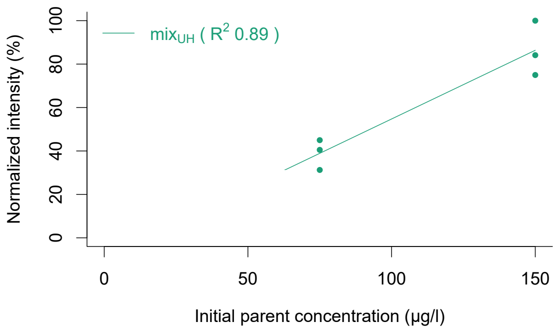

Condition	mix 25	mix 75	mix 150	RSQ	p	slope	single	dark
U								
UH		39%	86%	0.89	0.005	0.63%		
UHN			47%					

5.2.4.1 Candidate ‘UnF-PHE-M165-1’

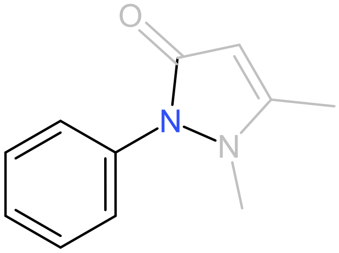

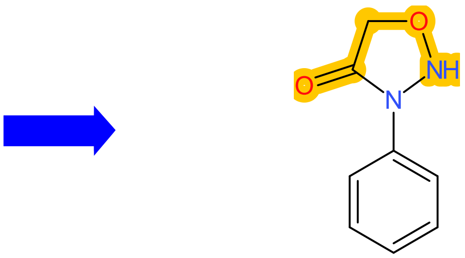

Formula	$C_8H_8N_2O_2$ (Δ $-C_3H_4$ $+O$)
SMILES	O=C1CONN1c2ccccc2
m/z error	-0.4 mDa
XLog P	1.4 (Δ +0.0)
Data source(s)	-
In silico similarity	NA
ID confidence level	4c
Fit	$fit_{formula}$: 0.95<br>$fit_{compound}$: 0.58
TP_sim_{max}	0.53
TP_score	$TP\_score_{formula}$: 0.95<br>$TP\_score_{compound}$: 0.58

No MS/MS annotations

5.2.5 Feature ‘M166_R473_6824’

RT: 7.9 (Δ +0.3) min; m/z: 166.0862 (Δ -23.0167)

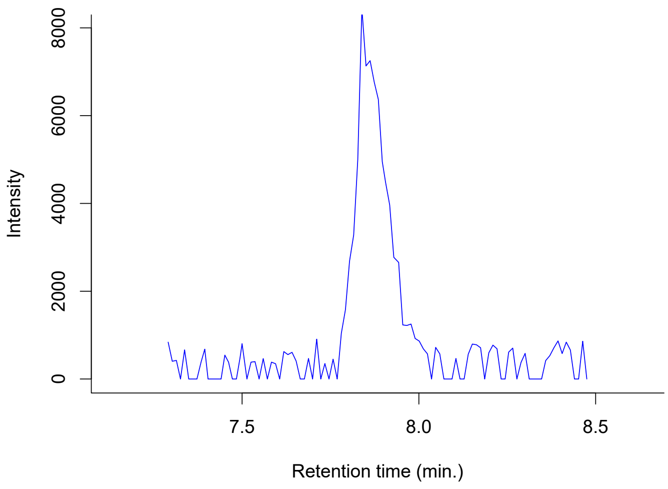

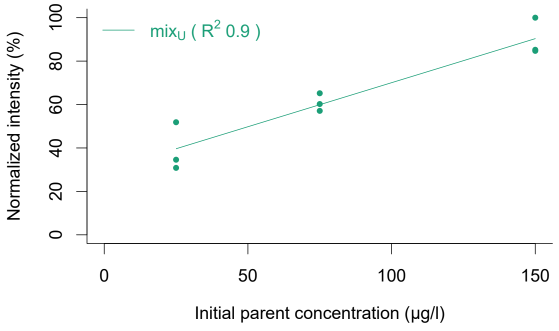

Condition	mix 25	mix 75	mix 150	RSQ	p	slope	single	dark
U	39%	61%	90%	0.90	0.000	0.41%		
UH								
UHN								

5.2.5.1 Candidate ‘UnF-PHE-M166-1’

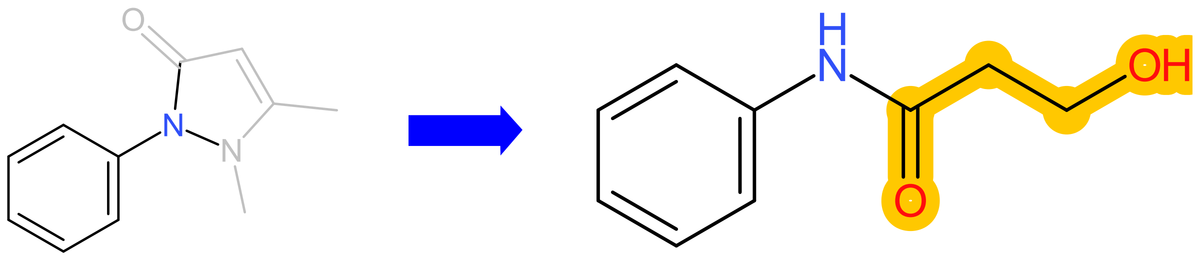

Formula	$C_9H_{11}NO_2$ (Δ $-C_2HN$ +O)
SMILES	C1=CC=C(C=C1)NC(=O)CCO
m/z error	-0.1 mDa
XLog P	0.8 (Δ -0.5)
Data source(s)	15561474
In silico similarity	NA
ID confidence level	5
Fit	$fit_{formula}$: 0.96<br>$fit_{compound}$: 0.58
TP_sim_{max}	0.46
TP_score	$TP\_score_{formula}$: 0.96<br>$TP\_score_{compound}$: 0.58

No MS/MS annotations

5.2.6 Feature 'M208_R533_2918'

RT: 8.9 (Δ +1.3) min; m/z: 208.0965 (Δ +18.9936)

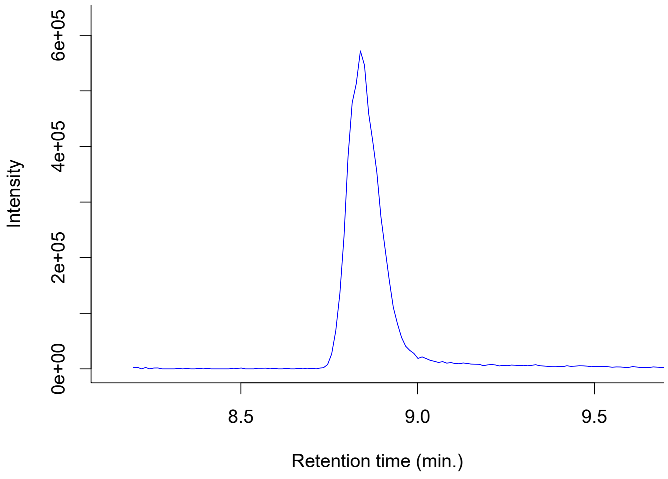

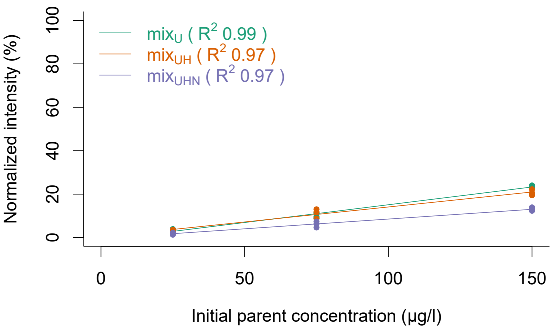

Condition	mix 25	mix 75	mix 150	RSQ	p	slope	single	dark
U	3%	10%	24%	0.99	0.000	0.16%	87%	
UH	3%	11%	21%	0.97	0.000	0.14%	96%	
UHN	2%	6%	13%	0.97	0.000	0.09%	87%	

5.2.6.1 Candidate 'UnF-PHE-M208-1'

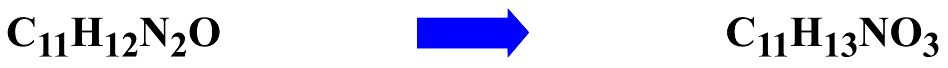

Formula	$C_{11}H_{13}NO_3$ (Δ -N +HO_2)
m/z error	-0.3 mDa
Data source(s)	-
In silico similarity	0.99 (formula)
ID confidence level	4b
Fit	$fit_{formula}$: 0.96
$TP\_score_{formula}$	1.95

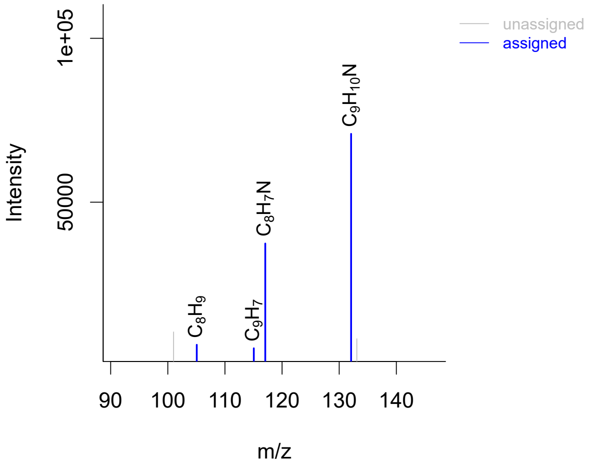

References

(1) Xie, Y. *Knitr: A General-Purpose Package for Dynamic Report Generation in R*; 2022.

(2) Xie, Y. *Dynamic Documents with R and Knitr*, 2nd ed.; Chapman; Hall/CRC: Boca Raton, Florida, 2015.

(3) Xie, Y. Knitr: A Comprehensive Tool for Reproducible Research in R. In *Implementing reproducible computational research*; Stodden, V., Leisch, F., Peng, R. D., Eds.; Chapman; Hall/CRC, 2014.

(4) Allaire, J.; Xie, Y.; Dervieux, C.; McPherson, J.; Luraschi, J.; Ushey, K.; Atkins, A.; Wickham, H.; Cheng, J.; Chang, W.; Iannone, R. *Rmarkdown: Dynamic Documents for r*; 2023.

(5) Xie, Y.; Allaire, J. J.; Grolemund, G. *R Markdown: The Definitive Guide*; Chapman; Hall/CRC: Boca Raton, Florida, 2018.

(6) Xie, Y.; Dervieux, C.; Riederer, E. *R Markdown Cookbook*; Chapman; Hall/CRC: Boca Raton, Florida, 2020.

(7) Xie, Y.; Lesur, R.; Thorne, B.; Tan, X. *Pagedown: Paginate the HTML Output of r Markdown with CSS for Print*.

(8) Google Chrome Webbrowser. https://www.google.com/chrome/.

(9) Helmus, R.; Bagdonaite, I.; Voogt, P. de; Bommel, M. van; Schymanski, E. L.; Wezel, A. van; Laak, T. ter. Code accompanying the manuscript "accompanies the manuscript "Comprehensive mass spectrometry workflows to systematically elucidate transformation processes of organic micropollutants: a case study on photodegradation of four pharmaceuticals", 2024. https://doi.org/10.5281/zenodo.13377935.

(10) Guha, R. Chemical Informatics Functionality in R. *Journal of Statistical Software***2007**, *18* (6). https://doi.org/10.18637/jss.v018.i05.

(11) Kim, S.; Thiessen, P. A.; Bolton, E. E.; Chen, J.; Fu, G.; Gindulyte, A.; Han, L.; He, J.; He, S.; Shoemaker, B. A.; Wang, J.; Yu, B.; Zhang, J.; Bryant, S. H. PubChem Substance and Compound Databases. *Nucleic Acids Research***2016**, *44* (D1), D1202–D1213. https://doi.org/10.1093/nar/gkv951.
